# Global trend and disparity in the burden of ischemic stroke attributable to high body-mass index from 1990 to 2021 and projection to 2050: a systematic analysis based on the Global Burden of Disease Study 2021

**DOI:** 10.1016/j.dialog.2026.100304

**Published:** 2026-04-22

**Authors:** Shuting Ni, Ying Zhang, Shilei Lin, Yongxiang Zhang, Tong Zhao

**Affiliations:** a Department of Neurosurgery, Neurosurgery Research Institute, The First Affiliated Hospital, Fujian Medical University, Fuzhou 350005, Fujian, China; b Department of Anesthesiology, Anesthesiology Research Institute, The First Affiliated Hospital of Fujian Medical University, National Regional Medical Center, Binhai Campus of the First Affiliated Hospital, Fujian Medical University, Fuzhou, China; c Department of Neurosurgery, Binhai Branch of National Regional Medical Center, The First Affiliated Hospital, Fujian Medical University, Fuzhou 350209, Fujian, China; d Fujian Provincial Institutes of Brain Disorders and Brain Sciences, First Affiliated Hospital, Fujian Medical University, Fuzhou 350005, Fujian, China

**Keywords:** Ischemic stroke, High body-mass index, Global Burden of Disease Study 2021, Disability-adjusted life years, Estimated annual percentage change

## Abstract

**Purpose:**

This study assessed the global burden, trends, and socioeconomic disparities of ischemic stroke attributable to high body-mass index (IS-HBMI) from 1990 to 2021, and to project future trends to 2050.

**Methods:**

Data were extracted from the Global Burden of Disease Study 2021 (GBD 2021; data accessed on [November 8, 2025]). The analysis focused on ischemic stroke  attributable to high body-mass index, measuring deaths, disability-adjusted life years (DALYs), and their corresponding age-standardized rates (ASMR and ASDR)  across all ages, both sexes, and all Socio-demographic Index (SDI) regions. Temporal trends were analyzed using Joinpoint regression (estimated annual percentage change, EAPC), and drivers of mortality change were quantified via demographic decomposition. A Bayesian age-period-cohort (BAPC) model projected future burden to 2050. Regional performance was benchmarked using a best-performance frontier analysis.

**Conclusion:**

The study revealed that the global disease burden of IS-HBMI had continuously increased from 1990 to 2021, and it was predicted to escalate until 2050. The findings emphasize the need for more detailed IS screening and weight loss measures tailored to specific regions and populations, which would benefit efforts to curb the projected rise in IS-HBMI deaths.

## 1 Introduction

Ischemic stroke is a neurological dysfunction caused by a vascular accident and is an important contributor to disability and death [1]. According to the latest data, there are approximately 7.8 million new cases of IS worldwide each year, accounting for 62.4 % of all stroke cases worldwide [2], [3]. IS triggers a complex series of ischemic cascade reactions, ultimately resulting in irreversible neuronal damage and cerebral infarction. This process can result in devastating dysfunction for the individual patient, as well as a heavy financial and caregiving burden on his or her family and society. The rapid progression of ischemic stroke can be attributed to a number of factors, the main risk factor being hypertension [4]. In addition, many studies have highlighted risk factors such as high cholesterol, diabetes, obesity, and end-stage renal disease [5], [6], [7], [8], [9]. Many of these risk factors are strongly associated with weight gain [10].

In recent years, more and more studies have focused on the relationship between obesity/overweight and IS, emphasizing that obesity/overweight is an important risk factor for the development of IS. The pathophysiological mechanisms linking high BMI to IS are multifactorial and well-established. Elevated BMI contributes to a pro-inflammatory state, insulin resistance, dyslipidemia, endothelial dysfunction, and increased incidence of hypertension and type 2 diabetes, all of which accelerate atherosclerosis and promote thrombus formation, ultimately elevating the risk of cerebral ischemia [11], [12]. While other risk factors like smoking and alcohol use are critical, the global pandemic of obesity, its role as an upstream driver of multiple metabolic comorbidities, and the standardized availability of BMI data across populations make it a pivotal and actionable target for global preventive strategies [10].Stroke risk rises signified that from 1990 to 2019, the EAPC for IS mortality (ASMR) attributable to high BMI was 1.62 (95cantly when BMI >25 kg/m^2^, a study suggests [13].A prospective cohort study also found a significant association between high BMI and perioperative ischemic stroke [14].Other studies, including Mendelian Randomization analyses and meta-analyses, have further reinforced the significant correlation between obesity/overweight and the development of IS [15].

Although previous studies have revealed an association between obesity/overweight and IS, there is still a lack of comprehensive study on the burden of ischemic stroke attributable to high body-mass index (IS-HBMI) and its trends across sexes, age groups, regions, and countries/territories. This study comprehensively utilized data from the Global Burden of Disease Study 2021 (GBD 2021) to analyze the trends and disparities in the burden of IS-HBMI from 1990 to 2021 and predicted its future trajectory up to 2050, which was expected to provide a scientific foundation for future targeted prevention and intervention strategies. This work directly contributes to monitoring progress towards United Nations Sustainable Development Goal (SDG) 3.4, on reducing premature mortality from non-communicable diseases, and SDG 10, on reducing inequalities within and among countries.

## 2 Methods

The GBD 2021, led by the Institute for Health Metrics and Evaluation (IHME) at the University of Washington, employs standardized and comparable methods to assess disease burden data across 204 countries and regions, covering 371 diseases and injuries and 88 risk factors, providing a comprehensive framework for global health comparisons [16], [17]. All data are publicly available at https://vizhub.healthdata.org/gbd-results/. The GBD 2021 originates from various credible official sources, encompassing regional and national censuses, vital statistics, disease registries, and other authoritative databases. Data identification, extraction, and integration are rigorously performed through systematic assessments of published studies, alongside information from websites, reports and primary datasets from governments, international organizations and collaborators. Stringent quality control, evaluation, and calculation procedures are implemented to guarantee data accuracy. This study did not require ethical approval due to its use of publicly accessible data and adhered to the Guidelines for Accurate, and its methodological details are further elaborated in the latest GBD capstone publications [18], [19].

Data were obtained from the Global Burden of Disease Study 2021 (GBD 2021; data accessed on November 8, 2025). The estimates extracted pertained specifically to ischemic stroke (GBD cause)  attributable to high body-mass index (GBD risk). The measures included the number of deaths, disability-adjusted life years (DALYs), and their corresponding age-standardized rates (ASMR and ASDR). Data for all age groups, both sexes, and all locations  (global, regional, and national) as defined in GBD 2021 were included in the analysis.

For IS, the International Classification of Diseases (ICD-10) codes are I63.x, G46.3, and G46.4 [20]. BMI is a measure of body weight, calculated by an individual’s dividing weight (in kilograms) by the square of height (in meters) [21].It is commonly used for the primary diagnosis of obesity and overweight due to its simple calculation method. And it is also widely employed to assess nutritional status, body composition and physical development levels. According to the BMI classification, weight categories are defined as follows: underweight (BMI below 18.5), normal weight range (18.5 to 24.9), overweight (25.0 to 29.9), and obesity (greater than30.0). This study employed the disease burden estimates for ischemic stroke attributable to high body-mass index from the GBD 2021 study. The GBD’s risk attribution methodology was followed without modification [22].

The burden of IS-HBMI was measured by the number of deaths, disability-adjusted life years (DALYs), and their corresponding age-standardized rates (ASMR and ASDR). DALYs represent the sum of years lived with disability (YLDs) and years of life lost (YLLs). standardizedstandardizedstandardizedstandardizedstandardizedstandardizedstandardizedSpecifically, the Age-Standardized Mortality Rate (ASMR) were extracted to enable a more objective and accurate measurement of disease burden. Absolute cases and ASR were obtained directly from GBD 2021, which provided 95% uncertainty interval (UI), allowing parameter values to be estimated with relative reliability.

In examining disease burden in various countries/territories, we also employed the socio-demographic Index (SDI) to assess the level of socioeconomic development of a country or region. GBD 2021 divides countries/territories into five stages of socioeconomic development: high (SDI > 0.81), middle-high (SDI between 0.70 to 0.81), middle (SDI between 0.61 to 0.69), middle-low (SDI between 0.46 to 0.60), and low (SDI < 0.46). Based on this, differences in disease burden of IS-HBMI among countries/territories with differing degrees of economic development could be investigated, allowing for the establishment of more precise preventative measures adapted to the economic development context of each country/territory [23].

Joinpoint regression analysis was performed using the Joinpoint Regression Program (Version 4.9.1.0, National Cancer Institute) to identify significant inflection points in the temporal trends of age-standardized mortality rate (ASMR) and age-standardized DALY rate (ASDR) from 1990 to 2021. A log-linear model  was applied. The analysis allowed for a maximum of 7 joinpoints, with a permutation test  (using 4499 permutations and a significance level of 0.05) to determine the optimal number of joinpoints. Each segment was required to have a minimum length of 5 years. The models were weighted by the inverse of the variance  of the annual point estimates. The annual percent change (APC)  for each segment and its 95% confidence interval (CI) were calculated. The trend for the entire period was summarized by the estimated annual percentage change (EAPC) which is a weighted average of the segment-specific APCs [24]. To quantify the contributions of different drivers to the observed changes in the absolute number of IS-HBMI deaths between 1990 and 2021, we performed a demographic decomposition analysis. This method partitions the total change into three components: (1) population growth  (change in total population size), (2) population ageing  (change in the population age structure), and (3) epidemiological transition  (change in age-specific mortality rates). The analysis was conducted according to the standard demographic decomposition method [25].

The total change in deaths (ΔD) is expressed as the sum of the contributions from these three factors:

$$\mathrm{\Delta D}={\mathrm{\Delta D}}_{\text{population}}+{\mathrm{\Delta D}}_{\text{ageing}}+{\mathrm{\Delta D}}_{\text{epidemiological}}$$

The contribution of each component was calculated by constructing counterfactual scenarios. Specifically, we compared the observed death count in 2021 against hypothetical counts where two of the three factors were held constant at their 1990 levels while allowing the third to change. The results are presented as both the absolute number of deaths attributable to each driver and its percentage contribution to the net change observed from 1990 to 2021.

A prediction study was developed using Bayesian age-period-cohort (BAPC) model, in which Bayesian statistical techniques were applied to analyze correlations between age, period, and cohort in demographic data [26].This method is particularly suited for long-term epidemiological forecasting, as it separates the independent contributions of age (physiological risk), period (environmental and medical influences), and cohort (exposure factors specific to a birth cohort) to disease trends. The model parameterizes age (20 groups from 20-24 to 95+ years), period (7 intervals from 1990-1994 to 2020-2021), and derived birth cohort effects, with second-order random walk (RW2) priors assigned to each dimension to ensure smooth trends.  To ensure model identifiability, standard sum-to-zero constraints were applied to the period and cohort effects. Compared to pure time series models like ARIMA or machine learning approaches that may overfit short-term fluctuations, this model delivers more stable predictive outcomes. We selected the BAPC model over alternative approaches because the core objective is to forecast future disease burden based on dynamic shifts in population structure and risk exposure cohorts, rather than merely extrapolating historical temporal patterns [27]. BAPC model utilizes integrated nested Laplace approximations (INLA) for complete Bayesian inference, with the number of deaths modeled under a Poisson likelihood. Model fitting and validation were performed using INLA-specific diagnostics. The model’s predictive performance was assessed using the conditional predictive ordinate (CPO) and probability integral transform (PIT).  Diagnostic checks (CPO, PIT) indicated no major lack of fit, and the model with RW2 priors was selected based on the lowest DIC/WAIC. Previous studies have shown that the BAPC model has a relatively lower error rate compared to other prediction models, which can more accurately and comprehensively predict the trends in disease burden changes to ensure model robustness, we performed model comparison using the Deviance Information Criterion (DIC) and the Watanabe-Akaike Information Criterion (WAIC),  which indicated good model fit. Furthermore, we conducted sensitivity analyses on key modeling assumptions, including: (1) varying age group aggregations; and (2) applying different SDI classification boundaries. The main conclusions regarding socioeconomic gradients, sex disparities, and future trend directions remained consistent across these sensitivity analyses, underscoring the reliability of our findings [28]. For projections from 2022 to 2050, the fitted period and cohort effects were extrapolated. To estimate future death counts, the projected age- and sex-specific  mortality rates were then multiplied by the corresponding age- and sex-specific population denominators. These population denominators were derived from the United Nations World Population Prospects 2022 revision (medium-variant scenario). All statistical analysis and mapping were conducted by R software (version 4.3.2). All statistical tests were two sided, and P-values less than 0.05 were considered statistically significant.

To benchmark the efficiency of different regions, we constructed a best-performance frontier depicting the lowest theoretically achievable age-standardized mortality rate (ASMR) at each level of socioeconomic development (SDI). The frontier was empirically derived by fitting a quantile regression model (at the 5th percentile) to the relationship between ASMR and SDI across all region-year observations from 1990 to 2021. The resulting smoothed curve represents the minimum observed burden for any given SDI. We then quantified the deviation from this frontier  for each observation as the difference between its actual ASMR and the frontier value at its corresponding SDI, which estimates the excess burden attributable to factors beyond core socioeconomic development.

## 3 Results

### 3.1 Global disease burden of IS-HBMI in 2021

**Fig 1:**
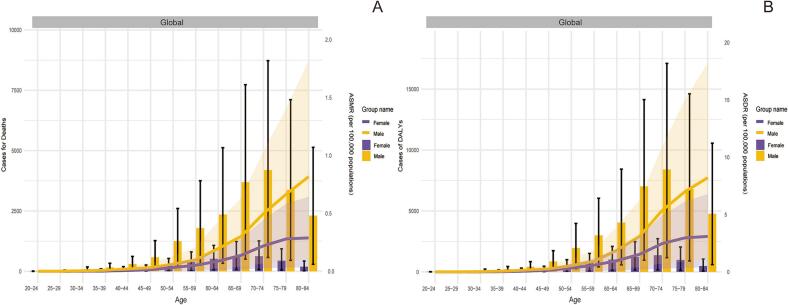
Cases and ASR of IS-HBMI by different sexes in 2021. Notes: (A) deaths, (B) DALYs. IS-HBMI, ischemic stroke attributable to high body-mass index; DALYs, the Disability-Adjusted Life Years; ASMR, age-standardized mortality rate; ASDR, age-standardized DALY rate.

**Fig 2:**
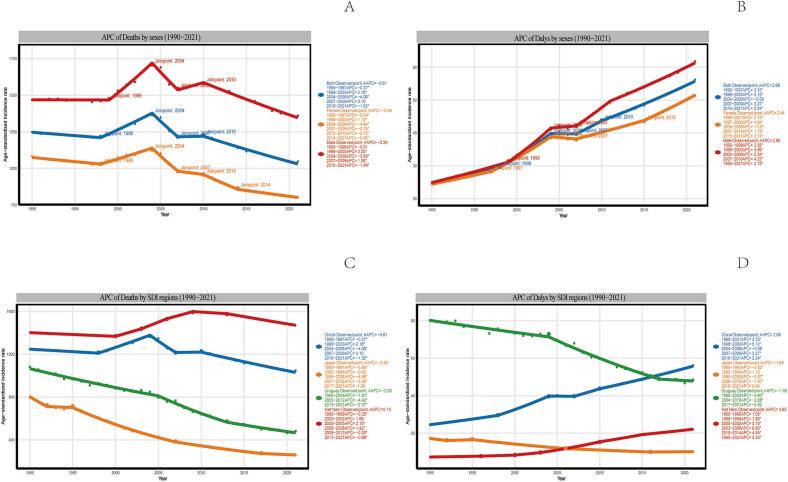
Trends of IS-HBMI from 1990 to 2021, calculated by Joinpoint regression.  (A, B) Trends by different sexes for deaths and DALYs, respectively. (C, D) Trends by SDI regions for deaths and DALYs, respectively. IS-HBMI, ischemic stroke attributable to high body-mass index; DALYs, disability-adjusted life years; EAPC, estimated annual percentage change.

**Fig 3:**
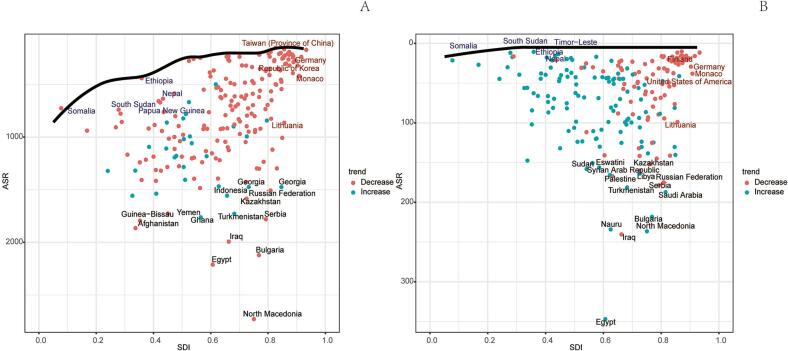
Frontier Analysis of Age-Standardized Mortality Rates for Ischemic Stroke in 2021 (Classified by Multiple Risk Factors and High Body Mass Index (HBMI)) Each data point represents a specific country or region, while the frontier line indicates the lowest achievable age-standardized mortality rate at a given socioeconomic development level. Countries/regions are plotted based on their Socioeconomic Development Index (SDI) and age-standardized mortality rate. Countries with lower Socio-Demographic Index (SDI) and minimal deviation from the frontier are highlighted in blue, while those with higher SDI and significant deviation relative to their development level are highlighted in red. Data point colors indicate trends in age-standardized mortality rates(ASMR) from 1990 to 2021. DALYs refer to disability-adjusted life years; HBMI denotes high body mass index.

**Fig 4:**
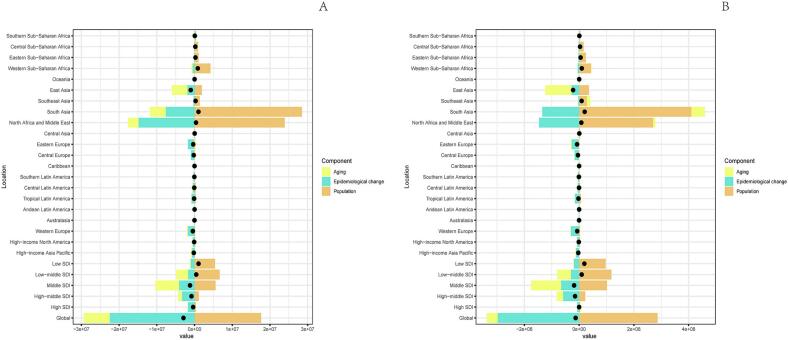
Decomposition analysis in deaths (A) and DALYs (B) of IS-HBMI from 1990 to 2021 by SDI regions and GBD regions. IS-HBMI, ischemic stroke attributable to high body-mass index; DALYs, the Disability-Adjusted Life Years; SDI, socio demographic index.

**Fig 5:**
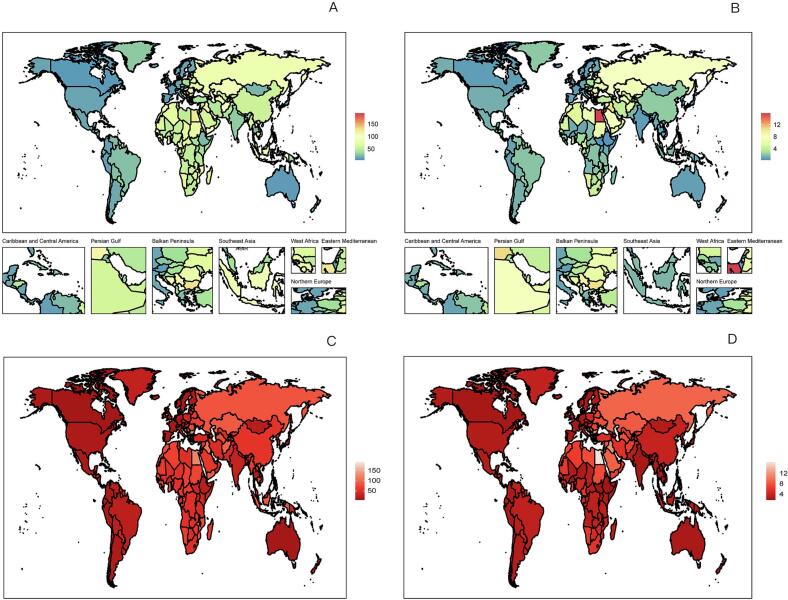
National Burden of Ischemic Stroke Attributable to High Body Mass Index, 2021  Age-standardized rates (A, B) and their average annual percentage change (C, D). Abbreviations:  EAPC, estimated annual percentage change; ASDR, age-standardized DALY rate; ASMR, age-standardized mortality rate; ASR, age-standardized rate; DALYs, disability-adjusted life years; HBMI, high body mass index; IS, ischemic stroke. Map lines delineate study areas and do not necessarily depict accepted national boundaries.

**Fig 6:**
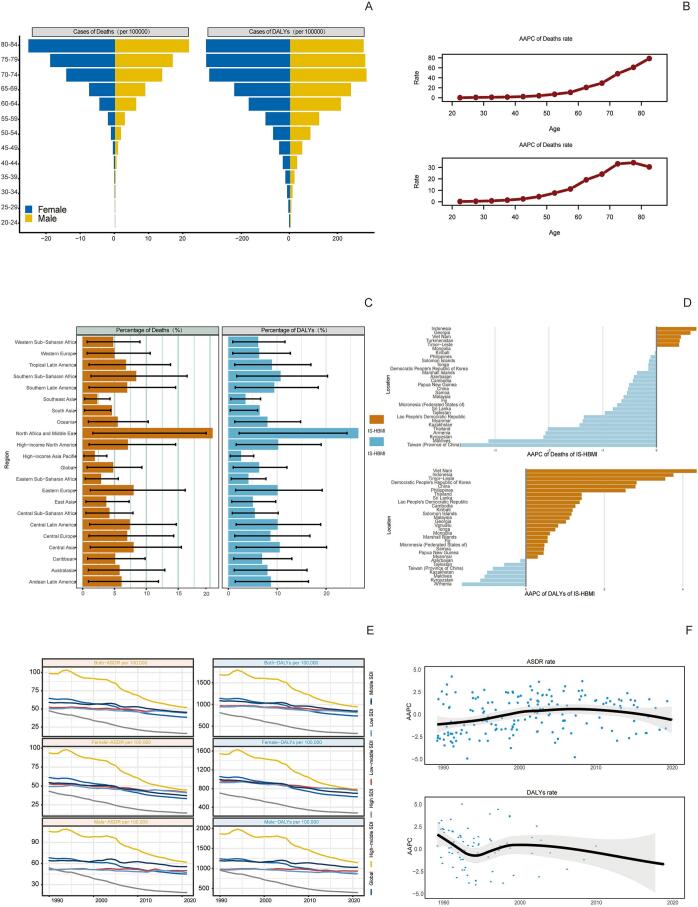
Burden of ischemic stroke attributable to high body-mass index (IS-HBMI): deaths, disability-adjusted life years (DALYs), and their Estimated Annual Percentage Change (EAPC), 1990–2021. Results are shown by age (A, for burden; B, for EAPC), by Socio-demographic Index (SDI) quintile and Global Burden of Disease (GBD) region (C), and by sex over time (E).

**Fig 7:**
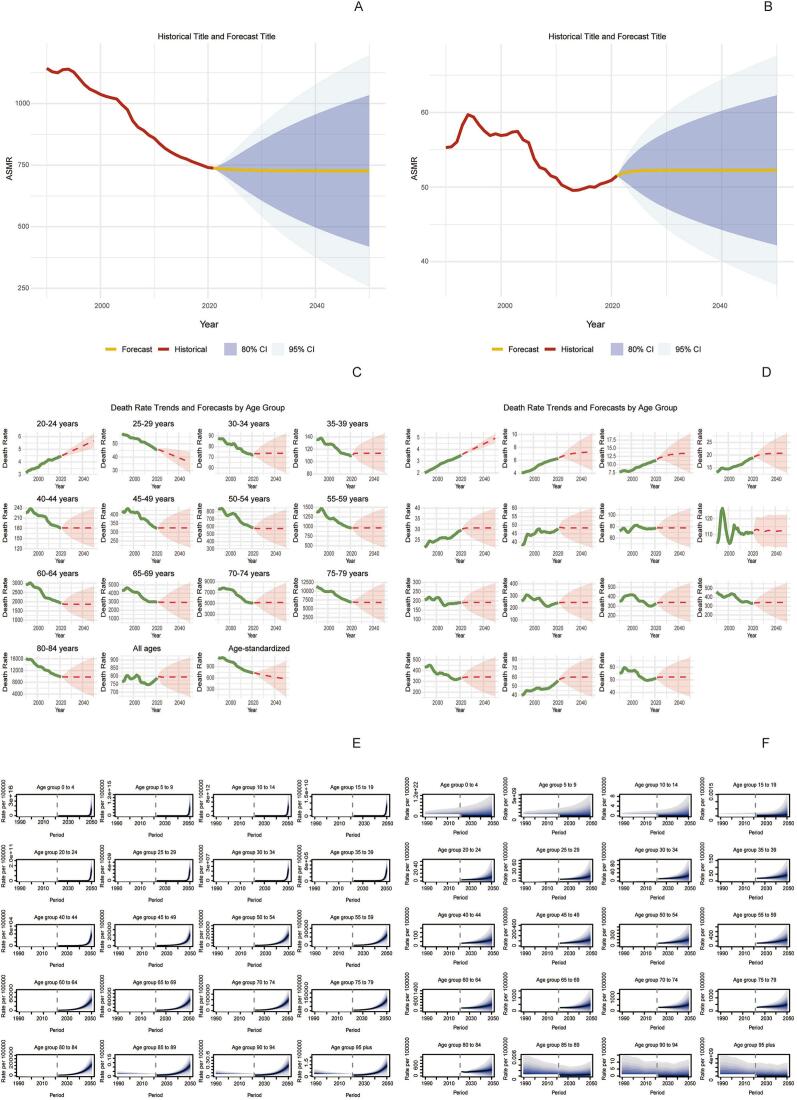
Historical Projected Number of Cases and Age-Standardized Rates of DALYs and Mortality for Ischemic Stroke Attributable to Multiple Risk Factors (A, C, E) and to High Body Mass Index (B, D, F) through 2050 Abbreviations:  ASDR, age-standardized DALY rate; ASMR, age-standardized mortality rate; DALYs, disability-adjusted life years; HBMI, high body mass index; IS, ischemic stroke.

In 2021, the global number of deaths from ischemic stroke (IS-HBMI) deaths attributable to high body mass index (IS-HBMI) was 172,391 (95% UI: [25,065-34,7913]), an increase of 95.7% compared with 1990 (88,075 [95% UI: 12,636-176,367]). The age-standardizedstandardizedstandardizedstandardizedstandardizedstandardizedstandardized mortality rate (ASMR) declined from 2.57  (95% UI: 0.37-5.15) to 2.06 per 100,000  (95% UI: 0.30-4.17), with an estimated annual percentage change (EAPC) of -1.10%  (95% CI: -1.25 to -0.96). standardizedstandardizedstandardizedstandardizedstandardizedstandardizedstandardized Similarly, disability-adjusted life years (DALYs) increased to 44,391.86 thousand (95% UI: 6,490.3-86,474.85) in 2021, while the age-standardized DALY rate (ASDR) showed a modest decline (EAPC: -0.58%, 95% CI: -0.71 to -0.46). Significant socioeconomic disparities were observed: for example, the EAPC for ASMR was -2.60%  in high-SDI regions but +1.54%  in low-SDI regions (Table 1). (See Fig 1, Fig 2, Fig 3, Fig 4, Fig 5, Fig 6, Fig 7.)

**Table 1:** Death cases and age-standardized mortality rate (ASMR) of ischemic stroke attributable to high body-mass index, by sex, Socio-demographic Index (SDI) quintile, and Global Burden of Disease (GBD) region, 1990 and 2021, with estimated annual percentage change (EAPC), 1990–2021. Note: The unit for the number of DALYs is “thousands.” EAPC indicates estimated annual percentage Change; ASR, age-standardized rate; DALYs, disability-adjusted life-years; GBD, Global Burden of Disease; HBMI, high body mass index; SDI, sociodemographic index; and UI, uncertainty interval. Note: The unit for the number of DALYs is thousands.

All risks death and ASMR						
	1990		2021		1990-2021	
	Death cases	ASMR per 100,000	Death cases	ASMR per 100,000	EAPC of ASMR	
	No. *102 (95% UI)	No. (95% UI)	No. *102 (95% UI)	No.(95% UI)	No. (95% CI)	
Overall	20612.18 [18674.12-22466.01]	64.58 [57.79-70.43]	31459.29 [27769.21-34852.96]	38.57 [33.81-42.77]	-1.87 [-1.96 to -1.77]	-1.87
Sex						
Female	11532.73 [10278.18-12617.57]	61.29 [54.05-67.15]	15677.87 [13271.28-17757.6]	33.35 [28.26-37.75]	-2.21 [-2.33 to -2.1]	-2.21
Male	9079.46 [8116.43-10071.06]	68.11 [60.68-75.24]	15781.42 [13980.79-17657.04]	45.01 [39.86-50.17]	-1.48 [-1.56 to -1.4]	-1.48
Socio-demographic index						
High SDI	5250.25 [4627.39-5702.32]	47.32 [41.55-51.57]	4248.14 [3494.06-4838.54]	16.36 [13.65-18.46]	-3.74 [-3.89 to -3.58]	-3.74
High-middle SDI	7914.17 [7133.94-8519.13]	98.93 [88.57-107.1]	10043.65 [8752.11-11309.47]	51.96 [45.19-58.59]	-2.45 [-2.66 to -2.24]	-2.45
Middle SDI	4334.39 [3855.3-4939.99]	58.93 [51.61-66.93]	10345.59 [9040.39-11610.37]	45.26 [39.24-50.82]	-0.88 [-0.99 to -0.78]	-0.88
Low-middle SDI	2307.54 [1993.28-2686.11]	52.35 [44.97-60.94]	5225.96 [4613.37-5928.04]	45.39 [39.79-51.12]	-0.47 [-0.52 to -0.43]	-0.47
Low SDI	770.33 [629.41-991.49]	50.31 [41.36-64.25]	1560.81 [1317-1961.11]	43.91 [37-54.05]	-0.45 [-0.51 to -0.39]	-0.45
Region						
Andean Latin America	45.24 [39.42-50.97]	26.13 [22.84-29.57]	81.22 [66.76-98.95]	14.69 [12.06-17.89]	-2.08 [-2.28 to -1.87]	-2.08
Australasia	84.59 [72.55-93.97]	38.87 [33.14-43.41]	76.01 [60.43-88.89]	11.46 [9.17-13.36]	-4.18 [-4.31 to -4.06]	-4.18
Caribbean	100.13 [89.15-110.55]	43.97 [38.67-48.53]	166.05 [142.78-192.83]	30.35 [26.1-35.3]	-1.14 [-1.22 to -1.06]	-1.14
Central Asia	290.02 [259.24-316.77]	71.44 [63.56-78.27]	399.85 [354.62-443.7]	62.71 [55.48-69.98]	-0.84 [-1.09 to -0.58]	-0.84
Central Europe	1647.98 [1509.45-1754.91]	127.32 [115.64-135.99]	1405.94 [1253.08-1555.17]	57.61 [51.34-63.69]	-2.89 [-3.04 to -2.73]	-2.89
Central Latin America	207.99 [187.39-223.63]	32.02 [28.74-34.67]	363.9 [311.08-417.33]	15.77 [13.45-18.1]	-2.43 [-2.6 to -2.26]	-2.43
Central Sub-Saharan Africa	76.57 [56.86-97.99]	58.22 [44.07-73.75]	168.39 [125.13-228.85]	52.72 [38.63-71.86]	-0.48 [-0.54 to -0.42]	-0.48
East Asia	4010.06 [3393.1-4750.53]	66.73 [56.8-79.17]	10635.41 [8810.69-12542.65]	55.46 [45.82-65.04]	-0.56 [-0.83 to -0.3]	-0.56
Eastern Europe	3589.04 [3239-3837.16]	147.55 [131.77-158.61]	2847.09 [2486.83-3162.52]	78.71 [68.76-87.44]	-2.84 [-3.31 to -2.36]	-2.84
Eastern Sub-Saharan Africa	215.57 [172.39-273.23]	44.46 [35.97-55.38]	458.24 [378.07-546.9]	40.55 [33.13-48.51]	-0.38 [-0.42 to -0.33]	-0.38
High-income Asia Pacific	926.94 [811.65-1008.04]	54.68 [47.09-59.94]	920.35 [697.75-1081.99]	13.06 [10.22-15.08]	-4.97 [-5.15 to -4.79]	-4.97
High-income North America	948.21 [802.17-1053.63]	25.43 [21.45-28.29]	1042.73 [829.32-1198.54]	13.86 [11.11-15.86]	-2.55 [-2.89 to -2.22]	-2.55
North Africa and Middle East	1136.13 [976.82-1314.94]	92.22 [78.16-106.69]	2246.78 [1940.39-2553.48]	64.66 [55.23-73.23]	-1.14 [-1.17 to -1.1]	-1.14
Oceania	6.91 [5.28-9.14]	41.2 [32.62-53]	15.88 [12.57-20.56]	34.75 [27.86-44.37]	-0.67 [-0.74 to -0.6]	-0.67
South Asia	1562.77 [1265.43-1990.13]	38.95 [31.75-48.98]	3971.52 [3362.91-4937.59]	33.9 [28.91-41.4]	-0.59 [-0.67 to -0.51]	-0.59
Southeast Asia	1190.72 [1035.76-1349.14]	65.44 [56.26-74.88]	3039.16 [2579.74-3511.66]	60.1 [51.59-69.06]	-0.2 [-0.36 to -0.03]	-0.2
Southern Latin America	198.11 [175.76-218.2]	48.68 [42.68-53.78]	169.34 [145.77-188.71]	18.23 [15.69-20.3]	-2.8 [-2.95 to -2.65]	-2.8
Southern Sub-Saharan Africa	87.55 [70.83-100.32]	42.04 [33.89-48.37]	221.07 [194.96-245.01]	52.8 [45.91-58.78]	0.89 [0.38 to 1.4]	0.89
Tropical Latin America	484.64 [432.59-521.34]	69.5 [61.33-75.63]	605.53 [515.31-673.78]	24.97 [21.23-27.85]	-3.12 [-3.24 to -3]	-3.12
Western Europe	3372.26 [2949.84-3663.86]	55.5 [48.41-60.51]	1789.21 [1446.53-2054.08]	13.97 [11.43-15.93]	-4.65 [-4.8 to -4.49]	-4.65
Western Sub-Saharan Africa	430.77 [336.99-563.32]	66.94 [52.45-86.68]	835.63 [696.4-998.57]	61.34 [51.53-72.3]	-0.25 [-0.36 to -0.14]	-0.25
China	3879.6 [3272.65-4616.82]	67.32 [57.13-80.16]	10410.55 [8579.19-12307.91]	56.57 [46.69-66.48]	-0.52 [-0.8 to -0.25]	-0.52
Indonesia	429.15 [349.26-512.67]	65.61 [52.46-79.07]	1332.07 [1002-1667.77]	87.55 [68.18-108.39]	1.02 [0.83 to 1.2]	1.02
Democratic People's Republic of Korea	73.2 [54.53-95.87]	62.38 [46.32-81.51]	172.21 [135.23-224.91]	58.27 [45.78-75.88]	-0.26 [-0.45 to -0.07]	-0.26
Cambodia	20.17 [16.3-25.05]	68.45 [55.72-84.12]	53.65 [41.99-65.35]	66.83 [52.82-80.39]	-0.17 [-0.28 to -0.06]	-0.17
Taiwan (Province of China)	57.26 [51.55-61.85]	49.67 [44.25-54.18]	52.66 [43.87-60.37]	11.8 [9.87-13.49]	-4.61 [-4.87 to -4.34]	-4.61
Malaysia	36.42 [31.12-42.14]	46.77 [39.95-54.21]	85.45 [74.84-97.8]	37.95 [32.61-43.86]	-0.41 [-0.59 to -0.22]	-0.41
Timor-Leste	0.92 [0.74-1.12]	56.75 [46.26-68.84]	4.04 [2.9-5.31]	62.13 [45.49-81.05]	0.47 [0.32 to 0.63]	0.47
Myanmar	152.48 [115.22-190.71]	91.53 [72.16-111.67]	247.7 [197.62-311.67]	66.27 [52.95-83.66]	-1.22 [-1.31 to -1.14]	-1.22
Sri Lanka	65.18 [57.85-72.51]	92.8 [82.77-103.1]	131.28 [91.61-169.76]	60.5 [42.57-77.48]	-0.64 [-0.96 to -0.32]	-0.64
Lao People's Democratic Republic	14.08 [11.04-17.7]	97.45 [77.78-122.1]	22.51 [17.38-28.09]	70.1 [55.07-86.17]	-1.21 [-1.29 to -1.13]	-1.21
Maldives	0.39 [0.33-0.46]	71.6 [59.25-84.51]	0.86 [0.68-1.02]	33.97 [26.89-40.4]	-2.75 [-2.84 to -2.65]	-2.75
Thailand	103.46 [83.89-124.68]	38.97 [31.41-46.86]	244.75 [187.74-312.58]	22.58 [17.26-28.82]	-2.35 [-2.62 to -2.09]	-2.35
Kiribati	0.11 [0.09-0.13]	45.01 [34.02-54.78]	0.21 [0.17-0.26]	48 [39.03-58.9]	0.16 [0.09 to 0.23]	0.16
Philippines	91.14 [78.59-101.19]	49.4 [43.62-54.69]	244.76 [208.17-283.89]	39.31 [33.72-45.39]	-0.52 [-0.64 to -0.41]	-0.52
Micronesia (Federated States of)	0.21 [0.16-0.27]	56.54 [42.85-72.96]	0.21 [0.16-0.28]	45.78 [34.97-59.95]	-0.78 [-0.84 to -0.71]	-0.78
Viet Nam	270.28 [215.92-340.26]	80.95 [65.19-102.12]	662.39 [526.62-791.91]	84.92 [68.21-101.14]	0.43 [0.27 to 0.58]	0.43
Marshall Islands	0.06 [0.04-0.07]	52.75 [39.94-68.1]	0.09 [0.06-0.12]	47.43 [36.37-62.66]	-0.4 [-0.47 to -0.34]	-0.4
Fiji	0.86 [0.7-1.04]	39.01 [31.47-46.9]	1.77 [1.39-2.26]	37.29 [29.48-46.1]	-0.56 [-0.8 to -0.31]	-0.56
Papua New Guinea	3.9 [2.58-5.66]	40.09 [27.39-56.86]	10.15 [7.25-14.27]	35.64 [26.02-49.1]	-0.45 [-0.56 to -0.34]	-0.45
Solomon Islands	0.37 [0.27-0.5]	49.66 [37.6-66.38]	1 [0.72-1.4]	48.17 [35.7-66.07]	-0.13 [-0.24 to -0.02]	-0.13
Samoa	0.28 [0.22-0.34]	45.46 [36.77-55.75]	0.42 [0.34-0.51]	37.87 [31.35-46.17]	-0.67 [-0.76 to -0.59]	-0.67
Tonga	0.11 [0.09-0.14]	27.33 [21.65-33.23]	0.19 [0.15-0.24]	26.08 [19.77-32.41]	0.02 [-0.12 to 0.15]	0.02
Vanuatu	0.22 [0.17-0.28]	60.5 [46.8-75.38]	0.53 [0.39-0.66]	49.47 [37.09-61.36]	-0.8 [-0.86 to -0.74]	-0.8
Azerbaijan	15.92 [12.26-19.59]	39.3 [30.24-48.52]	25.56 [19.62-32.95]	34.62 [26.53-43.98]	0 [-0.28 to 0.28]	0
Armenia	14.24 [12.53-15.66]	64.05 [56.16-70.63]	19.3 [16.79-21.79]	44.67 [38.83-50.35]	-2.12 [-2.49 to -1.75]	-2.12
Kazakhstan	112.51 [97.88-125.55]	105.73 [92.11-117.49]	118.79 [102.59-135.91]	90.46 [78.55-102.64]	-0.98 [-1.44 to -0.52]	-0.98
Georgia	37.18 [30.02-43.39]	67.03 [54.45-78.04]	55.91 [49.01-63.59]	84.16 [74.23-95.32]	0.78 [0.08 to 1.47]	0.78
Kyrgyzstan	25.65 [22.57-28.56]	99.6 [87.08-111.13]	21.04 [17.24-24.65]	53.2 [43.41-62.44]	-2.45 [-2.81 to -2.08]	-2.45
Mongolia	1.79 [1.36-2.33]	21.22 [15.91-27.57]	3.4 [2.43-4.42]	21.18 [15.24-27.85]	-0.11 [-0.54 to 0.32]	-0.11
Tajikistan	17.79 [14.26-21.35]	74.74 [59.53-88.92]	24.81 [19.8-30.42]	64.28 [51.01-77.96]	-0.78 [-1.2 to -0.36]	-0.78
Turkmenistan	10.33 [8.4-12.07]	64.73 [53.03-75.66]	26.08 [19.72-33.3]	79.31 [61.5-100.62]	0.33 [-0.17 to 0.83]	0.33
Uzbekistan	54.6 [46.93-60.83]	51.71 [44.3-57.51]	104.97 [89.15-121.65]	53.35 [45.27-62.11]	-0.57 [-0.97 to -0.17]	-0.57
Bosnia and Herzegovina	35.66 [31.19-40.62]	115.07 [100.6-130.66]	52.67 [41.98-63.08]	81.14 [64.47-97.45]	-1.44 [-1.62 to -1.26]	-1.44
Albania	7.76 [6.38-9.34]	49.61 [40.74-59.65]	15.99 [11.8-20.95]	39.42 [29.39-51.66]	-0.32 [-0.6 to -0.04]	-0.32
Croatia	63.39 [57.06-67.99]	127.19 [114.27-136.45]	43.97 [37.57-50.14]	42.34 [36.21-48.32]	-3.74 [-3.89 to -3.59]	-3.74
Bulgaria	140.11 [128.41-150.15]	162.04 [146.96-173.64]	190.86 [165.29-218.29]	128.78 [111.51-146.58]	-0.49 [-0.68 to -0.3]	-0.49
Hungary	166.81 [152-177.5]	123.28 [112.23-131.47]	83.8 [70.31-96.08]	38.16 [32.04-43.74]	-4.14 [-4.36 to -3.93]	-4.14
Czechia	188.16 [170.03-204.76]	140.82 [127.41-153.59]	64.06 [54.96-73.08]	26.77 [22.98-30.52]	-5.77 [-6.22 to -5.32]	-5.77
North Macedonia	27.53 [24.43-30.43]	180.45 [159.79-199.69]	43.98 [35.96-52.78]	192.91 [162.96-226.39]	-0.14 [-0.69 to 0.41]	-0.14
Montenegro	1.96 [1.57-2.39]	35.66 [28.4-43.2]	4.62 [3.62-5.73]	56.68 [44.55-70]	1.86 [1.59 to 2.14]	1.86
Romania	309.69 [278.44-336.26]	143.73 [129.6-156.11]	336.65 [292.55-380.48]	78.97 [68.65-89.26]	-2.4 [-2.65 to -2.14]	-2.4
Poland	445.83 [408.65-476.86]	112.5 [101.76-120.71]	294.73 [252.86-332.76]	37.07 [31.91-41.81]	-3.99 [-4.12 to -3.85]	-3.99
Slovakia	50.67 [44.42-57.47]	89.08 [78.04-100.82]	41.28 [34.17-49.36]	42.86 [35.5-51.33]	-2.44 [-2.52 to -2.36]	-2.44
Serbia	165.62 [145.95-185.09]	215.42 [189.62-239.99]	200.5 [167.79-235.16]	113.64 [95.23-133.65]	-2.72 [-3 to -2.43]	-2.72
Estonia	23.76 [21.19-25.85]	119.86 [106.4-130.6]	7.61 [6.32-8.77]	22.92 [19.21-26.48]	-6.86 [-7.49 to -6.22]	-6.86
Slovenia	18.44 [16.63-19.89]	75.75 [68.08-82]	12.37 [10.19-14.38]	21.82 [18.06-25.28]	-3.99 [-4.22 to -3.77]	-3.99
Belarus	114.47 [100.82-124.57]	92.36 [80.94-100.75]	107.46 [88.91-128.31]	64.88 [53.65-77.28]	-1.82 [-2.22 to -1.41]	-1.82
Latvia	46.16 [41.56-49.99]	131.85 [118.21-142.8]	37.33 [31.68-42.65]	76.62 [65.45-87.16]	-2.24 [-2.52 to -1.96]	-2.24
Republic of Moldova	24.82 [21.99-27.5]	77.72 [68.52-85.55]	28.05 [24.5-31.93]	46.05 [40.17-52.48]	-1.48 [-1.92 to -1.04]	-1.48
Ukraine	880.14 [795.83-945.5]	137.18 [122.81-148.47]	560.25 [439.03-696.75]	69.7 [54.36-86.42]	-2.94 [-3.28 to -2.6]	-2.94
Lithuania	30.48 [27.18-33.1]	68.06 [60.76-74.03]	32.77 [27.78-37.3]	47.02 [40.1-53.49]	-1.47 [-1.79 to -1.14]	-1.47
Russian Federation	2469.21 [2224.66-2639.88]	161.99 [144.17-174.33]	2073.61 [1810.89-2307.82]	85.45 [74.68-95.09]	-2.92 [-3.48 to -2.37]	-2.92
Japan	730.84 [634.74-798.2]	49.24 [42.19-54.3]	725.1 [541.3-863.2]	11.8 [9.19-13.73]	-4.86 [-5.04 to -4.68]	-4.86
Australia	69.78 [59.55-77.86]	38.78 [33.04-43.5]	60.89 [47.99-71.51]	10.74 [8.53-12.57]	-4.39 [-4.52 to -4.25]	-4.39
Singapore	8.35 [7.48-9.09]	48.55 [43.04-53.25]	4.42 [3.54-5.16]	5.43 [4.34-6.35]	-6.88 [-7.34 to -6.41]	-6.88
Brunei Darussalam	0.46 [0.38-0.54]	64.04 [53.84-75.32]	0.57 [0.47-0.68]	30.91 [25.02-36.84]	-1.99 [-2.32 to -1.67]	-1.99
Republic of Korea	187.29 [162.67-211.61]	100.26 [85.07-112.75]	190.25 [150.09-228.87]	20.94 [16.47-25.25]	-5.82 [-6.11 to -5.52]	-5.82
New Zealand	14.81 [12.74-16.32]	39.3 [33.44-43.46]	15.12 [12.3-17.58]	15.61 [12.77-18.13]	-3.21 [-3.37 to -3.06]	-3.21
Cyprus	4.8 [3.98-5.78]	107.61 [88.15-128.97]	4.33 [3.46-5.2]	27.94 [22.4-33.75]	-4.63 [-4.99 to -4.28]	-4.63
Austria	73.88 [63.61-81.11]	58.41 [50.1-64.3]	26.62 [21.59-30.75]	11.12 [9.13-12.74]	-5.75 [-6.13 to -5.38]	-5.75
Finland	38.16 [33.27-42.02]	52.33 [45.28-57.75]	26.87 [21.55-31.07]	15.89 [13-18.27]	-3.83 [-3.99 to -3.67]	-3.83
Andorra	0.11 [0.08-0.15]	25.04 [18.19-33.02]	0.22 [0.15-0.28]	11.69 [8.23-15.32]	-2.23 [-2.46 to -2]	-2.23
Belgium	79.13 [68.83-87.08]	49.67 [42.85-54.84]	40.5 [31.87-46.66]	12.82 [10.34-14.65]	-4.2 [-4.33 to -4.07]	-4.2
Denmark	39.52 [34.86-43.29]	43.71 [38.59-47.88]	22.98 [18.77-26.22]	16.05 [13.17-18.27]	-3.61 [-3.83 to -3.4]	-3.61
France	349.6 [299.36-387.97]	38.12 [32.59-42.32]	235.91 [187.66-274.52]	11.1 [9.01-12.83]	-3.93 [-4.07 to -3.78]	-3.93
Germany	832.12 [719.55-908.07]	60.91 [52.6-66.58]	383.1 [312.75-438.07]	15.14 [12.45-17.14]	-4.55 [-4.83 to -4.27]	-4.55
Iceland	1.05 [0.88-1.18]	33.34 [28.28-37.49]	0.82 [0.63-0.97]	11.1 [8.66-13.04]	-3.66 [-3.87 to -3.44]	-3.66
Luxembourg	4.28 [3.77-4.63]	81.02 [71.33-88.11]	1.71 [1.42-1.96]	13.35 [11.11-15.25]	-5.61 [-5.74 to -5.48]	-5.61
Netherlands	84.54 [72.81-93.67]	40.88 [35.06-45.32]	72.05 [59.02-83.22]	17.36 [14.26-20.01]	-3.39 [-3.73 to -3.04]	-3.39
Ireland	21.13 [18.83-23.02]	54.76 [48.31-59.76]	10.66 [8.5-12.31]	12.13 [9.69-14]	-4.83 [-5.05 to -4.6]	-4.83
Greece	127.63 [112.34-138.76]	90.82 [79.02-98.81]	88.7 [72-101.85]	24.22 [19.88-27.71]	-5.05 [-5.46 to -4.65]	-5.05
Israel	13.55 [12.02-15.05]	30.86 [27.09-34.25]	13.52 [10.92-15.48]	9.23 [7.54-10.53]	-4.37 [-4.57 to -4.18]	-4.37
Italy	515.55 [448.14-563.85]	58.97 [50.58-64.83]	336.49 [257.54-395]	15.57 [12.14-18.14]	-4.56 [-4.79 to -4.33]	-4.56
Malta	2.04 [1.79-2.24]	52.49 [45.8-57.9]	1.49 [1.18-1.75]	12.63 [10.08-14.77]	-4.65 [-4.93 to -4.38]	-4.65
Norway	38.83 [33.77-42.63]	48.87 [42.53-53.68]	15.36 [12.41-17.74]	12.01 [9.84-13.79]	-4.76 [-4.94 to -4.57]	-4.76
Spain	324.1 [283.95-355.01]	61.53 [53.31-67.68]	161.34 [129.13-189.01]	11.32 [9.24-13.14]	-5.44 [-5.65 to -5.22]	-5.44
Portugal	167.82 [148.61-184.25]	138.31 [120.68-152.8]	77.36 [63.19-90.04]	22.81 [18.82-26.43]	-6.41 [-6.66 to -6.16]	-6.41
Switzerland	44.02 [37.72-49.19]	37.05 [31.73-41.39]	24.39 [18.98-28.6]	9.59 [7.56-11.14]	-4.24 [-4.37 to -4.1]	-4.24
Sweden	71.36 [61.28-79.09]	40.69 [34.92-45.09]	38.62 [30.51-45.06]	13.22 [10.54-15.42]	-3.76 [-4.04 to -3.49]	-3.76
Chile	43.8 [39.77-47.09]	51.67 [46.75-55.78]	52.9 [45.61-58.79]	19.88 [17.13-22.05]	-2.55 [-2.77 to -2.33]	-2.55
United Kingdom	535.52 [479.17-577.38]	54.88 [48.73-59.33]	204.11 [166.51-233.45]	12.43 [10.24-14.16]	-5.11 [-5.36 to -4.85]	-5.11
Argentina	130.01 [113.69-145.22]	45.73 [39.68-50.97]	97.13 [83.77-108.79]	16.38 [14.14-18.34]	-2.89 [-3.1 to -2.67]	-2.89
United States of America	859.73 [726.89-955.48]	25.19 [21.25-28.02]	956.47 [760.06-1098.97]	14.38 [11.53-16.45]	-2.42 [-2.77 to -2.07]	-2.42
Uruguay	24.29 [21.23-26.79]	63.34 [55.06-69.87]	19.3 [16.26-21.68]	27.7 [23.42-31]	-2.83 [-3.04 to -2.61]	-2.83
Bahamas	0.43 [0.36-0.5]	33.47 [28.24-38.56]	0.77 [0.63-0.94]	23.66 [19.14-28.9]	-1.15 [-1.32 to -0.98]	-1.15
Belize	0.22 [0.19-0.25]	24.49 [21.29-27.24]	0.53 [0.44-0.6]	21.68 [18.07-24.82]	-0.78 [-1.36 to -0.21]	-0.78
Canada	88.3 [75.84-98.62]	28.14 [24.05-31.51]	86.12 [69.74-100.01]	9.8 [7.97-11.35]	-3.83 [-4.04 to -3.61]	-3.83
Antigua and Barbuda	0.28 [0.23-0.31]	46.27 [39.25-51.95]	0.26 [0.23-0.29]	30.37 [26.18-33.82]	-1.52 [-1.78 to -1.27]	-1.52
Dominica	0.31 [0.27-0.36]	55.61 [47.39-64.13]	0.33 [0.27-0.38]	44.24 [37.01-52.03]	-0.76 [-0.87 to -0.66]	-0.76
Barbados	1.86 [1.61-2.06]	59.39 [50.89-65.77]	1.97 [1.57-2.35]	37.75 [30.02-45.15]	-1.65 [-1.87 to -1.44]	-1.65
Grenada	0.64 [0.55-0.72]	76.82 [65.6-86.35]	0.38 [0.32-0.42]	42.97 [36.22-48.28]	-1.81 [-2.04 to -1.58]	-1.81
Cuba	33.2 [28.72-36.9]	35.4 [30.37-39.3]	59.61 [49.98-68.78]	27.91 [23.42-32.16]	-0.81 [-0.93 to -0.69]	-0.81
Haiti	20.83 [15.91-26.02]	98.85 [76.8-121.2]	35.76 [25.28-50.61]	76.3 [55.35-106.31]	-0.72 [-0.78 to -0.66]	-0.72
Dominican Republic	9.7 [8.04-11.31]	34.84 [28.78-40.6]	26.84 [20.9-34.57]	28.25 [21.98-36.33]	-0.14 [-0.42 to 0.14]	-0.14
Ecuador	13.02 [11.32-14.52]	29.49 [25.61-32.83]	22.29 [17.75-27.67]	15.43 [12.41-18.96]	-1.92 [-2.21 to -1.62]	-1.92
Guyana	3.03 [2.68-3.37]	97.47 [85.98-108.09]	2.95 [2.36-3.68]	60.47 [48.77-74.59]	-1.02 [-1.26 to -0.78]	-1.02
Saint Lucia	0.7 [0.62-0.77]	102.67 [91.31-112.88]	0.97 [0.79-1.17]	43.41 [35.03-51.8]	-3.56 [-4.03 to -3.09]	-3.56
Jamaica	10.45 [9.06-11.62]	55.17 [47.88-61.41]	12.88 [9.94-15.89]	38.01 [29.52-46.97]	-1.03 [-1.44 to -0.62]	-1.03
Suriname	1.05 [0.89-1.18]	48.12 [40.56-54.33]	1.98 [1.51-2.5]	34.71 [26.11-43.95]	-1.01 [-1.28 to -0.73]	-1.01
Saint Vincent and the Grenadines	0.4 [0.35-0.45]	63.81 [54.54-71.39]	0.49 [0.42-0.56]	39.92 [33.92-45.61]	-1.34 [-1.61 to -1.07]	-1.34
Trinidad and Tobago	4.79 [4.23-5.25]	71.46 [62.72-79.08]	6.56 [5-8.16]	36.12 [27.62-44.96]	-2.42 [-2.67 to -2.18]	-2.42
Bolivia (Plurinational State of)	10.13 [7.33-13.37]	42.01 [30.9-54.24]	17.83 [12.46-24.75]	25.3 [17.94-34.3]	-1.59 [-1.72 to -1.46]	-1.59
Peru	22.08 [18.24-26.08]	21.31 [17.56-25.23]	41.09 [31.66-52.3]	12.42 [9.56-15.82]	-2.29 [-2.74 to -1.84]	-2.29
Colombia	46.88 [42.08-50.84]	33.28 [29.63-36.25]	69.95 [57.13-82.56]	12.46 [10.18-14.72]	-3.65 [-3.89 to -3.4]	-3.65
El Salvador	5.94 [5.06-6.74]	21.59 [18.33-24.55]	9.05 [7.1-11.18]	13.23 [10.42-16.31]	-1.78 [-2.08 to -1.48]	-1.78
Costa Rica	3.67 [3.19-4.05]	23.34 [20.24-25.78]	7.68 [6.41-8.83]	13.64 [11.41-15.61]	-2.26 [-2.68 to -1.84]	-2.26
Honduras	5.78 [4.48-7.21]	36.56 [28.36-45.32]	22.93 [17.39-29.21]	47.85 [36.45-60.19]	1.02 [0.77 to 1.28]	1.02
Nicaragua	3.22 [2.77-3.69]	26.37 [22.73-30.32]	6.02 [4.93-7.52]	14.95 [12.2-18.61]	-1.67 [-1.87 to -1.46]	-1.67
Guatemala	5.63 [4.9-6.29]	26.43 [23.09-29.47]	13.86 [11.66-16.14]	15.56 [13.08-18.08]	-2.22 [-2.57 to -1.87]	-2.22
Venezuela (Bolivarian Republic of)	22.22 [19.31-24.72]	28.52 [24.62-31.77]	61.34 [45.86-79]	23.25 [17.47-29.71]	-0.87 [-1.17 to -0.58]	-0.87
Mexico	110.51 [99.94-118.46]	35.12 [31.59-37.81]	164.16 [140.57-186.46]	14.77 [12.63-16.79]	-2.79 [-2.94 to -2.64]	-2.79
Panama	4.14 [3.58-4.63]	31.5 [27.12-35.3]	8.9 [6.79-10.73]	19.17 [14.67-23.08]	-1.77 [-1.99 to -1.56]	-1.77
Brazil	474.79 [424.24-510.29]	70.01 [61.67-76.19]	588.37 [501.16-654.37]	24.79 [21.1-27.63]	-3.17 [-3.28 to -3.05]	-3.17
Algeria	68.43 [53.23-85.05]	99.66 [78.4-122.14]	167.06 [128.19-209.73]	70.89 [54.54-87.62]	-0.87 [-0.93 to -0.81]	-0.87
Egypt	255.76 [189.67-351.42]	153.45 [117.12-205.01]	495.38 [391.71-621.68]	124.48 [100.79-151.5]	-0.14 [-0.35 to 0.06]	-0.14
Paraguay	9.85 [8.33-11.34]	51.4 [43.19-59.21]	17.16 [13.34-21.47]	33.35 [25.97-41.69]	-1.12 [-1.33 to -0.9]	-1.12
Bahrain	0.75 [0.66-0.84]	87.33 [75.81-98.03]	1.94 [1.59-2.29]	51.58 [43.11-60.02]	-2.14 [-2.63 to -1.64]	-2.14
Iraq	81.2 [67.16-95.64]	113.81 [93.8-133.84]	188.6 [150.03-226.03]	112.77 [90.24-133.61]	-0.61 [-0.83 to -0.39]	-0.61
Iran (Islamic Republic of)	144.31 [126.38-161.78]	84.01 [72.37-94.9]	292.94 [254.49-324.32]	45.24 [38.94-50.28]	-2.14 [-2.26 to -2.01]	-2.14
Kuwait	1.33 [1.17-1.47]	32.59 [28.45-36.22]	4.95 [4.01-5.94]	23.5 [18.94-28.24]	-0.84 [-2 to 0.32]	-0.84
Libya	7.16 [5.35-9.7]	45.14 [33.58-60.9]	20.9 [15.14-28.31]	50.57 [37.02-68.89]	0.91 [0.67 to 1.14]	0.91
Qatar	0.35 [0.29-0.42]	78.63 [65.25-92.65]	1.05 [0.81-1.31]	32.26 [25.32-38.95]	-3.51 [-4.32 to -2.69]	-3.51
Jordan	8.88 [7.23-10.67]	96.08 [78.47-114.75]	23.48 [18.98-28.62]	47.99 [38.95-57.94]	-2.74 [-3.09 to -2.39]	-2.74
Lebanon	10.68 [8.4-13.83]	63.37 [50.26-82.25]	16.3 [13.12-19.32]	24.17 [19.47-28.72]	-3.29 [-3.54 to -3.05]	-3.29
Palestine	8.15 [6.56-10.02]	121.07 [97.42-146.7]	12.31 [10.37-14.42]	75.19 [63.03-87.79]	-1.59 [-1.91 to -1.26]	-1.59
Morocco	102.28 [75.88-133.6]	85.48 [63.62-111.24]	229.6 [173.99-290.22]	82.08 [62.22-102.92]	0 [-0.05 to 0.05]	0
Oman	3.45 [2.5-4.41]	66.09 [49.14-84.15]	6.11 [4.89-7.41]	49.78 [40.11-59.55]	-0.25 [-0.61 to 0.12]	-0.25
Syrian Arab Republic	29.17 [23.3-35.59]	71.18 [56.83-87.32]	54.08 [41.6-69.81]	58.4 [46.38-73.33]	-1.11 [-1.32 to -0.91]	-1.11
Saudi Arabia	36.26 [27.93-45.47]	85.41 [66.1-106.76]	67.8 [54.07-83.25]	61 [50.22-74.54]	-1.32 [-1.47 to -1.16]	-1.32
Tunisia	24.26 [18.92-31.11]	67.83 [53.31-86.51]	57.79 [39.36-78.47]	50.78 [34.93-68.25]	-1.13 [-1.26 to -1.01]	-1.13
United Arab Emirates	2.1 [1.61-2.66]	81.51 [64.19-100.8]	6 [4.58-7.48]	56.53 [45.64-68.57]	0.81 [0.13 to 1.49]	0.81
Turkey	190.76 [159.06-223.06]	71.62 [59.51-83.59]	322.8 [261.29-394.56]	40.25 [32.6-49.26]	-1.67 [-2.02 to -1.33]	-1.67
Afghanistan	56.85 [40.03-78.69]	106.08 [76.57-144.31]	68.57 [51.13-92.85]	98.36 [73.41-130.08]	-0.33 [-0.49 to -0.17]	-0.33
Yemen	34.88 [25.18-48.26]	101.72 [74.21-137.27]	96.37 [67.76-131.76]	94.6 [67.71-130.67]	-0.43 [-0.51 to -0.34]	-0.43
Bhutan	0.63 [0.41-0.87]	40.18 [25.12-55.33]	1.73 [1.32-2.17]	32.66 [24.97-40.93]	-0.71 [-0.79 to -0.63]	-0.71
Nepal	30.9 [21.2-40.71]	48.03 [34.03-62.85]	65.07 [47.95-90.02]	36.27 [26.94-48.59]	-0.95 [-1.09 to -0.8]	-0.95
Pakistan	191 [140.22-252.11]	42.9 [31.8-56.04]	374.77 [290.1-477.9]	43.7 [33.89-55.54]	-0.2 [-0.36 to -0.04]	-0.2
Bangladesh	253.27 [196.94-328]	68.85 [54.02-87.39]	708.78 [549.24-911.1]	65.2 [50.88-81.42]	-0.31 [-0.66 to 0.04]	-0.31
India	1086.98 [864.96-1391.71]	33.76 [27-42.44]	2821.17 [2367.91-3577.65]	29.11 [24.49-36.26]	-0.57 [-0.72 to -0.42]	-0.57
Angola	13.13 [10.07-16.71]	58.3 [45.07-72.05]	37.01 [29.12-46.79]	54.7 [43.45-68.08]	-0.48 [-0.59 to -0.38]	-0.48
Equatorial Guinea	0.8 [0.58-1.08]	64.16 [47.31-86.81]	1.6 [1.09-2.26]	49.85 [34.72-69.56]	-1.1 [-1.35 to -0.85]	-1.1
Congo	5 [3.74-6.32]	74.83 [57.42-91.68]	9.82 [7.54-12.34]	61.47 [47.46-76.7]	-0.87 [-0.98 to -0.76]	-0.87
Central African Republic	4.52 [3.19-6.12]	70.45 [49.86-93.4]	7.23 [4.96-10.3]	64.24 [44.9-89.67]	-0.37 [-0.43 to -0.3]	-0.37
Gabon	2.4 [1.87-3]	54.04 [41.28-67.98]	3.43 [2.6-4.48]	49.93 [38.32-64.1]	-0.38 [-0.54 to -0.23]	-0.38
Democratic Republic of the Congo	50.71 [35.83-68.49]	56.32 [40.67-74.71]	109.31 [72.84-161.26]	51.07 [34.35-74.71]	-0.44 [-0.49 to -0.39]	-0.44
Comoros	0.75 [0.55-0.96]	57.25 [43.99-71.69]	1.53 [1.16-1.98]	42.46 [32.53-54.76]	-1.25 [-1.44 to -1.06]	-1.25
Eritrea	2.86 [1.87-4.13]	52.43 [35.88-73.25]	7.21 [5.22-9.78]	47.99 [35.33-63.02]	-0.32 [-0.38 to -0.26]	-0.32
Djibouti	0.35 [0.24-0.5]	47.47 [34.72-67.82]	1.67 [1.22-2.32]	46.58 [35.26-62.59]	-0.16 [-0.2 to -0.12]	-0.16
Kenya	18.52 [13.49-24.38]	32.08 [22.98-42.05]	51.24 [37.78-65.85]	36.53 [26.55-46.92]	0.64 [0.54 to 0.74]	0.64
Burundi	12.88 [9.18-17.41]	72.94 [52.63-96.33]	14.03 [10.39-19.12]	46.51 [34.71-62.68]	-1.98 [-2.21 to -1.75]	-1.98
Madagascar	23.52 [18.87-28.68]	65.45 [52.32-79.08]	39.81 [28.34-52.48]	62.91 [45.78-81.65]	-0.22 [-0.27 to -0.16]	-0.22
Ethiopia	34.05 [22.07-53.31]	28.14 [18.94-41.58]	72.67 [55.98-96.49]	22.93 [17.53-30.29]	-0.88 [-0.96 to -0.8]	-0.88
Mozambique	26.48 [21.75-32.36]	63.58 [52.09-75.88]	61.24 [45.16-78.38]	80.01 [58.2-101.29]	1.17 [1.01 to 1.34]	1.17
Malawi	12.78 [10.02-15.51]	51.73 [40.85-63.11]	29.18 [23.18-36.69]	59.92 [47.45-74.68]	0.29 [0.07 to 0.51]	0.29
Somalia	5.87 [3.73-8.94]	44.32 [29.31-65.31]	12.08 [7.38-18.46]	37.25 [24.21-55.13]	-0.5 [-0.54 to -0.47]	-0.5
Rwanda	13.67 [10.55-17.62]	74.57 [58.34-94.54]	16.62 [11.74-22.1]	42.73 [30.08-56.93]	-2.63 [-3.01 to -2.25]	-2.63
Mauritius	5.02 [4.5-5.44]	86.62 [77.12-94.58]	5.1 [4.47-5.58]	30.93 [27.14-33.98]	-4.49 [-5.02 to -3.95]	-4.49
United Republic of Tanzania	26.41 [20.32-35.71]	36.18 [27.88-48.72]	83.7 [61.64-112]	46.31 [34.3-60.77]	0.79 [0.59 to 0.99]	0.79
Seychelles	0.3 [0.25-0.35]	53.27 [44.71-62.16]	0.35 [0.28-0.41]	37.11 [29.57-43.49]	-0.82 [-1.05 to -0.58]	-0.82
Lesotho	3.28 [2.5-4.28]	50.5 [37.99-66.36]	5.83 [4.29-7.61]	79.1 [59.54-99.52]	2.4 [1.9 to 2.9]	2.4
Uganda	20.32 [15.23-27.02]	45.94 [33.78-60.17]	33.55 [25.5-43.9]	34.61 [26.72-45.12]	-1.54 [-1.83 to -1.25]	-1.54
South Africa	64.91 [49.96-75.47]	39.06 [29.96-45.52]	174.28 [152.82-195.84]	50.2 [43.69-56.53]	0.88 [0.34 to 1.42]	0.88
Zambia	8.58 [6.25-11.97]	47.01 [34.74-64.37]	23.93 [17.46-32.26]	55.8 [41.14-73.24]	0.49 [0.38 to 0.6]	0.49
Botswana	2.58 [1.95-3.21]	74.08 [55.75-91.26]	4.67 [3.76-5.85]	48.17 [38.42-60.59]	-1.27 [-1.51 to -1.03]	-1.27
Namibia	3.22 [2.63-3.86]	78.85 [63.32-94.08]	6.38 [4.92-7.88]	68.91 [54-84.31]	-0.66 [-0.94 to -0.39]	-0.66
Zimbabwe	12.38 [10.11-14.74]	47.72 [38.92-56.77]	27.63 [22.09-34.3]	64.87 [52.64-78.24]	1.68 [1.13 to 2.22]	1.68
Benin	12.49 [10.1-15.43]	76.11 [61.47-94.12]	25.26 [20.49-31.58]	68.43 [55.81-85.21]	-0.3 [-0.38 to -0.21]	-0.3
Eswatini	1.18 [0.9-1.5]	64.83 [50.27-81.18]	2.28 [1.61-3.14]	65.11 [47.22-86.29]	0.5 [0.09 to 0.92]	0.5
Cameroon	16.54 [12.28-21.59]	54.69 [42.02-69.68]	53.88 [40.14-74.21]	64.21 [49.14-85.41]	0.62 [0.17 to 1.06]	0.62
Burkina Faso	12.34 [9.04-16.49]	41.69 [31.12-54.83]	28.01 [21.67-36.64]	42.74 [33.32-54.57]	0.35 [0.21 to 0.49]	0.35
Chad	13.94 [10.21-19.66]	60.48 [43.99-83.88]	28.61 [21.21-39.19]	70.59 [53.58-95.65]	0.47 [0.3 to 0.64]	0.47
Cabo Verde	1.07 [0.85-1.35]	44.2 [35.07-55.75]	2.35 [1.9-2.89]	58.21 [46.81-71.18]	0.59 [0.2 to 0.97]	0.59
Gambia	1.81 [1.34-2.35]	72.76 [56.04-94.3]	6.16 [4.54-8.15]	83.27 [61.68-109.08]	0.44 [0.35 to 0.52]	0.44
Guinea	16.21 [11.61-21.28]	61.18 [43.94-79.22]	30.62 [22.98-40.22]	70.43 [54.06-91.43]	0.75 [0.6 to 0.89]	0.75
Côte d'Ivoire	18.06 [14.26-22.2]	73.91 [60.57-88.17]	51.56 [39.56-67.85]	69.98 [55.28-88.55]	-0.22 [-0.4 to -0.04]	-0.22
Ghana	37.34 [29.24-48.01]	87.64 [68.71-111.85]	107.3 [81.76-134.99]	93.04 [71.72-116.26]	0.45 [0.23 to 0.67]	0.45
Guinea-Bissau	2.66 [1.97-3.51]	94.49 [72.69-120.38]	4.28 [3.29-5.48]	95.37 [74.47-119.95]	0.22 [0.15 to 0.29]	0.22
Liberia	6.09 [4.91-7.39]	69.75 [57.29-82.98]	9.67 [7.13-12.84]	68.32 [51.91-87.92]	-0.12 [-0.22 to -0.03]	-0.12
Togo	6.42 [5.16-7.98]	75.56 [61.55-92.84]	18.53 [13.46-23.96]	76.01 [57.07-97.59]	-0.09 [-0.23 to 0.06]	-0.09
Mauritania	6.3 [4.67-8.43]	80.51 [60.96-106.99]	10.93 [7.8-15.68]	65.99 [47.67-92.96]	-0.78 [-0.96 to -0.6]	-0.78
Senegal	18.85 [15.28-23.36]	76.56 [62.08-93.47]	41.89 [31.8-56.5]	72.67 [55.45-96.6]	-0.24 [-0.29 to -0.19]	-0.24
Nigeria	224.71 [160.75-311.93]	67.51 [49.33-91.92]	338.42 [268.31-426.35]	53.78 [43.65-66.96]	-0.8 [-0.93 to -0.67]	-0.8
Niger	8.79 [5.81-13.02]	52.01 [35.29-75.63]	27.24 [18.75-39.19]	53.27 [37.7-75.44]	0.23 [0.17 to 0.28]	0.23
Mali	13.34 [9.6-18.77]	52.09 [37.97-72.49]	29.28 [21.51-40.28]	49.84 [38.34-68.06]	0.08 [-0.05 to 0.2]	0.08
Sao Tome and Principe	0.32 [0.27-0.38]	59.5 [49.73-69.95]	0.54 [0.46-0.66]	67.43 [57.02-81.45]	0.6 [0.48 to 0.72]	0.6
Sierra Leone	13.49 [10.82-16.36]	80.52 [64.88-97.31]	21.08 [16.04-26.94]	74.79 [57.93-93.96]	-0.05 [-0.21 to 0.12]	-0.05
Bermuda	0.22 [0.18-0.25]	40.21 [33.67-46.27]	0.26 [0.21-0.32]	16.28 [13.19-19.89]	-3.07 [-3.26 to -2.88]	-3.07
Greenland	0.15 [0.12-0.18]	83.29 [68.52-98.4]	0.12 [0.1-0.15]	28.68 [22.61-36.62]	-3.71 [-3.91 to -3.51]	-3.71
Cook Islands	0.03 [0.03-0.04]	33.69 [27.06-41.08]	0.05 [0.04-0.06]	19.92 [15.45-25.33]	-1.8 [-1.95 to -1.64]	-1.8
American Samoa	0.05 [0.04-0.06]	36.38 [29.69-42.79]	0.1 [0.08-0.13]	29.81 [23.98-36.41]	-0.9 [-1.06 to -0.74]	-0.9
Monaco	0.58 [0.43-0.71]	66.33 [49.94-81.28]	0.35 [0.26-0.43]	26.31 [19.66-32.44]	-3.13 [-3.32 to -2.94]	-3.13
Guam	0.14 [0.12-0.17]	32.49 [26.23-38.84]	0.22 [0.18-0.26]	10.1 [8.25-11.9]	-3.32 [-3.77 to -2.87]	-3.32
Niue	0.01 [0.01-0.01]	48.15 [39.17-58.7]	0.01 [0.01-0.01]	40.08 [32.28-48.36]	-0.84 [-0.92 to -0.76]	-0.84
Nauru	0.02 [0.01-0.03]	72.79 [55.26-90.11]	0.03 [0.02-0.03]	66.2 [51.33-83.54]	-0.44 [-0.73 to -0.15]	-0.44
Palau	0.04 [0.03-0.05]	50.76 [39.3-63.58]	0.07 [0.05-0.08]	47.47 [38.23-58.11]	-0.03 [-0.13 to 0.06]	-0.03
Northern Mariana Islands	0.03 [0.03-0.04]	38.96 [30.83-48.59]	0.09 [0.07-0.1]	28.1 [22.88-33.1]	-1.55 [-1.84 to -1.26]	-1.55
Saint Kitts and Nevis	0.39 [0.33-0.44]	108.96 [92.36-123.81]	0.28 [0.23-0.32]	58.28 [47.69-66.67]	-1.82 [-2.01 to -1.63]	-1.82
Puerto Rico	8.04 [6.89-8.99]	24.97 [21.32-27.98]	7.35 [5.77-8.79]	7.65 [6.05-9.08]	-4.19 [-4.43 to -3.95]	-4.19
San Marino	0.16 [0.13-0.19]	42.26 [34.04-50.23]	0.14 [0.1-0.2]	12.46 [8.49-17.65]	-3.41 [-3.79 to -3.02]	-3.41
Tuvalu	0.03 [0.02-0.03]	57.94 [46.08-71.78]	0.04 [0.03-0.04]	45.47 [35.98-56.1]	-0.85 [-0.91 to -0.78]	-0.85
Tokelau	0.01 [0-0.01]	47.92 [35.34-60.84]	0.01 [0-0.01]	36 [27.54-45.47]	-1.05 [-1.12 to -0.98]	-1.05
United States Virgin Islands	0.2 [0.16-0.24]	34.66 [27.55-41.39]	0.27 [0.21-0.34]	15.77 [12.28-19.66]	-2.32 [-2.47 to -2.16]	-2.32
South Sudan	8.38 [5.81-11.4]	43.82 [31.35-58.89]	9.39 [6.69-12.57]	38.89 [28.5-51.5]	-0.54 [-0.72 to -0.36]	-0.54
Sudan	68.51 [48.17-88.88]	94.71 [67.9-121.01]	110.67 [78.99-146.86]	74.36 [53.41-97.18]	-0.92 [-0.97 to -0.88]	-0.92
Latin America & Caribbean - WB	521.11 [459.78-566.78]	25.16 [22-27.56]	1380.15 [1171.59-1547.15]	20.48 [17.37-22.96]	-2.11 [-2.73 to -1.48]	-2.11
East Asia & Pacific - WB	3056.87 [2619.53-3514.02]	34.38 [29.04-39.35]	14547.6 [12376.68-16776.84]	46.89 [39.69-54.07]	-0.47 [-1.13 to 0.21]	-0.47
Sub-Saharan Africa - WB	423.79 [326.02-551.22]	29.13 [22.51-37.5]	1797.38 [1555.37-2094.67]	53.05 [45.82-61.36]	-0.04 [-0.89 to 0.82]	-0.04
Europe & Central Asia - WB	5670.94 [5015.02-6142.54]	55.75 [49.02-60.63]	6680.58 [5831.83-7373.32]	35.24 [30.96-38.71]	-3.29 [-4.26 to -2.31]	-3.29
South Asia - WB	1685.2 [1376.34-2117.95]	40.89 [33.53-50.84]	4172.23 [3548.67-5150.96]	34.83 [29.74-42.36]	-0.63 [-0.74 to -0.52]	-0.63
Northern Africa	464.18 [378.51-582.3]	107.24 [87.23-132.89]	981.66 [809.55-1172.63]	87.31 [72.15-102.96]	-0.4 [-0.5 to -0.3]	-0.4
Middle East & North Africa - WB	835.31 [715.7-985.67]	93.9 [79.7-110.27]	1759.32 [1522.25-2014.16]	67.58 [58.07-77.46]	-1.07 [-1.11 to -1.04]	-1.07
Western Africa	393.66 [306.48-515.45]	67.68 [52.75-87.67]	741.65 [615.7-877.74]	60.78 [50.96-71.62]	-0.32 [-0.41 to -0.23]	-0.32
Eastern Africa	228.52 [189.59-284.47]	49.42 [41.43-60.7]	445.59 [364.11-535.02]	40.81 [32.71-48.91]	-0.79 [-0.86 to -0.73]	-0.79
America	1972.29 [1727.94-2152.71]	34.04 [29.61-37.32]	2415.12 [2029.43-2712.37]	17.27 [14.53-19.34]	-2.41 [-2.56 to -2.26]	-2.41
Southern Africa	148.52 [125.92-168.91]	47.24 [39.65-54.17]	372.42 [325.66-422.25]	56.87 [49.51-64.17]	0.72 [0.36 to 1.07]	0.72
Commonwealth Middle Income	1768.99 [1446.6-2219.09]	40.48 [33.15-49.97]	4168.46 [3583.07-5051.04]	33.9 [29.2-40.03]	-0.64 [-0.73 to -0.54]	-0.64
Commonwealth High Income	732.11 [647.36-793.25]	47.2 [41.47-51.37]	387.25 [314.65-445.49]	11.69 [9.58-13.4]	-4.81 [-5 to -4.62]	-4.81
Europe	8824.86 [7916.92-9471.3]	87.35 [77.95-94.11]	6430.37 [5607.59-7094.22]	34.51 [30.3-37.94]	-3.46 [-3.68 to -3.24]	-3.46
Commonwealth Low Income	384.94 [315.26-477.88]	60.03 [48.94-73.29]	1005.39 [782.61-1233.21]	59.57 [46.96-72.1]	-0.15 [-0.36 to 0.07]	-0.15
Africa	1342 [1160.22-1560.06]	68.06 [58.4-79.29]	2769.77 [2410.85-3162.09]	61.45 [53.27-69.66]	-0.26 [-0.37 to -0.15]	-0.26
Asia	8428.86 [7417.31-9668.73]	58.49 [51.18-66.26]	19800.6 [17264.12-22344.38]	44.22 [38.47-49.93]	-0.96 [-1.07 to -0.85]	-0.96
African Region	877.82 [735.56-1087.59]	57.25 [48.07-70.52]	1841.69 [1592.22-2138.39]	53.53 [46.28-61.68]	-0.23 [-0.36 to -0.1]	-0.23
South-East Asia Region	2196.55 [1864.36-2630.1]	45.58 [38.52-54.24]	5729.66 [5048.66-6612.62]	39.57 [34.57-45.41]	-0.55 [-0.63 to -0.46]	-0.55
Eastern Mediterranean Region	1073.53 [915.04-1258.05]	79.55 [67.22-93.05]	2143.34 [1849.45-2448.58]	65.07 [55.79-74.42]	-0.68 [-0.74 to -0.62]	-0.68
Region of the Americas	1972.29 [1727.94-2152.71]	34.04 [29.61-37.32]	2415.12 [2029.43-2712.37]	17.27 [14.53-19.34]	-2.41 [-2.56 to -2.26]	-2.41
European Region	9059.14 [8132.71-9723.72]	86.94 [77.59-93.69]	6739.45 [5884.81-7436.4]	35.22 [30.95-38.68]	-3.38 [-3.6 to -3.16]	-3.38
Advanced Health System	10852.4 [9677.38-11701.75]	67.82 [60.1-73.41]	8540.34 [7317.65-9524.15]	25.1 [21.73-27.79]	-3.66 [-3.86 to -3.46]	-3.66
Western Pacific Region	5331.25 [4610.08-6127.41]	64.74 [56.07-74.22]	12493.82 [10439.39-14491.09]	45.91 [38.43-53.29]	-1.1 [-1.25 to -0.96]	-1.1
Central Africa	107.12 [82.19-136.73]	59.37 [46.45-74.48]	228.45 [173.72-305.05]	55.97 [43.08-74.38]	-0.28 [-0.38 to -0.18]	-0.28
North America	948.26 [802.2-1053.71]	25.43 [21.45-28.29]	1042.85 [829.44-1198.73]	13.86 [11.11-15.86]	-2.55 [-2.89 to -2.22]	-2.55
Limited Health System	2526.72 [2123.86-3120.25]	45.79 [38.46-55.54]	5889.84 [5098.87-7058.84]	38.55 [33.46-45.59]	-0.68 [-0.76 to -0.6]	-0.68
Basic Health System	6935.75 [6164.53-7790.84]	64.52 [57.02-72.49]	16510.54 [14277.37-18839.7]	50.87 [43.59-58.11]	-0.73 [-0.89 to -0.56]	-0.73
Minimal Health System	261.82 [212.33-331.61]	64.36 [52.86-80.03]	483.42 [384.39-618.41]	59.8 [47.97-76.15]	-0.2 [-0.24 to -0.16]	-0.2
Southeast Asia, East Asia, and Oceania	5207.68 [4547.88-6035.73]	66.25 [57.66-77.09]	13690.45 [11645.07-15806.46]	56.39 [47.6-65.25]	-0.47 [-0.7 to -0.23]	-0.47
Central Europe, Eastern Europe, and Central Asia	5527.04 [5018.97-5874.28]	133.44 [119.68-142.63]	4652.88 [4125.41-5076.75]	69.74 [61.81-76.08]	-2.75 [-3.11 to -2.38]	-2.75
High-income	5530.11 [4836.03-6032.23]	45.52 [39.58-49.82]	3997.63 [3194.94-4623.93]	13.82 [11.18-15.82]	-4.14 [-4.32 to -3.96]	-4.14
Sub-Saharan Africa	810.46 [666.89-1015.02]	55.42 [45.58-69]	1683.33 [1450.96-1966.15]	52.23 [45.09-60.69]	-0.19 [-0.33 to -0.05]	-0.19
Latin America and Caribbean	837.99 [753.58-899.79]	47.52 [42.35-51.36]	1216.7 [1032.17-1367.63]	20.82 [17.65-23.42]	-2.63 [-2.74 to -2.53]	-2.63
World Bank Regions	11498.18 [10247.01-12581.69]	37.84 [33.04-41.53]	31380.1 [27698.29-34773.67]	38.52 [33.76-42.72]	-1.69 [-2.52 to -0.85]	-1.69
League of Arab States	756.84 [640.16-903.09]	99.42 [83.57-117.87]	1586.58 [1337.17-1866.43]	80.4 [68.04-94.06]	-0.65 [-0.7 to -0.61]	-0.65
Commonwealth	2886.04 [2458.99-3438.22]	45.76 [39.18-53.54]	5561.1 [4819.34-6599.89]	32.7 [28.32-38.17]	-1.19 [-1.26 to -1.11]	-1.19
WHO region	20510.59 [18581.42-22359.24]	64.64 [57.83-70.5]	31363.08 [27686.06-34749.1]	38.74 [33.96-42.97]	-1.86 [-1.95 to -1.76]	-1.86
European Union	4219.15 [3748.97-4541.24]	69.62 [61.65-75.23]	2674.17 [2271.45-3018.39]	21.58 [18.57-24.17]	-3.99 [-4.1 to -3.89]	-3.99
Gulf Cooperation Council	44.23 [34.73-54.59]	79.63 [62.43-98.09]	87.84 [72.51-104.7]	51.73 [43.66-61.15]	-1.46 [-1.65 to -1.28]	-1.46
Organization of Islamic Cooperation	2736.93 [2396.37-3111.58]	71.38 [61.44-80.97]	5918.59 [5277.62-6611.79]	64.37 [56.85-71.68]	-0.39 [-0.48 to -0.3]	-0.39
African Union	1342 [1160.22-1560.06]	68.06 [58.4-79.29]	2769.77 [2410.85-3162.09]	61.45 [53.27-69.66]	-0.26 [-0.37 to -0.15]	-0.26
OECD Countries	6678.48 [5884.73-7252.68]	49.96 [43.85-54.44]	5006.49 [4091.76-5733.36]	15.78 [13.14-17.92]	-4.01 [-4.18 to -3.84]	-4.01
Four World Regions	20568.01 [18633.63-22418.7]	64.59 [57.79-70.44]	31415.86 [27731.81-34807.39]	38.6 [33.83-42.8]	-1.87 [-1.96 to -1.77]	-1.87
G20	15562.79 [14033.66-16966.03]	62.58 [55.88-68.2]	22949.05 [19989.75-25884.43]	35.66 [30.99-40.19]	-2.03 [-2.14 to -1.92]	-2.03
Nordic Region	189.07 [164.25-207.69]	44.94 [38.99-49.39]	104.77 [84.67-119.6]	14.18 [11.61-16.17]	-3.9 [-4.05 to -3.75]	-3.9
Association of Southeast Asian Nations	1125.99 [975.85-1278.34]	64.1 [54.76-73.59]	2898.28 [2461.19-3357.92]	59.24 [50.53-68.08]	-0.21 [-0.37 to -0.04]	-0.21
Sahel Region	315.76 [255.54-421.7]	67.77 [55.3-89.16]	567.31 [470.19-691.75]	59.89 [50.7-72.35]	-0.41 [-0.5 to -0.33]	-0.41
Health System Grouping Levels	20576.68 [18641-22428.18]	64.55 [57.75-70.39]	31424.15 [27738.31-34815.99]	38.56 [33.8-42.76]	-1.87 [-1.96 to -1.77]	-1.87

IS-HBMI death and ASMR						
	1990		2021		1990-2021	
	Death cases	ASMR per 100,000	Death cases	ASMR per 100,000	EAPC of ASMR	
	No. *102 (95% UI)	No. (95% UI)	No. *102 (95% UI)	No.(95% UI)	No. (95% CI)	
Overall	880.75 [126.36-1763.67]	2.57 [0.37-5.15]	1723.91 [250.65-3479.13]	2.06 [0.3-4.17]	-1.1 [-1.25 to -0.96]	-1.1
Sex						
Female	548.68 [78.72-1099.74]	2.79 [0.4-5.68]	950.15 [138.51-1915.59]	2.03 [0.3-4.09]	-1.47 [-1.63 to -1.3]	-1.47
Male	332.07 [47.65-655.64]	2.18 [0.31-4.35]	773.76 [112.04-1544.98]	2.05 [0.29-4.09]	-0.5 [-0.61 to -0.39]	-0.5
Socio-demographic index						
High SDI	246.49 [35.11-501.64]	2.21 [0.31-4.5]	265.99 [38.46-563.72]	1.12 [0.17-2.3]	-2.6 [-2.81 to -2.4]	-2.6
High-middle SDI	429.15 [62.01-848.69]	4.93 [0.71-9.84]	656.96 [95.45-1334.97]	3.35 [0.49-6.84]	-1.83 [-2.1 to -1.55]	-1.83
Middle SDI	120.42 [16.65-237.61]	1.37 [0.19-2.77]	488.73 [71.06-958.23]	1.96 [0.28-3.91]	1.08 [1.02 to 1.13]	1.08
Low-middle SDI	67.35 [9.92-133.66]	1.29 [0.19-2.6]	253.75 [37.62-500.69]	1.93 [0.28-3.84]	1.43 [1.37 to 1.48]	1.43
Low SDI	15.21 [1.91-28.74]	0.77 [0.1-1.48]	56.01 [7.18-106.17]	1.27 [0.16-2.47]	1.54 [1.47 to 1.6]	1.54
Region						
Andean Latin America	2.18 [0.32-4.57]	1.13 [0.16-2.4]	5.91 [0.85-11.76]	1.03 [0.15-2.06]	-0.52 [-0.72 to -0.33]	-0.52
Australasia	4.28 [0.61-8.77]	1.89 [0.27-3.94]	5.48 [0.77-12.5]	0.85 [0.12-1.91]	-2.85 [-2.97 to -2.73]	-2.85
Caribbean	3.8 [0.56-7.37]	1.56 [0.23-3.04]	10.1 [1.48-19.99]	1.85 [0.27-3.66]	0.59 [0.52 to 0.67]	0.59
Central Asia	21.08 [3.1-41.65]	4.88 [0.71-9.7]	36 [5.44-72.83]	5.06 [0.76-10.32]	-0.44 [-0.78 to -0.09]	-0.44
Central Europe	111.19 [16.39-225.01]	8.06 [1.18-16.57]	111.72 [16.38-233.62]	4.68 [0.69-9.74]	-2.13 [-2.3 to -1.96]	-2.13
Central Latin America	12.41 [1.84-24.45]	1.68 [0.25-3.35]	31.9 [4.98-65.03]	1.33 [0.21-2.75]	-0.99 [-1.19 to -0.8]	-0.99
Central Sub-Saharan Africa	1.58 [0.19-3.24]	0.86 [0.1-1.75]	7.89 [1.04-16.05]	1.91 [0.25-3.94]	2.44 [2.35 to 2.52]	2.44
East Asia	74.32 [9.44-150.38]	0.98 [0.12-1.99]	451.1 [61.94-933.3]	2.17 [0.3-4.49]	2.64 [2.46 to 2.82]	2.64
Eastern Europe	250.77 [36.56-493.8]	9.66 [1.4-19.22]	263.97 [38.5-526.49]	7.37 [1.07-14.65]	-1.7 [-2.22 to -1.17]	-1.7
Eastern Sub-Saharan Africa	3.47 [0.41-6.56]	0.55 [0.07-1.08]	15.33 [1.96-30.54]	1.1 [0.14-2.25]	2.18 [2.14 to 2.22]	2.18
High-income Asia Pacific	15.29 [2.05-30.28]	0.85 [0.11-1.68]	20.99 [2.7-43.49]	0.34 [0.05-0.68]	-3.43 [-3.6 to -3.25]	-3.43
High-income North America	56.37 [8.05-116.99]	1.53 [0.22-3.17]	89.77 [12.77-189.88]	1.24 [0.18-2.58]	-1.33 [-1.68 to -0.97]	-1.33
North Africa and Middle East	80.85 [12.32-161.31]	5.5 [0.82-11.16]	258.11 [42.03-509.56]	6.46 [1.02-12.99]	0.51 [0.47 to 0.55]	0.51
Oceania	0.36 [0.05-0.75]	1.49 [0.2-3.18]	1.02 [0.15-2.06]	1.62 [0.23-3.4]	0.1 [0.03 to 0.18]	0.1
South Asia	16.86 [1.88-34.15]	0.34 [0.04-0.68]	102.72 [12.94-201.32]	0.76 [0.09-1.5]	2.63 [2.58 to 2.67]	2.63
Southeast Asia	13.73 [1.8-26.04]	0.59 [0.08-1.13]	76.9 [10.38-149.16]	1.27 [0.17-2.52]	2.62 [2.47 to 2.77]	2.62
Southern Latin America	12.48 [1.81-25.23]	2.89 [0.42-5.9]	13.72 [2.01-28.87]	1.5 [0.22-3.15]	-1.84 [-1.97 to -1.7]	-1.84
Southern Sub-Saharan Africa	5.04 [0.73-9.9]	2.14 [0.31-4.38]	20.86 [3.04-41.39]	4.43 [0.64-8.85]	2.55 [2 to 3.11]	2.55
Tropical Latin America	26.41 [3.86-52.52]	3.23 [0.46-6.64]	48.96 [7.09-100.16]	1.96 [0.28-4.03]	-1.57 [-1.69 to -1.45]	-1.57
Western Europe	156.3 [21.99-322.11]	2.59 [0.37-5.34]	106.82 [14.67-228.43]	0.89 [0.12-1.89]	-3.68 [-3.84 to -3.52]	-3.68
Western Sub-Saharan Africa	11.96 [1.54-24.04]	1.59 [0.21-3.27]	44.66 [6.05-87.28]	2.78 [0.37-5.46]	1.75 [1.63 to 1.86]	1.75
Indonesia	4.38 [0.54-8.69]	0.49 [0.06-0.95]	33.8 [4.39-68.51]	1.58 [0.2-3.3]	4.12 [3.88 to 4.35]	4.12
Democratic People's Republic of Korea	0.97 [0.12-2.18]	0.75 [0.09-1.75]	5.92 [0.78-13.84]	1.99 [0.26-4.64]	3.13 [3.11 to 3.16]	3.13
Cambodia	0.19 [0.02-0.37]	0.47 [0.06-0.92]	0.82 [0.12-1.62]	0.76 [0.1-1.56]	1.43 [1.27 to 1.59]	1.43
Taiwan (Province of China)	1.64 [0.23-3.23]	1.18 [0.16-2.37]	2.55 [0.36-5.26]	0.58 [0.08-1.2]	-2.33 [-2.59 to -2.08]	-2.33
China	71.71 [9.09-145.27]	0.98 [0.12-1.99]	442.63 [60.69-916.58]	2.21 [0.3-4.58]	2.7 [2.5 to 2.89]	2.7
Malaysia	1.09 [0.16-2.08]	1.23 [0.18-2.37]	4.58 [0.63-9.15]	1.77 [0.24-3.56]	1.36 [1.22 to 1.51]	1.36
Lao People's Democratic Republic	0.18 [0.02-0.36]	0.94 [0.12-1.96]	0.57 [0.07-1.17]	1.37 [0.17-2.83]	1.22 [1.2 to 1.24]	1.22
Maldives	0.01 [0-0.02]	0.98 [0.12-1.94]	0.03 [0-0.05]	0.81 [0.11-1.61]	-1.05 [-1.25 to -0.85]	-1.05
Philippines	1.66 [0.21-3.27]	0.66 [0.09-1.35]	9.65 [1.26-19.12]	1.28 [0.17-2.51]	2.33 [2.09 to 2.58]	2.33
Thailand	1.87 [0.24-3.66]	0.56 [0.07-1.11]	10 [1.47-20.2]	0.92 [0.14-1.86]	1.03 [0.74 to 1.32]	1.03
Sri Lanka	1.01 [0.13-2.04]	1.15 [0.15-2.44]	4.2 [0.6-9.33]	1.75 [0.25-4.08]	1.89 [1.63 to 2.14]	1.89
Viet Nam	1.12 [0.13-2.29]	0.31 [0.04-0.64]	7.92 [0.93-15.78]	0.89 [0.1-1.88]	4.09 [3.83 to 4.36]	4.09
Myanmar	1.98 [0.26-3.94]	0.87 [0.11-1.74]	4.86 [0.67-9.85]	1.04 [0.15-2.16]	0.37 [0.25 to 0.48]	0.37
Timor-Leste	0 [0-0.01]	0.19 [0.02-0.44]	0.04 [0-0.08]	0.46 [0.06-0.96]	3.19 [2.81 to 3.56]	3.19
Fiji	0.07 [0.01-0.15]	2.61 [0.35-5.51]	0.21 [0.03-0.43]	3.69 [0.56-7.57]	0.74 [0.51 to 0.98]	0.74
Papua New Guinea	0.14 [0.02-0.32]	0.86 [0.11-2]	0.46 [0.06-1.02]	1 [0.13-2.22]	0.32 [0.18 to 0.46]	0.32
Marshall Islands	0 [0-0.01]	3.43 [0.49-7.61]	0.01 [0-0.02]	4.26 [0.62-8.92]	0.59 [0.52 to 0.66]	0.59
Kiribati	0.01 [0-0.02]	2.26 [0.32-4.63]	0.02 [0-0.05]	3.79 [0.57-7.77]	1.64 [1.57 to 1.71]	1.64
Micronesia (Federated States of)	0.02 [0-0.03]	3.54 [0.51-7.44]	0.03 [0-0.06]	4.34 [0.63-9.07]	0.58 [0.52 to 0.65]	0.58
Solomon Islands	0.02 [0-0.04]	1.46 [0.18-3.26]	0.06 [0.01-0.14]	2.12 [0.28-4.94]	1.22 [1.02 to 1.42]	1.22
Samoa	0.02 [0-0.05]	2.95 [0.46-6.09]	0.05 [0.01-0.09]	3.51 [0.53-6.97]	0.46 [0.37 to 0.56]	0.46
Tonga	0.01 [0-0.02]	1.93 [0.28-3.91]	0.02 [0-0.04]	2.68 [0.43-5.39]	1.17 [1.04 to 1.31]	1.17
Vanuatu	0.01 [0-0.02]	1.8 [0.24-4.08]	0.03 [0-0.08]	2.32 [0.31-5.18]	0.64 [0.56 to 0.71]	0.64
Armenia	1.1 [0.17-2.2]	4.6 [0.69-9.36]	1.56 [0.24-3.23]	3.62 [0.56-7.54]	-1.81 [-2.2 to -1.41]	-1.81
Georgia	2.3 [0.33-4.8]	3.97 [0.56-8.26]	3.64 [0.51-7.37]	5.71 [0.81-11.46]	1.13 [0.55 to 1.72]	1.13
Azerbaijan	1.2 [0.17-2.44]	2.65 [0.38-5.43]	2.38 [0.34-5.1]	2.78 [0.39-5.8]	0.25 [-0.09 to 0.59]	0.25
Kyrgyzstan	1.8 [0.27-3.51]	6.51 [0.96-12.89]	2.34 [0.38-4.59]	5.11 [0.81-10.07]	-1.45 [-1.92 to -0.97]	-1.45
Kazakhstan	8.11 [1.2-16.25]	7.17 [1.05-14.48]	10.56 [1.57-21.77]	7.34 [1.08-15.42]	-0.59 [-1.16 to -0.03]	-0.59
Tajikistan	1.18 [0.16-2.37]	4.65 [0.64-9.34]	2.16 [0.31-4.37]	4.65 [0.66-9.41]	-0.35 [-0.78 to 0.09]	-0.35
Mongolia	0.1 [0.02-0.21]	1.08 [0.16-2.16]	0.25 [0.03-0.51]	1.28 [0.16-2.7]	0.46 [0 to 0.93]	0.46
Uzbekistan	4.41 [0.65-8.64]	4 [0.58-7.81]	10.61 [1.65-21.8]	4.63 [0.71-9.93]	-0.2 [-0.6 to 0.2]	-0.2
Turkmenistan	0.87 [0.14-1.8]	4.8 [0.74-9.92]	2.5 [0.36-5.26]	6.63 [0.96-14.12]	0.76 [0.25 to 1.27]	0.76
Albania	0.49 [0.07-1.04]	2.89 [0.42-6.21]	1.18 [0.16-2.57]	2.82 [0.39-6.18]	0.31 [0.05 to 0.58]	0.31
Bosnia and Herzegovina	2.18 [0.31-4.51]	6.13 [0.85-12.72]	3.78 [0.56-7.94]	5.86 [0.87-12.3]	-0.57 [-0.73 to -0.41]	-0.57
Bulgaria	10.55 [1.54-20.88]	10.46 [1.48-20.97]	15.57 [2.31-32.37]	10.62 [1.58-22.16]	0.22 [0.04 to 0.4]	0.22
Croatia	3.82 [0.54-7.86]	7.28 [1.02-15.16]	3.33 [0.48-7.27]	3.32 [0.48-7.18]	-2.72 [-2.9 to -2.55]	-2.72
Hungary	12.92 [1.94-26.24]	9.11 [1.36-18.75]	7.43 [1.1-14.75]	3.55 [0.53-7.01]	-3.46 [-3.68 to -3.24]	-3.46
Czechia	14.44 [2.12-29.87]	10.54 [1.54-21.92]	5.22 [0.78-11.17]	2.22 [0.33-4.71]	-5.39 [-5.76 to -5.02]	-5.39
Poland	29.16 [4.28-58.39]	7.09 [1.03-14.1]	23.48 [3.6-50.81]	3.02 [0.46-6.47]	-3.18 [-3.31 to -3.05]	-3.18
Montenegro	0.14 [0.02-0.29]	2.42 [0.35-5.11]	0.42 [0.06-0.89]	4.81 [0.65-10.53]	2.51 [2.39 to 2.64]	2.51
North Macedonia	1.79 [0.26-3.55]	10.84 [1.59-21.88]	3.58 [0.53-7.53]	13.89 [2-29.56]	0.54 [0.08 to 1.01]	0.54
Romania	19.15 [2.74-38.32]	7.99 [1.12-16.09]	25.1 [3.46-52.26]	6.09 [0.84-12.6]	-1.48 [-1.8 to -1.15]	-1.48
Slovakia	4.08 [0.58-8.36]	6.99 [0.99-14.43]	3.8 [0.6-7.77]	3.89 [0.61-7.97]	-2.03 [-2.13 to -1.93]	-2.03
Serbia	9.38 [1.45-19.56]	10.76 [1.64-22.59]	16.26 [2.38-33.41]	9.24 [1.36-18.95]	-1.19 [-1.51 to -0.87]	-1.19
Ukraine	61.32 [8.79-121.76]	9.01 [1.29-18.1]	51.11 [7.95-101.9]	6.45 [1.01-12.88]	-1.8 [-2.14 to -1.47]	-1.8
Slovenia	1.32 [0.19-2.7]	5.41 [0.78-11.09]	0.95 [0.14-2.09]	1.75 [0.26-3.83]	-3.66 [-3.9 to -3.42]	-3.66
Estonia	1.78 [0.26-3.62]	8.84 [1.3-17.85]	0.6 [0.08-1.25]	1.97 [0.28-4.05]	-6.37 [-7 to -5.74]	-6.37
Russian Federation	172.23 [25.23-341.23]	10.48 [1.52-21.05]	193.27 [28.15-396.02]	8 [1.16-16.33]	-1.76 [-2.37 to -1.14]	-1.76
Lithuania	2.19 [0.33-4.4]	4.89 [0.74-9.88]	2.78 [0.39-6.13]	4.27 [0.61-9.12]	-0.68 [-1.05 to -0.32]	-0.68
Belarus	7.99 [1.15-15.85]	6.26 [0.9-12.57]	10.02 [1.5-20.3]	6.08 [0.91-12.33]	-0.92 [-1.44 to -0.4]	-0.92
Latvia	3.46 [0.52-7.15]	9.78 [1.46-20.37]	3.12 [0.45-6.48]	6.82 [0.99-13.92]	-1.68 [-1.97 to -1.4]	-1.68
Republic of Moldova	1.8 [0.27-3.62]	4.98 [0.73-10.02]	3.05 [0.44-6.13]	4.99 [0.73-10.04]	0.21 [-0.29 to 0.71]	0.21
New Zealand	0.81 [0.12-1.65]	2.09 [0.3-4.26]	1 [0.14-2.17]	1.05 [0.15-2.28]	-2.38 [-2.55 to -2.21]	-2.38
Japan	11.94 [1.58-23.55]	0.77 [0.1-1.52]	15.5 [2-32.54]	0.29 [0.04-0.59]	-3.47 [-3.64 to -3.29]	-3.47
Singapore	0.15 [0.02-0.3]	0.74 [0.1-1.48]	0.18 [0.02-0.36]	0.21 [0.03-0.44]	-4.12 [-4.6 to -3.64]	-4.12
Australia	3.46 [0.49-7.22]	1.86 [0.26-3.91]	4.48 [0.63-10.27]	0.82 [0.12-1.84]	-2.94 [-3.07 to -2.81]	-2.94
Brunei Darussalam	0.01 [0-0.02]	0.99 [0.13-1.93]	0.03 [0-0.05]	0.89 [0.12-1.8]	-0.3 [-0.5 to -0.11]	-0.3
Republic of Korea	3.19 [0.44-6.74]	1.4 [0.19-2.97]	5.28 [0.67-11.22]	0.58 [0.07-1.23]	-3.65 [-3.91 to -3.39]	-3.65
Austria	3.53 [0.48-7.45]	2.84 [0.39-5.98]	1.5 [0.2-3.31]	0.66 [0.09-1.46]	-5.12 [-5.5 to -4.74]	-5.12
Cyprus	0.15 [0.02-0.3]	2.83 [0.38-5.85]	0.23 [0.04-0.52]	1.45 [0.22-3.27]	-2.55 [-2.81 to -2.28]	-2.55
Andorra	0.01 [0-0.01]	1.1 [0.16-2.47]	0.01 [0-0.03]	0.64 [0.09-1.41]	-1.51 [-1.77 to -1.26]	-1.51
Belgium	3.06 [0.43-6.22]	1.94 [0.27-3.92]	2.23 [0.3-4.89]	0.75 [0.11-1.62]	-2.99 [-3.1 to -2.88]	-2.99
Denmark	1.53 [0.22-3.13]	1.75 [0.25-3.57]	1.14 [0.16-2.42]	0.82 [0.11-1.73]	-2.84 [-3.05 to -2.62]	-2.84
France	12.04 [1.69-24]	1.36 [0.19-2.68]	12.72 [1.7-27.34]	0.65 [0.09-1.37]	-2.33 [-2.43 to -2.23]	-2.33
Italy	19.98 [2.8-40.75]	2.25 [0.31-4.6]	18.69 [2.48-41.28]	0.91 [0.12-1.98]	-3.21 [-3.46 to -2.96]	-3.21
Finland	1.97 [0.29-4.05]	2.7 [0.39-5.58]	1.68 [0.23-3.51]	1.04 [0.15-2.15]	-3.13 [-3.25 to -3.01]	-3.13
Greece	5.22 [0.73-11.07]	3.6 [0.5-7.72]	5.33 [0.75-12.04]	1.57 [0.22-3.49]	-3.45 [-3.82 to -3.07]	-3.45
Ireland	0.94 [0.14-1.95]	2.36 [0.34-4.92]	0.63 [0.09-1.4]	0.73 [0.1-1.61]	-3.86 [-4.09 to -3.62]	-3.86
Norway	1.55 [0.22-3.2]	2 [0.28-4.13]	0.71 [0.1-1.48]	0.57 [0.08-1.18]	-4.39 [-4.57 to -4.21]	-4.39
Germany	44.23 [6.11-90]	3.31 [0.46-6.72]	23.63 [3.33-51.22]	1.03 [0.15-2.2]	-3.91 [-4.18 to -3.63]	-3.91
Malta	0.08 [0.01-0.16]	1.99 [0.27-3.89]	0.08 [0.01-0.19]	0.73 [0.11-1.59]	-3.41 [-3.74 to -3.07]	-3.41
Iceland	0.06 [0.01-0.12]	1.88 [0.28-3.91]	0.05 [0.01-0.11]	0.7 [0.1-1.53]	-3.3 [-3.5 to -3.1]	-3.3
Israel	0.74 [0.11-1.53]	1.63 [0.23-3.39]	0.81 [0.11-1.73]	0.57 [0.08-1.19]	-4.01 [-4.22 to -3.8]	-4.01
Luxembourg	0.2 [0.03-0.41]	3.84 [0.52-7.69]	0.11 [0.01-0.23]	0.85 [0.12-1.83]	-4.76 [-4.86 to -4.66]	-4.76
Sweden	3.01 [0.41-6.52]	1.77 [0.24-3.81]	1.94 [0.27-4.29]	0.7 [0.1-1.52]	-3.23 [-3.5 to -2.96]	-3.23
Spain	16.64 [2.35-34.55]	3.11 [0.44-6.48]	11.38 [1.59-25.49]	0.86 [0.13-1.9]	-4.32 [-4.55 to -4.08]	-4.32
Netherlands	3.57 [0.49-7.56]	1.73 [0.24-3.68]	3.8 [0.55-8.12]	0.94 [0.14-1.99]	-2.55 [-2.86 to -2.25]	-2.55
Portugal	6.95 [0.92-14.36]	5.41 [0.71-11.5]	4.57 [0.61-9.91]	1.42 [0.19-3.08]	-5 [-5.26 to -4.74]	-5
United States of America	51.31 [7.32-105.65]	1.53 [0.22-3.14]	83.71 [11.92-177.1]	1.3 [0.19-2.72]	-1.16 [-1.52 to -0.8]	-1.16
United Kingdom	28.86 [4.16-59.83]	2.98 [0.43-6.17]	14.28 [1.99-31.34]	0.91 [0.13-1.97]	-4.16 [-4.4 to -3.92]	-4.16
Argentina	8.26 [1.19-16.72]	2.73 [0.39-5.55]	8.12 [1.19-17.25]	1.39 [0.2-2.93]	-1.87 [-2.07 to -1.67]	-1.87
Switzerland	1.81 [0.25-3.95]	1.57 [0.22-3.4]	1.18 [0.15-2.6]	0.49 [0.07-1.06]	-3.67 [-3.83 to -3.51]	-3.67
Uruguay	1.39 [0.2-2.83]	3.56 [0.52-7.28]	1.35 [0.2-2.91]	2.09 [0.31-4.43]	-1.94 [-2.17 to -1.71]	-1.94
Canada	5.05 [0.73-10.6]	1.58 [0.23-3.33]	6.05 [0.84-13.4]	0.71 [0.1-1.56]	-3.08 [-3.32 to -2.84]	-3.08
Chile	2.84 [0.41-5.78]	3.12 [0.45-6.43]	4.24 [0.61-8.69]	1.61 [0.23-3.29]	-1.7 [-1.9 to -1.49]	-1.7
Antigua and Barbuda	0.01 [0-0.02]	2.01 [0.3-4.13]	0.02 [0-0.04]	2.1 [0.31-4.25]	-0.06 [-0.29 to 0.17]	-0.06
Barbados	0.09 [0.01-0.18]	2.95 [0.42-6]	0.15 [0.02-0.32]	2.94 [0.44-6.19]	-0.21 [-0.39 to -0.03]	-0.21
Cuba	1.28 [0.19-2.44]	1.3 [0.19-2.5]	3.91 [0.56-7.83]	1.91 [0.28-3.81]	1.37 [1.23 to 1.5]	1.37
Bahamas	0.03 [0-0.05]	1.94 [0.28-3.87]	0.07 [0.01-0.14]	2.02 [0.28-4.04]	0.15 [0.01 to 0.28]	0.15
Belize	0.01 [0-0.03]	1.54 [0.22-3.18]	0.05 [0.01-0.1]	1.97 [0.31-4.01]	0.43 [-0.17 to 1.04]	0.43
Dominica	0.02 [0-0.04]	3.75 [0.53-7.53]	0.03 [0.01-0.07]	4.42 [0.67-9.12]	0.49 [0.41 to 0.57]	0.49
Grenada	0.02 [0-0.05]	3.17 [0.45-6.38]	0.03 [0-0.05]	2.57 [0.34-5.26]	-0.5 [-0.71 to -0.29]	-0.5
Bolivia (Plurinational State of)	0.44 [0.06-0.92]	1.5 [0.2-3.14]	1.26 [0.17-2.77]	1.54 [0.2-3.38]	0.08 [-0.04 to 0.2]	0.08
Haiti	0.34 [0.04-0.7]	1.16 [0.15-2.38]	1.17 [0.15-2.49]	1.82 [0.23-3.9]	1.76 [1.64 to 1.89]	1.76
Dominican Republic	0.34 [0.05-0.69]	0.99 [0.13-2.06]	1.75 [0.27-3.6]	1.78 [0.28-3.67]	2.21 [2.01 to 2.41]	2.21
Saint Lucia	0.03 [0-0.05]	3.17 [0.46-6.49]	0.06 [0.01-0.12]	2.4 [0.34-4.98]	-1.56 [-1.97 to -1.15]	-1.56
Suriname	0.03 [0-0.07]	1.45 [0.2-2.87]	0.11 [0.02-0.24]	1.88 [0.27-3.9]	0.82 [0.5 to 1.13]	0.82
Guyana	0.15 [0.02-0.31]	4.35 [0.59-8.62]	0.22 [0.03-0.44]	3.88 [0.58-8.09]	0.27 [0.02 to 0.52]	0.27
Jamaica	0.45 [0.06-0.89]	2.46 [0.35-4.77]	0.94 [0.15-1.92]	2.88 [0.45-5.94]	0.76 [0.36 to 1.16]	0.76
Saint Vincent and the Grenadines	0.01 [0-0.02]	1.8 [0.24-3.67]	0.03 [0-0.05]	2.03 [0.3-4.07]	0.5 [0.26 to 0.73]	0.5
Trinidad and Tobago	0.27 [0.04-0.51]	3.53 [0.53-6.94]	0.51 [0.07-0.99]	2.68 [0.38-5.27]	-1.33 [-1.58 to -1.08]	-1.33
Ecuador	0.74 [0.11-1.49]	1.47 [0.21-2.98]	1.95 [0.27-3.95]	1.28 [0.18-2.62]	-0.26 [-0.6 to 0.09]	-0.26
Peru	1 [0.15-2.19]	0.89 [0.13-1.98]	2.71 [0.4-5.57]	0.81 [0.12-1.66]	-0.93 [-1.38 to -0.47]	-0.93
Colombia	2.37 [0.34-4.58]	1.51 [0.21-2.98]	5.24 [0.78-11.06]	0.94 [0.14-1.97]	-2.08 [-2.39 to -1.77]	-2.08
El Salvador	0.38 [0.05-0.78]	1.31 [0.18-2.75]	0.79 [0.11-1.64]	1.2 [0.17-2.49]	-0.54 [-0.89 to -0.19]	-0.54
Mexico	6.85 [1.01-13.59]	1.85 [0.27-3.69]	15.55 [2.48-30.88]	1.32 [0.21-2.64]	-1.23 [-1.38 to -1.07]	-1.23
Honduras	0.31 [0.04-0.63]	1.66 [0.23-3.52]	1.73 [0.24-3.63]	3.16 [0.45-6.7]	2.27 [2.05 to 2.49]	2.27
Nicaragua	0.21 [0.03-0.41]	1.49 [0.22-2.95]	0.55 [0.09-1.12]	1.27 [0.2-2.61]	-0.46 [-0.63 to -0.29]	-0.46
Costa Rica	0.18 [0.03-0.38]	1.11 [0.15-2.29]	0.55 [0.08-1.15]	0.97 [0.15-2.04]	-0.97 [-1.38 to -0.56]	-0.97
Venezuela (Bolivarian Republic of)	1.46 [0.22-2.99]	1.71 [0.25-3.55]	5.54 [0.82-11.58]	2 [0.29-4.14]	0.19 [-0.14 to 0.52]	0.19
Guatemala	0.36 [0.06-0.69]	1.34 [0.2-2.68]	1.07 [0.16-2.19]	1.12 [0.16-2.32]	-1.04 [-1.37 to -0.72]	-1.04
Iran (Islamic Republic of)	8.48 [1.26-16.12]	3.67 [0.54-7.1]	29 [4.43-56.27]	4.12 [0.62-8.09]	0.17 [0.01 to 0.33]	0.17
Brazil	25.94 [3.79-51.56]	3.25 [0.47-6.69]	47.65 [6.9-97.33]	1.95 [0.28-4]	-1.61 [-1.73 to -1.5]	-1.61
Panama	0.3 [0.04-0.63]	2.17 [0.31-4.66]	0.88 [0.14-1.8]	1.91 [0.3-3.91]	-0.59 [-0.79 to -0.39]	-0.59
Algeria	3.48 [0.49-7.28]	3.68 [0.51-7.97]	14.71 [2.41-30.33]	5.3 [0.84-11.07]	1.25 [1.2 to 1.3]	1.25
Egypt	24.71 [3.75-52.16]	11.74 [1.72-25.44]	78.75 [13.59-152.28]	15.81 [2.58-31.83]	1.38 [1.21 to 1.55]	1.38
Paraguay	0.47 [0.07-0.95]	2.28 [0.33-4.68]	1.31 [0.18-2.76]	2.44 [0.34-5.17]	0.38 [0.22 to 0.54]	0.38
Bahrain	0.06 [0.01-0.12]	4.68 [0.65-9.59]	0.26 [0.04-0.52]	4.79 [0.78-9.95]	-0.54 [-0.97 to -0.12]	-0.54
Tunisia	1.18 [0.17-2.44]	2.66 [0.37-5.58]	5.16 [0.8-11.06]	4.22 [0.64-9.17]	1.35 [1.22 to 1.48]	1.35
Jordan	0.95 [0.14-1.88]	8.45 [1.24-16.76]	3.47 [0.57-6.85]	5.96 [0.95-12.44]	-1.78 [-2.2 to -1.36]	-1.78
Lebanon	0.84 [0.11-1.77]	4.39 [0.57-9.11]	1.57 [0.23-3.3]	2.42 [0.36-5.06]	-2.15 [-2.48 to -1.83]	-2.15
Iraq	7.31 [1.05-14.81]	9.66 [1.37-19.69]	21.37 [3.44-43.09]	10.84 [1.68-22.63]	-0.21 [-0.41 to -0.02]	-0.21
Kuwait	0.14 [0.02-0.26]	2.59 [0.39-5.11]	0.75 [0.12-1.44]	2.98 [0.46-6.01]	0.72 [-0.4 to 1.86]	0.72
Morocco	5.35 [0.71-11.66]	3.98 [0.52-8.6]	19.64 [2.86-40.56]	6.19 [0.9-12.81]	1.57 [1.49 to 1.64]	1.57
Afghanistan	2.69 [0.34-5.41]	3.94 [0.5-7.87]	5.09 [0.8-11.13]	5.6 [0.85-12.1]	0.99 [0.86 to 1.11]	0.99
Libya	0.5 [0.07-1.04]	2.85 [0.41-6]	3.04 [0.49-5.99]	6.38 [1-12.91]	3.06 [2.85 to 3.27]	3.06
Yemen	1.18 [0.15-2.43]	2.64 [0.34-5.45]	6.95 [0.92-13.81]	5.58 [0.73-11.25]	2.29 [2.09 to 2.49]	2.29
Oman	0.22 [0.03-0.44]	3.35 [0.46-6.76]	0.81 [0.14-1.59]	5.03 [0.82-10.14]	1.94 [1.68 to 2.2]	1.94
Bhutan	0.02 [0-0.05]	0.99 [0.14-2.06]	0.07 [0.01-0.16]	1.26 [0.19-2.72]	0.79 [0.7 to 0.89]	0.79
Nepal	0.3 [0.03-0.62]	0.33 [0.04-0.66]	1.27 [0.15-2.75]	0.56 [0.07-1.21]	1.84 [1.63 to 2.06]	1.84
Palestine	0.65 [0.09-1.41]	8.71 [1.26-19.14]	1.58 [0.26-3.08]	8.29 [1.33-16.63]	-0.19 [-0.48 to 0.1]	-0.19
Saudi Arabia	3.07 [0.44-6.21]	5.95 [0.84-12.14]	12.77 [2.24-24.68]	7.84 [1.28-15.84]	0.66 [0.47 to 0.86]	0.66
India	11.86 [1.3-24.23]	0.3 [0.03-0.6]	73.45 [8.93-143.02]	0.67 [0.08-1.35]	2.64 [2.55 to 2.72]	2.64
Pakistan	3.2 [0.38-6.97]	0.62 [0.07-1.36]	17.31 [2.35-34.7]	1.62 [0.21-3.24]	3.21 [3.02 to 3.4]	3.21
Qatar	0.03 [0.01-0.07]	5.08 [0.8-11.45]	0.17 [0.03-0.33]	3.46 [0.56-7.38]	-1.81 [-2.56 to -1.05]	-1.81
United Arab Emirates	0.18 [0.02-0.36]	4.81 [0.68-9.85]	1.13 [0.18-2.15]	6.27 [0.97-12.61]	2.51 [1.94 to 3.09]	2.51
Syrian Arab Republic	2.32 [0.36-4.45]	4.86 [0.74-9.51]	7.96 [1.29-16.09]	7.21 [1.13-14.84]	0.73 [0.47 to 1]	0.73
Turkey	13.51 [1.85-27.05]	4.54 [0.61-8.93]	32.44 [5-67.79]	3.81 [0.58-8.04]	-0.61 [-0.95 to -0.28]	-0.61
Bangladesh	1.47 [0.17-3.11]	0.35 [0.04-0.73]	10.61 [1.39-22.85]	0.84 [0.11-1.81]	3.18 [3.01 to 3.36]	3.18
Comoros	0.02 [0-0.03]	0.98 [0.12-1.96]	0.07 [0.01-0.14]	1.63 [0.23-3.31]	1.38 [1.14 to 1.61]	1.38
Central African Republic	0.07 [0.01-0.15]	0.76 [0.08-1.64]	0.27 [0.03-0.6]	1.53 [0.19-3.52]	2.21 [2.16 to 2.27]	2.21
Angola	0.22 [0.03-0.43]	0.66 [0.08-1.34]	1.43 [0.2-2.93]	1.53 [0.21-3.12]	2.38 [2.22 to 2.54]	2.38
Democratic Republic of the Congo	1.04 [0.11-2.18]	0.82 [0.09-1.76]	5.21 [0.65-11.44]	1.93 [0.24-4.23]	2.67 [2.59 to 2.76]	2.67
Gabon	0.1 [0.01-0.2]	1.97 [0.26-3.89]	0.29 [0.04-0.56]	3.47 [0.47-6.99]	1.66 [1.45 to 1.87]	1.66
Equatorial Guinea	0.02 [0-0.05]	1.4 [0.18-2.94]	0.12 [0.02-0.24]	2.95 [0.39-6.01]	2.17 [1.9 to 2.45]	2.17
Congo	0.12 [0.01-0.26]	1.23 [0.13-2.64]	0.58 [0.08-1.16]	2.64 [0.33-5.66]	2.13 [1.97 to 2.3]	2.13
Kenya	0.32 [0.04-0.65]	0.46 [0.06-0.94]	2.09 [0.27-4.15]	1.13 [0.14-2.35]	3.24 [3.11 to 3.37]	3.24
Burundi	0.15 [0.02-0.32]	0.69 [0.08-1.52]	0.25 [0.03-0.52]	0.65 [0.08-1.36]	-0.89 [-1.23 to -0.55]	-0.89
Eritrea	0.03 [0-0.07]	0.4 [0.04-0.87]	0.14 [0.02-0.32]	0.67 [0.08-1.46]	1.73 [1.55 to 1.9]	1.73
Malawi	0.19 [0.02-0.36]	0.63 [0.07-1.31]	0.95 [0.12-1.91]	1.57 [0.2-3.24]	2.7 [2.48 to 2.92]	2.7
Ethiopia	0.55 [0.06-1.19]	0.31 [0.03-0.67]	1.48 [0.19-3.01]	0.39 [0.05-0.8]	0.34 [0.17 to 0.5]	0.34
Djibouti	0 [0-0.01]	0.37 [0.04-0.84]	0.03 [0-0.07]	0.68 [0.08-1.42]	1.81 [1.73 to 1.89]	1.81
Madagascar	0.34 [0.04-0.66]	0.79 [0.1-1.63]	1.34 [0.18-2.71]	1.6 [0.22-3.45]	2.14 [2.02 to 2.25]	2.14
Mauritius	0.2 [0.03-0.39]	2.89 [0.4-5.55]	0.3 [0.04-0.59]	1.67 [0.24-3.34]	-3.11 [-3.73 to -2.48]	-3.11
United Republic of Tanzania	0.65 [0.08-1.32]	0.72 [0.09-1.5]	3.95 [0.52-8.51]	1.91 [0.25-4.13]	3.22 [3.01 to 3.43]	3.22
Mozambique	0.4 [0.05-0.82]	0.75 [0.09-1.59]	2.1 [0.28-4.35]	2.05 [0.26-4.31]	3.8 [3.6 to 3.99]	3.8
Seychelles	0.01 [0-0.02]	1.79 [0.25-3.44]	0.03 [0-0.05]	2.44 [0.36-4.81]	1.33 [1.14 to 1.53]	1.33
Somalia	0.11 [0.01-0.24]	0.51 [0.05-1.13]	0.36 [0.04-0.84]	0.7 [0.08-1.59]	0.97 [0.87 to 1.08]	0.97
Uganda	0.26 [0.03-0.57]	0.46 [0.05-1.01]	0.89 [0.12-1.88]	0.72 [0.1-1.55]	0.66 [0.35 to 0.98]	0.66
Rwanda	0.21 [0.03-0.42]	0.86 [0.1-1.7]	0.41 [0.05-0.88]	0.82 [0.11-1.78]	-1.2 [-1.69 to -0.71]	-1.2
Zambia	0.16 [0.02-0.34]	0.67 [0.07-1.42]	1.12 [0.15-2.34]	2.01 [0.26-4.3]	3.43 [3.29 to 3.57]	3.43
Zimbabwe	0.31 [0.04-0.59]	0.91 [0.13-1.82]	1.88 [0.26-3.74]	3.32 [0.45-6.75]	5.11 [4.38 to 5.83]	5.11
Lesotho	0.16 [0.02-0.34]	2.22 [0.33-4.93]	0.5 [0.08-1.03]	5.79 [0.84-12.04]	4.23 [3.62 to 4.85]	4.23
South Africa	4.27 [0.61-8.5]	2.3 [0.33-4.75]	17.31 [2.55-34.39]	4.47 [0.66-8.89]	2.25 [1.68 to 2.83]	2.25
Botswana	0.1 [0.01-0.2]	2.22 [0.29-4.64]	0.37 [0.05-0.75]	3.42 [0.45-6.97]	1.75 [1.38 to 2.12]	1.75
Namibia	0.12 [0.02-0.26]	2.48 [0.33-5.49]	0.5 [0.07-0.97]	4.84 [0.67-9.91]	1.98 [1.65 to 2.32]	1.98
Eswatini	0.09 [0.01-0.17]	4.39 [0.54-9.21]	0.3 [0.04-0.63]	7.62 [0.98-16.3]	2.27 [1.78 to 2.76]	2.27
Benin	0.33 [0.04-0.68]	1.75 [0.23-3.59]	1.17 [0.16-2.43]	2.62 [0.37-5.4]	1.32 [1.15 to 1.49]	1.32
Chad	0.27 [0.03-0.59]	1.04 [0.13-2.38]	0.88 [0.11-1.91]	1.79 [0.23-3.86]	1.72 [1.59 to 1.86]	1.72
Cameroon	0.83 [0.1-1.69]	2.24 [0.28-4.61]	4.73 [0.68-9.88]	4.65 [0.66-10.03]	2.49 [2.01 to 2.96]	2.49
Gambia	0.06 [0.01-0.12]	1.82 [0.23-3.72]	0.3 [0.04-0.6]	3.38 [0.43-6.87]	1.94 [1.79 to 2.09]	1.94
Côte d'Ivoire	0.63 [0.08-1.23]	1.88 [0.23-3.7]	3.02 [0.39-6.16]	3.16 [0.41-6.36]	1.67 [1.47 to 1.87]	1.67
Cabo Verde	0.02 [0-0.05]	1 [0.13-2.06]	0.12 [0.02-0.23]	2.74 [0.43-5.58]	3.07 [2.73 to 3.42]	3.07
Burkina Faso	0.15 [0.02-0.31]	0.37 [0.05-0.78]	0.52 [0.06-1.07]	0.62 [0.08-1.29]	1.87 [1.79 to 1.95]	1.87
Ghana	0.91 [0.12-1.78]	1.6 [0.2-3.27]	6.14 [0.81-12.16]	4.35 [0.56-9.04]	3.49 [3.28 to 3.7]	3.49
Guinea-Bissau	0.06 [0.01-0.14]	1.79 [0.23-3.75]	0.2 [0.03-0.41]	3.25 [0.45-6.48]	2.11 [2.06 to 2.15]	2.11
Niger	0.18 [0.02-0.42]	0.82 [0.09-1.9]	0.7 [0.08-1.6]	1.04 [0.12-2.45]	0.84 [0.74 to 0.93]	0.84
Mali	0.31 [0.04-0.69]	0.93 [0.11-2]	0.88 [0.11-1.83]	1.15 [0.14-2.41]	0.8 [0.6 to 1]	0.8
Guinea	0.36 [0.04-0.72]	1.16 [0.14-2.4]	1.1 [0.15-2.22]	2.16 [0.28-4.51]	2.37 [2.26 to 2.49]	2.37
Liberia	0.25 [0.03-0.5]	2.28 [0.3-4.58]	0.73 [0.11-1.48]	3.94 [0.56-8.01]	1.7 [1.6 to 1.8]	1.7
Mauritania	0.33 [0.05-0.71]	3.78 [0.52-7.91]	0.87 [0.12-1.87]	4.8 [0.64-10.46]	0.58 [0.4 to 0.75]	0.58
Sao Tome and Principe	0.01 [0-0.02]	1.85 [0.24-3.81]	0.04 [0.01-0.07]	3.83 [0.52-7.61]	2.36 [2.28 to 2.43]	2.36
Nigeria	6.29 [0.8-13.35]	1.68 [0.22-3.61]	19.7 [2.73-38.97]	2.76 [0.37-5.57]	1.41 [1.24 to 1.57]	1.41
Sierra Leone	0.26 [0.03-0.52]	1.4 [0.17-2.88]	0.82 [0.1-1.67]	2.42 [0.3-5.01]	2.02 [1.9 to 2.14]	2.02
American Samoa	0.01 [0-0.01]	3.06 [0.48-6]	0.01 [0-0.02]	3.15 [0.5-6.19]	-0.21 [-0.38 to -0.04]	-0.21
Senegal	0.54 [0.07-1.04]	1.84 [0.24-3.65]	1.8 [0.25-3.57]	2.67 [0.37-5.33]	1.13 [1.05 to 1.21]	1.13
Togo	0.17 [0.02-0.34]	1.58 [0.2-3.29]	0.93 [0.12-1.86]	3.05 [0.38-6.15]	2.04 [1.95 to 2.14]	2.04
Bermuda	0.01 [0-0.03]	2.57 [0.37-5.42]	0.02 [0-0.05]	1.57 [0.24-3.24]	-1.74 [-1.96 to -1.51]	-1.74
Guam	0.01 [0-0.03]	2.21 [0.3-4.55]	0.02 [0-0.05]	1.06 [0.17-2.16]	-2.16 [-2.57 to -1.75]	-2.16
Greenland	0.01 [0-0.02]	5.46 [0.77-11.75]	0.01 [0-0.02]	2.11 [0.32-4.55]	-3.33 [-3.52 to -3.14]	-3.33
Cook Islands	0 [0-0.01]	2.76 [0.39-5.63]	0 [0-0.01]	1.96 [0.31-4]	-1.19 [-1.38 to -1]	-1.19
Monaco	0.03 [0-0.06]	3.77 [0.49-7.82]	0.02 [0-0.05]	1.84 [0.27-3.8]	-2.45 [-2.63 to -2.27]	-2.45
Niue	0 [0-0]	2.85 [0.38-5.96]	0 [0-0]	3.61 [0.53-7.31]	0.5 [0.41 to 0.59]	0.5
Nauru	0 [0-0]	5.71 [0.88-10.91]	0 [0-0.01]	7.53 [1.12-14.9]	0.69 [0.31 to 1.08]	0.69
Northern Mariana Islands	0 [0-0.01]	2.89 [0.44-5.91]	0.01 [0-0.02]	2.78 [0.43-5.6]	-0.55 [-0.81 to -0.29]	-0.55
Saint Kitts and Nevis	0.02 [0-0.04]	5.03 [0.66-10.24]	0.03 [0-0.05]	4.56 [0.67-9.26]	-0.21 [-0.39 to -0.04]	-0.21
Puerto Rico	0.52 [0.08-1.07]	1.55 [0.23-3.23]	0.62 [0.1-1.31]	0.71 [0.11-1.49]	-3.02 [-3.28 to -2.75]	-3.02
Palau	0 [0-0.01]	4.25 [0.63-8.5]	0.01 [0-0.02]	4.85 [0.75-10.1]	0.54 [0.46 to 0.62]	0.54
San Marino	0.01 [0-0.02]	2.11 [0.3-4.58]	0.01 [0-0.02]	0.84 [0.11-1.99]	-2.47 [-2.84 to -2.1]	-2.47
United States Virgin Islands	0.02 [0-0.04]	2.74 [0.42-5.96]	0.03 [0-0.05]	1.46 [0.23-3.04]	-1.92 [-2.09 to -1.76]	-1.92
Tuvalu	0 [0-0]	3.18 [0.44-6.53]	0 [0-0.01]	4.11 [0.61-8.39]	0.82 [0.76 to 0.89]	0.82
Tokelau	0 [0-0]	2.96 [0.45-6.46]	0 [0-0]	3.26 [0.54-6.83]	0.17 [0.08 to 0.26]	0.17
South Sudan	0.08 [0.01-0.16]	0.33 [0.04-0.7]	0.14 [0.02-0.29]	0.44 [0.05-0.93]	0.74 [0.37 to 1.11]	0.74
Sudan	3.95 [0.53-8.44]	4.55 [0.59-9.83]	11.24 [1.67-23.08]	6.31 [0.93-13.05]	0.91 [0.86 to 0.96]	0.91
Latin America & Caribbean - WB	57.15 [8.38-113.06]	2.38 [0.35-4.7]	110.22 [16.43-227.63]	1.6 [0.24-3.32]	-1.3 [-1.42 to -1.18]	-1.3
East Asia & Pacific - WB	57.22 [7.65-114.94]	0.53 [0.07-1.08]	551.03 [75.96-1115.3]	1.7 [0.23-3.45]	2.3 [1.91 to 2.68]	2.3
Middle East & North Africa - WB	61.48 [9.37-122.3]	5.73 [0.86-11.73]	210.02 [34.33-406.65]	6.94 [1.09-13.87]	0.61 [0.57 to 0.65]	0.61
Eastern Africa	6.74 [0.92-13.28]	1.12 [0.15-2.22]	22.46 [3.27-46.03]	1.67 [0.24-3.46]	1.05 [0.97 to 1.13]	1.05
Europe & Central Asia - WB	356.77 [51.58-725.46]	3.41 [0.49-6.98]	544.52 [80.29-1137.64]	3.05 [0.45-6.25]	-2.32 [-3.14 to -1.49]	-2.32
Southern Africa	6.01 [0.87-11.78]	1.68 [0.24-3.43]	26.45 [3.79-52.66]	3.56 [0.51-7.19]	2.56 [2.08 to 3.04]	2.56
South Asia - WB	20.57 [2.37-41.46]	0.4 [0.05-0.78]	112.04 [14.37-219.17]	0.81 [0.1-1.61]	2.3 [2.24 to 2.37]	2.3
Sub-Saharan Africa - WB	26.2 [3.53-50.18]	1.42 [0.19-2.81]	100.24 [14.07-193.52]	2.5 [0.35-5.03]	1.35 [0.71 to 1.99]	1.35
Northern Africa	35.55 [5.49-74.48]	6.71 [1.02-14.52]	122.17 [20.09-236.73]	9.1 [1.45-17.72]	1.24 [1.14 to 1.35]	1.24
Commonwealth High Income	39 [5.62-80]	2.5 [0.36-5.13]	27.14 [3.8-59.78]	0.85 [0.12-1.86]	-3.82 [-4 to -3.63]	-3.82
Western Africa	10.52 [1.36-21.19]	1.55 [0.2-3.2]	38.14 [5.13-75.52]	2.65 [0.35-5.21]	1.66 [1.56 to 1.75]	1.66
Commonwealth Low Income	3.78 [0.46-7.36]	0.49 [0.06-0.96]	21.82 [2.81-45.55]	1.07 [0.14-2.23]	2.63 [2.51 to 2.74]	2.63
Africa	61.43 [9.25-123.97]	2.57 [0.38-5.15]	221.58 [33.81-435.1]	4.1 [0.62-8.22]	1.63 [1.51 to 1.76]	1.63
Asia	169.92 [22.84-331.54]	0.99 [0.13-1.99]	779.8 [109.44-1524.28]	1.62 [0.23-3.21]	1.5 [1.43 to 1.57]	1.5
Commonwealth Middle Income	31.3 [3.92-59.79]	0.61 [0.08-1.17]	152.77 [20.48-293.86]	1.1 [0.15-2.14]	1.86 [1.74 to 1.99]	1.86
America	112.97 [16.43-227.23]	1.9 [0.28-3.81]	199.34 [29.1-419.43]	1.44 [0.21-3.02]	-1.24 [-1.42 to -1.05]	-1.24
African Region	25.62 [3.44-49.3]	1.4 [0.19-2.77]	103.36 [14.64-205.07]	2.57 [0.37-5.23]	1.91 [1.72 to 2.09]	1.91
South-East Asia Region	23.88 [2.86-46.05]	0.39 [0.05-0.74]	144.26 [18.6-280.14]	0.86 [0.11-1.69]	2.63 [2.54 to 2.71]	2.63
Europe	533.73 [77.35-1068.46]	5.18 [0.75-10.42]	520.01 [76.48-1091.54]	2.97 [0.44-6.1]	-2.37 [-2.66 to -2.07]	-2.37
Region of the Americas	112.97 [16.43-227.23]	1.9 [0.28-3.81]	199.34 [29.1-419.43]	1.44 [0.21-3.02]	-1.24 [-1.42 to -1.05]	-1.24
European Region	550.84 [79.92-1100.5]	5.18 [0.75-10.4]	548.97 [80.92-1146.18]	3.04 [0.45-6.24]	-2.29 [-2.59 to -2]	-2.29
Western Pacific Region	95.95 [12.46-191.3]	0.98 [0.13-1.98]	493.82 [67.81-1010.41]	1.75 [0.24-3.6]	1.9 [1.8 to 2]	1.9
Eastern Mediterranean Region	67.13 [10.38-134.66]	4.16 [0.63-8.63]	228.41 [36.84-445.93]	5.83 [0.91-11.7]	1.13 [1.08 to 1.18]	1.13
Central Africa	2.61 [0.31-4.96]	1.14 [0.14-2.23]	12.35 [1.68-24.8]	2.42 [0.32-4.86]	2.44 [2.27 to 2.6]	2.44
Basic Health System	217.87 [31.23-430.95]	1.72 [0.24-3.46]	855.83 [126.09-1701.99]	2.42 [0.35-4.87]	1.1 [1.03 to 1.18]	1.1
Minimal Health System	6.49 [0.82-12.5]	1.21 [0.15-2.36]	19.83 [2.71-39.04]	1.87 [0.25-3.79]	1.37 [1.32 to 1.41]	1.37
Advanced Health System	612.27 [88.73-1226.45]	3.76 [0.54-7.57]	648.68 [95.27-1361.94]	2.05 [0.31-4.19]	-2.54 [-2.8 to -2.27]	-2.54
Limited Health System	41.99 [5.3-81.47]	0.62 [0.08-1.2]	197.09 [26.09-381.92]	1.11 [0.15-2.19]	1.79 [1.74 to 1.84]	1.79
North America	56.38 [8.05-117]	1.53 [0.22-3.17]	89.78 [12.77-189.9]	1.24 [0.18-2.58]	-1.33 [-1.68 to -0.97]	-1.33
Central Europe, Eastern Europe, and Central Asia	383.04 [56.12-755.35]	8.68 [1.26-17.3]	411.69 [60.31-834.69]	6.2 [0.91-12.57]	-1.78 [-2.19 to -1.36]	-1.78
Southeast Asia, East Asia, and Oceania	88.42 [11.33-174.04]	0.88 [0.11-1.74]	529.02 [72.84-1067.05]	1.98 [0.27-4.02]	2.7 [2.53 to 2.87]	2.7
Latin America and Caribbean	44.81 [6.59-87.58]	2.24 [0.32-4.47]	96.87 [14.48-198.8]	1.61 [0.24-3.32]	-1.14 [-1.26 to -1.01]	-1.14
High-income	244.72 [34.51-499.04]	2.01 [0.28-4.1]	236.77 [32.92-508.11]	0.88 [0.13-1.86]	-3.01 [-3.23 to -2.79]	-3.01
Sub-Saharan Africa	22.05 [2.88-42.71]	1.28 [0.17-2.5]	88.73 [12.19-170.92]	2.34 [0.32-4.65]	1.93 [1.72 to 2.14]	1.93
League of Arab States	56.59 [8.75-113.74]	6.22 [0.94-12.8]	192.66 [31.59-370.57]	8.08 [1.28-16.28]	0.88 [0.84 to 0.93]	0.88
World Bank Regions	546.44 [78.39-1095.27]	1.66 [0.24-3.4]	1717.85 [249.78-3466.84]	2.05 [0.3-4.16]	-1.09 [-1.8 to -0.37]	-1.09
WHO region	876.39 [125.74-1755.34]	2.57 [0.37-5.16]	1718.16 [249.8-3467.23]	2.07 [0.3-4.18]	-1.09 [-1.24 to -0.95]	-1.09
European Union	225.96 [32.38-459.03]	3.72 [0.53-7.56]	181.03 [25.92-388.66]	1.58 [0.23-3.36]	-3.03 [-3.14 to -2.92]	-3.03
G20	651.86 [92.79-1306.4]	2.47 [0.35-5]	1187.34 [170.11-2391.45]	1.83 [0.26-3.69]	-1.39 [-1.56 to -1.23]	-1.39
Four World Regions	878.05 [125.97-1758.18]	2.57 [0.37-5.15]	1720.72 [250.17-3472.35]	2.06 [0.3-4.17]	-1.1 [-1.24 to -0.96]	-1.1
OECD Countries	326.75 [46.6-664.46]	2.43 [0.35-4.94]	327.79 [46.97-694.35]	1.12 [0.16-2.33]	-2.88 [-3.08 to -2.68]	-2.88
African Union	61.43 [9.25-123.97]	2.57 [0.38-5.15]	221.58 [33.81-435.1]	4.1 [0.62-8.22]	1.63 [1.51 to 1.76]	1.63
Commonwealth	74.07 [10-145.26]	1.13 [0.15-2.26]	201.73 [27.12-390.03]	1.11 [0.15-2.2]	-0.16 [-0.23 to -0.08]	-0.16
Gulf Cooperation Council	3.69 [0.54-7.38]	5.37 [0.77-10.96]	15.89 [2.8-30.06]	6.41 [1.06-13.04]	0.46 [0.21 to 0.71]	0.46
Organization of Islamic Cooperation	120.88 [18.1-243.48]	2.67 [0.4-5.46]	397.65 [61.77-781.8]	3.65 [0.55-7.35]	0.94 [0.88 to 1]	0.94
Nordic Region	8.12 [1.15-16.39]	1.99 [0.28-3.99]	5.53 [0.75-11.98]	0.78 [0.11-1.67]	-3.25 [-3.4 to -3.1]	-3.25
Sahel Region	9.77 [1.3-18.94]	1.73 [0.23-3.49]	31.43 [4.45-62.06]	2.68 [0.37-5.31]	1.3 [1.23 to 1.38]	1.3
Association of Southeast Asian Nations	12.63 [1.64-23.88]	0.56 [0.07-1.06]	72.41 [9.78-141.61]	1.23 [0.16-2.44]	2.67 [2.52 to 2.82]	2.67
Health System Grouping Levels	878.62 [126.05-1759.37]	2.57 [0.37-5.15]	1721.43 [250.28-3473.95]	2.06 [0.3-4.17]	-1.1 [-1.24 to -0.96]	-1.1

The demographic decomposition analysis quantified the contributions of three drivers to the change in global IS-HBMI deaths between 1990 and 2021. The net increase of 843.16 thousand deaths resulted from the combined effect of population growth  (contributing 930.13 thousand deaths, 83%  of the total increase), population ageing  (contributing -80.07 thousand deaths, -14%), and epidemiological transition  (i.e., changes in age-specific mortality rates, contributing -6.90 thousand deaths, -3%). The substantial negative contribution of epidemiological transition highlights the pivotal role of declining age-specific mortality rates in mitigating the burden increase driven by population growth and ageing. This finding aligns with the observed divergence of rising absolute deaths alongside falling age-standardized rates.

The global burden of IS-HBMI in 2021 showed significant sex differences. 1 The death count and age-standardized mortality rate (ASMR) were higher in males (1,006.61 thousand, 95% UI: 140.28–2,026.99; ASMR: 2.89 per 100,000) than in females (717.30 thousand, 95% UI: 99.96–1,452.13; ASMR: 1.38 per 100,000) (Table 1). From 1990 to 2021, the ASMR declined in both sexes, but the estimated annual percentage change (EAPC) was more pronounced in females (-1.34%, 95% CI: -1.50 to -1.18) than in males (-0.92%, 95% CI: -1.07 to -0.78). This pattern of a steeper decline among females was consistent across all Socio-demographic Index (SDI) quintiles (Table 1). These results suggest an asymmetry in the effectiveness of IS-HBMI risk reduction between sexs, which appears to be associated with the level of socioeconomic development.

In terms of health loss, the global number of disability-adjusted life years (DALYs) due to IS-HBMI reached 44,391.86 thousand (95% UI: 6,490.3–86,474.85) in 2021, more than doubling from 1990. The age-standardized DALY rate (ASDR) declined modestly from 55.30 to 51.52 per 100,000, with an estimated annual percentage change (EAPC) of -0.58% (95% CI: -0.71 to -0.46). Similar to the mortality pattern, the ASDR was higher in males (51.98 per 100,000) than in females (50.74 per 100,000) in 2021. However, the rate of decline in ASDR was slightly faster in females (EAPC: -0.92%) compared to males (EAPC: -0.10%). Pronounced socioeconomic disparities were also evident in ASDR trends: high-SDI regions experienced a steady decline (EAPC: -1.49%), whereas low- and low-middle-SDI regions saw an increase (EAPC: +1.47% and +1.59%, respectively) (Table 2).

**Table 2:** Disability-adjusted life year (DALY) cases and age-standardized DALY rate (ASDR) of ischemic stroke attributable to high body-mass index, by sex, Socio-demographic Index (SDI) quintile, and Global Burden of Disease (GBD) region, 1990 and 2021, with estimated annual percentage change (EAPC), 1990–2021. Note: EAPC indicates estimated annual percentage change; ASR, age-standardized rate; GBD, Global Burden of Disease; HBMI, high body mass index; SDI, sociodemographic index; and UI, uncertainty interval. Note: The unit for the number of DALYs is thousands.

IS						
	1990		2021		1990-2021	
	DALY cases	Age-standardized DALY rate per 100,000	DALY cases	Age-standardized DALY rate per 100,000	EAPC of Age-standardized DALY rate	
	No. *102 (95% UI)	No. (95% UI)	No. *102 (95% UI)	No.(95% UI)	No. (95% CI)	
Overall	409659.12 [373202.3-447262.56]	1142.8 [1036.88-1249.54]	622662.48 [557523.16-686873.1]	737.72 [658.81-814.07]	-1.62 [-1.72 to -1.53]	-1.62
Sex						
Female	212878.39 [191009.88-232370.04]	1054.94 [947.91-1153.96]	291007.93 [252706.96-327039.94]	626.2 [544.26-703.77]	-1.94 [-2.05 to -1.83]	-1.94
Male	196780.73 [176682.73-219514.65]	1241.98 [1112.91-1375.79]	331654.55 [296521.49-371633.71]	867.65 [773.77-973.12]	-1.32 [-1.4 to -1.23]	-1.32
Socio-demographic index						
High SDI	90934.13 [81682.37-98303.95]	809.83 [725.43-877.07]	76361.85 [66123.29-86401.85]	336.71 [294.05-378.32]	-3.13 [-3.27 to -2.98]	-3.13
High-middle SDI	151701.73 [138451.7-163279.63]	1683.58 [1533.42-1814.68]	186782.84 [166117.11-208475.96]	949.71 [844.64-1060.26]	-2.23 [-2.45 to -2.02]	-2.23
Middle SDI	97028.24 [86671.99-110523.91]	1088.2 [970.99-1235.33]	213136.87 [189003.45-238300.54]	850.05 [751.81-948.34]	-0.84 [-0.92 to -0.77]	-0.84
Low-middle SDI	51376.36 [45033.91-59783.57]	973.73 [851.41-1132.16]	110457.84 [97826.99-126871.53]	845.32 [749.22-963.3]	-0.49 [-0.53 to -0.45]	-0.49
Low SDI	17973.7 [15066.3-22835.11]	947.03 [795.08-1197.35]	35296.5 [30038.28-43602.2]	814.9 [691.16-1004.46]	-0.55 [-0.6 to -0.49]	-0.55
Region						
Andean Latin America	900.46 [784.65-1014.37]	472.43 [412.58-532.77]	1518.62 [1273.38-1816.64]	265.36 [222.93-318.09]	-2.08 [-2.3 to -1.87]	-2.08
Australasia	1412.3 [1245.15-1556.87]	615.3 [539.67-679.39]	1235.56 [1033.45-1424.13]	207.25 [174.99-238.54]	-3.76 [-3.92 to -3.6]	-3.76
Caribbean	1867.78 [1674.03-2066.02]	757.41 [678.72-837.05]	2990.46 [2567.59-3491.75]	553.14 [474.43-645.98]	-0.95 [-1.03 to -0.88]	-0.95
Central Asia	6379.92 [5809.17-6947.76]	1440.61 [1306.21-1568.46]	8880.36 [7879.48-9871.97]	1212.15 [1076.93-1342.4]	-1.01 [-1.29 to -0.73]	-1.01
Central Europe	29396.23 [27420.8-31176.41]	2098.81 [1941.01-2230.5]	23115.73 [20897.68-25224.19]	981.55 [889.26-1070.23]	-2.78 [-2.93 to -2.64]	-2.78
Central Latin America	4101.98 [3753.75-4382.61]	552.45 [505.53-591.88]	6873.2 [5988.26-7814.51]	286.73 [250.19-325.98]	-2.32 [-2.5 to -2.15]	-2.32
Central Sub-Saharan Africa	1882.07 [1459.23-2403.28]	1092.1 [847.56-1373.5]	3922.19 [2967.04-5120.9]	957.73 [733.52-1260.87]	-0.57 [-0.63 to -0.52]	-0.57
East Asia	92505.39 [79008.81-109374.55]	1241.74 [1063.46-1463.85]	215087.41 [182048.73-249034.07]	1033.67 [877.48-1197.02]	-0.56 [-0.75 to -0.36]	-0.56
Eastern Europe	66901.41 [61530.09-70950.36]	2527.39 [2304.51-2687.54]	50478.19 [44942.03-55528.09]	1413.05 [1260.28-1552.04]	-2.66 [-3.13 to -2.19]	-2.66
Eastern Sub-Saharan Africa	5161.51 [4206.62-6405.84]	846.42 [700.03-1034.73]	10682.56 [9008.86-12531.56]	770.12 [652.92-906.14]	-0.4 [-0.44 to -0.36]	-0.4
High-income Asia Pacific	17106.55 [15325.81-18621.36]	921.93 [819.13-1000.76]	15543.7 [12956.19-17858.56]	282.93 [241.9-325.76]	-4.18 [-4.35 to -4]	-4.18
High-income North America	17408.15 [15211.31-19454.81]	475.04 [414.32-531.38]	19869.04 [16831.53-22724.82]	291.66 [247.58-333.93]	-2.08 [-2.35 to -1.81]	-2.08
North Africa and Middle East	24912.66 [21770.77-28682.23]	1677.67 [1458.78-1923.11]	48093.03 [42072.67-54298.91]	1177.12 [1025.56-1332.17]	-1.17 [-1.19 to -1.15]	-1.17
Oceania	183.63 [144.81-233.35]	795.57 [642.49-1003.47]	403.65 [331.44-500.49]	674.25 [554.37-837.95]	-0.64 [-0.7 to -0.58]	-0.64
South Asia	35632.23 [29129.68-44815.76]	729.66 [600.16-913.25]	82839.09 [70922.9-105219.85]	619.87 [533.85-772.52]	-0.68 [-0.75 to -0.62]	-0.68
Southeast Asia	26711.64 [23723.01-29758.45]	1222.73 [1081.95-1367.31]	65488.85 [55872.05-75637.03]	1125.35 [969.2-1297.42]	-0.22 [-0.34 to -0.09]	-0.22
Southern Latin America	3640.53 [3232.09-4000.75]	832.56 [736.89-914.71]	3070.66 [2727.08-3422.03]	339.96 [301.59-379.24]	-2.65 [-2.77 to -2.53]	-2.65
Southern Sub-Saharan Africa	1985.11 [1683.03-2230.35]	820.63 [692.29-926.83]	4660.18 [4198.82-5129.04]	950.19 [854.17-1049.29]	0.6 [0.15 to 1.05]	0.6
Tropical Latin America	9694.28 [8870.78-10305.12]	1203.92 [1087.42-1289.2]	11164.79 [9897.15-12208.77]	447.28 [395.28-490.03]	-3.14 [-3.27 to -3.01]	-3.14
Western Europe	52390.26 [47097.84-56627.14]	859.33 [771.06-930.32]	27945.31 [23768.62-31661.37]	252.84 [217.1-285.22]	-4.14 [-4.31 to -3.96]	-4.14
Western Sub-Saharan Africa	9485.02 [7622.53-12352.11]	1241.51 [1000.43-1600.25]	18799.89 [15802.26-22369.19]	1128.04 [951.13-1330.93]	-0.29 [-0.4 to -0.18]	-0.29
Cambodia	447.79 [371.07-540.67]	1225.19 [1011.39-1481.38]	1093.21 [880.61-1303.56]	1111.87 [894.7-1322.28]	-0.44 [-0.54 to -0.34]	-0.44
China	89328.84 [75903.57-106128.61]	1246.77 [1063.55-1473.99]	209790.53 [177135.57-243398.86]	1046.86 [888.29-1213.95]	-0.53 [-0.73 to -0.33]	-0.53
Indonesia	10423.3 [8712.64-12195.71]	1265.27 [1052.05-1486.77]	30126.42 [22810.98-37438.99]	1556.14 [1215.79-1916.11]	0.74 [0.59 to 0.9]	0.74
Democratic People's Republic of Korea	1811.49 [1423.33-2346.69]	1283.52 [1015.11-1629.77]	3936.7 [3115.38-4976.61]	1238.64 [984.43-1574.09]	-0.15 [-0.32 to 0.02]	-0.15
Taiwan (Province of China)	1365.07 [1227.47-1492.45]	998.58 [895.16-1090.96]	1360.18 [1156.89-1572.28]	319.85 [270.82-369.47]	-3.63 [-3.84 to -3.42]	-3.63
Myanmar	3526.11 [2670.21-4374.59]	1748.04 [1354.47-2146.84]	5140.87 [4128.68-6347.24]	1201.45 [967.82-1482.66]	-1.37 [-1.46 to -1.29]	-1.37
Thailand	2511.22 [2109.52-2968.96]	798.18 [672.7-942.17]	5343.57 [4311.4-6590.54]	497.45 [400.96-613.61]	-2.02 [-2.23 to -1.81]	-2.02
Lao People's Democratic Republic	332.86 [265.42-415.24]	1859.13 [1482.44-2306.14]	497.74 [386.41-617.81]	1276.48 [1008.62-1566.83]	-1.35 [-1.42 to -1.27]	-1.35
Malaysia	852.18 [745.13-972.04]	994.26 [872.54-1144.51]	2015.62 [1773.33-2287.36]	777.07 [685.07-878.18]	-0.61 [-0.74 to -0.47]	-0.61
Philippines	2046.64 [1781.38-2262.18]	854.25 [749.27-942.56]	5840.99 [5028.49-6735.93]	791.77 [680.71-908.98]	-0.11 [-0.21 to -0.01]	-0.11
Sri Lanka	1252.18 [1120.82-1394.5]	1467.73 [1309.86-1624.27]	2345.52 [1690.62-2963.03]	970.27 [709.22-1223.17]	-0.81 [-1.06 to -0.55]	-0.81
Maldives	10.29 [9-11.83]	1404.4 [1214.33-1625.52]	17.86 [14.83-20.98]	604.07 [497.1-711.32]	-3.12 [-3.24 to -2.99]	-3.12
Viet Nam	5123.75 [4153.01-6337.6]	1402.86 [1140.47-1734.6]	12772.61 [10127.56-15369.73]	1468.22 [1178.51-1755.19]	0.45 [0.3 to 0.59]	0.45
Micronesia (Federated States of)	4.94 [3.88-6.46]	1124.91 [887.68-1456.73]	5.67 [4.41-7.45]	920.64 [715.01-1187.37]	-0.73 [-0.78 to -0.67]	-0.73
Marshall Islands	1.42 [1.12-1.84]	1020.11 [807.13-1300.25]	2.51 [1.91-3.33]	923 [716.05-1197.81]	-0.36 [-0.42 to -0.31]	-0.36
Fiji	24.99 [20.9-29.33]	827.17 [689.9-968.78]	46.49 [37.53-56.15]	740.82 [604.41-890.12]	-0.65 [-0.81 to -0.48]	-0.65
Timor-Leste	21.8 [17.67-25.81]	1027.37 [843.55-1218.29]	84.74 [62.12-109.21]	1108.31 [825.7-1433.86]	0.42 [0.26 to 0.57]	0.42
Kiribati	3.19 [2.67-3.71]	988.84 [810.36-1154.85]	6.07 [4.96-7.37]	993.17 [821.14-1182.92]	-0.03 [-0.09 to 0.03]	-0.03
Papua New Guinea	102.35 [71.68-142.42]	750.34 [540.89-1030.56]	253.59 [191.54-336.47]	658.04 [495.79-878.11]	-0.51 [-0.6 to -0.41]	-0.51
Samoa	6.47 [5.48-7.82]	886.01 [748.36-1069.65]	9.8 [8.29-11.58]	762.86 [645.39-899.44]	-0.55 [-0.62 to -0.48]	-0.55
Solomon Islands	10.2 [7.92-13.36]	977.76 [769.62-1262.88]	26.13 [19.84-34.6]	941.88 [720.97-1249.26]	-0.14 [-0.26 to -0.02]	-0.14
Tonga	2.75 [2.31-3.22]	565.76 [475.19-660.46]	4.04 [3.3-4.87]	525.33 [429.23-630.77]	-0.15 [-0.26 to -0.05]	-0.15
Armenia	301.14 [269.88-329.63]	1225.55 [1092.24-1341.63]	367.77 [327.2-412.29]	851.13 [758.02-955.15]	-2.06 [-2.39 to -1.73]	-2.06
Uzbekistan	1347.53 [1181.96-1496.42]	1193.98 [1051.27-1324.8]	2670.41 [2300.05-3076.76]	1124.13 [971.9-1289.01]	-0.76 [-1.1 to -0.43]	-0.76
Vanuatu	6.01 [4.81-7.53]	1189.3 [949.67-1468.66]	14.38 [11.78-17.64]	998.56 [803.04-1198.03]	-0.71 [-0.77 to -0.66]	-0.71
Azerbaijan	376.28 [302.46-458.65]	815.98 [657.03-989.76]	601.74 [477.98-749.63]	688.24 [552.16-852.09]	-0.42 [-0.66 to -0.17]	-0.42
Kazakhstan	2431.95 [2140.88-2709.73]	2066.69 [1809.02-2296.49]	2458.21 [2111.2-2789.79]	1586.04 [1376.55-1793.88]	-1.43 [-1.92 to -0.93]	-1.43
Kyrgyzstan	528.18 [469.86-585.45]	1889.05 [1682.74-2097.12]	530.36 [442.76-621.31]	1153.96 [967.02-1338.14]	-2.1 [-2.47 to -1.73]	-2.1
Georgia	703.32 [572.86-815.86]	1194.7 [976.26-1378.23]	926.26 [819.46-1042.08]	1474.97 [1302.76-1658.74]	0.63 [0.11 to 1.14]	0.63
Mongolia	49.08 [39.79-58.72]	491.81 [395.33-595.26]	105.26 [84.12-129.11]	498.35 [390.2-616.97]	-0.01 [-0.36 to 0.34]	-0.01
Tajikistan	382.81 [315.7-454.95]	1481.35 [1216.36-1756.82]	569.68 [459.37-695.09]	1195.54 [967.81-1447.7]	-0.96 [-1.31 to -0.6]	-0.96
Turkmenistan	259.62 [211.19-304.05]	1418.73 [1164.16-1654.03]	650.67 [499.63-823.27]	1728.33 [1346.53-2157.35]	0.43 [-0.02 to 0.89]	0.43
Bosnia and Herzegovina	730.81 [638.7-835.43]	2065.36 [1817.7-2345.38]	913.6 [744.35-1071.69]	1426.09 [1163.34-1676.98]	-1.48 [-1.6 to -1.35]	-1.48
Bulgaria	2699.1 [2482.39-2886.75]	2557.94 [2355.25-2749.33]	3139.09 [2722.65-3590.49]	2121.98 [1842.59-2422.21]	-0.45 [-0.61 to -0.28]	-0.45
Croatia	1078.43 [986.95-1159.57]	2023.46 [1844.39-2173.74]	689.8 [603.69-778.93]	694.22 [610.41-782.92]	-3.6 [-3.73 to -3.47]	-3.6
Albania	147.42 [123.54-175.76]	838.09 [698.43-997.35]	261.29 [203.02-338.39]	614.13 [480.56-792.5]	-0.66 [-0.91 to -0.42]	-0.66
Czechia	3199.21 [2918.63-3481.83]	2311.49 [2112.26-2515.35]	1146.66 [1003.97-1302.14]	501.34 [436.96-569.3]	-5.26 [-5.57 to -4.95]	-5.26
Hungary	3152.62 [2914.14-3345.37]	2198.06 [2026.18-2334.45]	1505.91 [1296.46-1705.09]	733.74 [631.5-832.21]	-3.93 [-4.13 to -3.73]	-3.93
North Macedonia	496.34 [446.1-550.94]	3028.43 [2723.33-3349.53]	737.48 [603.19-881.92]	2729.63 [2262.63-3226.99]	-0.64 [-1.06 to -0.23]	-0.64
Belarus	2302.53 [2066.78-2507.38]	1795.46 [1612.8-1952.38]	2020.95 [1689.51-2368.81]	1234.95 [1028.41-1447.71]	-1.94 [-2.39 to -1.49]	-1.94
Montenegro	35.97 [29.8-42.97]	618.48 [512.82-738.81]	75.43 [60.36-92.22]	842.53 [673.71-1022.7]	1.22 [1.12 to 1.32]	1.22
Romania	5543.71 [4994.88-6060.48]	2261.22 [2041.07-2464.38]	5301.51 [4636.46-5932.32]	1300.79 [1143.21-1454.91]	-2.33 [-2.6 to -2.06]	-2.33
Poland	7673.37 [7125.17-8133.43]	1828.51 [1689.79-1943.47]	4904.5 [4324.98-5484.88]	649.18 [574.84-726.25]	-3.72 [-3.84 to -3.6]	-3.72
Serbia	2866.58 [2509.59-3208.83]	3289.64 [2905.31-3671.16]	3118.72 [2638.31-3625.77]	1780.77 [1506.66-2074.92]	-2.56 [-2.82 to -2.3]	-2.56
Slovakia	984.13 [872.17-1120.29]	1666.38 [1478.19-1895.53]	799.04 [671.98-939.46]	825.44 [694.5-967.03]	-2.35 [-2.45 to -2.25]	-2.35
Slovenia	318.42 [290.88-343.68]	1295.02 [1180.48-1399.48]	186.23 [157.76-213.04]	365.07 [310.65-417.2]	-4.13 [-4.35 to -3.9]	-4.13
Estonia	433.11 [388.8-467.44]	2120.61 [1903.83-2288.26]	131.68 [112.63-151.04]	454.79 [391.28-519.13]	-6.34 [-6.89 to -5.78]	-6.34
Latvia	810.17 [737.88-867.66]	2262.16 [2058.45-2423.96]	578.15 [499.24-652.95]	1297.59 [1125.59-1469.2]	-2.28 [-2.54 to -2.02]	-2.28
Lithuania	599.79 [544.03-647.41]	1330.4 [1206.05-1441]	538.06 [464.33-608.41]	863.24 [753.33-976.88]	-1.32 [-1.65 to -0.99]	-1.32
Republic of Moldova	479.97 [423.23-529.36]	1281.06 [1143.43-1406.34]	574.88 [516.51-645.78]	948.68 [852.31-1063.86]	-0.75 [-1.18 to -0.32]	-0.75
Ukraine	16147.64 [14730.58-17323.12]	2340.7 [2133.03-2509.5]	10258.51 [8215.06-12657.57]	1297.39 [1037.04-1592.42]	-2.56 [-2.86 to -2.27]	-2.56
Russian Federation	46128.21 [42271.76-48926.18]	2742.41 [2492.1-2921.61]	36375.96 [32414.62-39813.93]	1507.07 [1344.91-1648.16]	-2.79 [-3.34 to -2.24]	-2.79
Brunei Darussalam	10.66 [9.13-12.4]	1226.13 [1051.45-1417.48]	14.94 [12.58-17.45]	565.07 [474.77-660.67]	-2.38 [-2.61 to -2.16]	-2.38
Republic of Korea	4100.13 [3602.07-4606.76]	1745.59 [1527.32-1947.76]	3628.26 [3036.88-4227.16]	392.22 [328.05-458.28]	-5.53 [-5.8 to -5.25]	-5.53
Japan	12798.95 [11451-13949.66]	802.62 [712.85-875.34]	11762.51 [9733.09-13607.5]	261.61 [223.98-303.55]	-3.88 [-4.05 to -3.71]	-3.88
Singapore	196.81 [177.39-215.74]	999.41 [899.49-1095.93]	138 [112.41-163.31]	164.14 [133.5-194.68]	-5.95 [-6.13 to -5.76]	-5.95
Australia	1162.57 [1021.14-1287.07]	610.92 [534.56-677.05]	1004.18 [840.7-1160.45]	199.03 [167.99-228.93]	-3.87 [-4.04 to -3.71]	-3.87
New Zealand	249.74 [220.32-274.52]	636.92 [557.97-699.96]	231.39 [194.83-267.08]	253.8 [213.65-293.1]	-3.17 [-3.34 to -2.99]	-3.17
Andorra	2.11 [1.6-2.77]	414.63 [314.1-537.77]	3.4 [2.59-4.31]	204.21 [157.95-257.05]	-2.17 [-2.38 to -1.97]	-2.17
Austria	1135.36 [993.67-1239.04]	898.11 [785-981.09]	514.23 [439.91-594.6]	247.89 [213.18-287.48]	-4.35 [-4.68 to -4.03]	-4.35
Belgium	1207.26 [1059.12-1321.99]	754.06 [662.75-826.39]	643.62 [544.99-730.54]	235.79 [203.27-267.55]	-3.67 [-3.84 to -3.5]	-3.67
Cyprus	73.24 [61.3-86.34]	1359.73 [1138.33-1602.5]	61.48 [50.42-72.9]	349.61 [287.4-414.5]	-4.71 [-4.98 to -4.43]	-4.71
Finland	679.45 [608.67-742.65]	925.27 [828.64-1010.93]	448.36 [379.56-509.52]	304.32 [262.46-344.38]	-3.57 [-3.7 to -3.44]	-3.57
France	5079.71 [4463.18-5562.82]	565.59 [496.98-619.7]	3591.12 [3030.23-4096.07]	208.71 [179.28-237.97]	-3.16 [-3.3 to -3.03]	-3.16
Denmark	647.9 [586.26-707.31]	737.58 [667.91-806.62]	361.38 [312.73-407.22]	271.53 [236.25-306.5]	-3.58 [-3.77 to -3.39]	-3.58
Germany	13394.66 [11958.77-14593.55]	991.62 [885.53-1079.5]	7003.66 [6031.82-7909.73]	318.69 [277.98-361.2]	-3.73 [-4 to -3.46]	-3.73
Greece	1876.75 [1668.38-2027.76]	1264.73 [1123.38-1368.74]	1171.54 [992.27-1333.13]	369.55 [317.39-416.18]	-4.67 [-4.99 to -4.35]	-4.67
Netherlands	1415.72 [1244.55-1557.79]	683.55 [599.73-751.91]	1108.7 [952.79-1252.05]	286.63 [247.37-323.11]	-3.34 [-3.62 to -3.06]	-3.34
Ireland	347.16 [312.59-375.23]	847.27 [762.23-918.3]	161.02 [134.68-181.95]	191.03 [160.11-215.72]	-4.96 [-5.19 to -4.74]	-4.96
Israel	256.57 [227.8-286.25]	540.8 [479.24-603.69]	253.48 [216.69-288.75]	189.39 [161.83-216.47]	-3.81 [-3.98 to -3.65]	-3.81
Iceland	17.44 [15.27-19.46]	574.84 [505.12-640.24]	13.27 [11.05-15.4]	202.96 [171.35-235.41]	-3.47 [-3.61 to -3.32]	-3.47
Italy	7799.77 [6961.6-8468.62]	863.67 [767.23-940.09]	4419.97 [3607.72-5108.14]	235.55 [195.12-268.92]	-4.48 [-4.75 to -4.21]	-4.48
Luxembourg	65.04 [58.33-70.02]	1186.45 [1061.26-1278.08]	25.39 [21.95-28.66]	215.68 [188.6-243.73]	-5.48 [-5.64 to -5.32]	-5.48
Norway	623 [553.68-681.63]	816.3 [725.85-893.3]	270.02 [227.81-311.91]	237.34 [200.22-274.62]	-4.25 [-4.36 to -4.15]	-4.25
Malta	34.77 [30.86-37.94]	845.4 [747.52-926.18]	22.62 [18.98-25.98]	207.25 [175.75-237.84]	-4.7 [-4.9 to -4.5]	-4.7
Spain	4866.45 [4304.49-5270.57]	891.42 [788.03-968.36]	2550.55 [2142.45-2940.72]	216.41 [185.38-247.31]	-4.55 [-4.81 to -4.29]	-4.55
Portugal	2617.29 [2346.89-2842.01]	1986.89 [1769.29-2168.76]	1015.59 [852.55-1157.02]	334.26 [286.55-377.43]	-6.29 [-6.52 to -6.06]	-6.29
United Kingdom	8429.78 [7626.3-9057.33]	866.43 [783.57-932.49]	3283.97 [2806.91-3715.51]	223.16 [191.32-254.25]	-4.72 [-4.93 to -4.51]	-4.72
Sweden	1122.03 [986.32-1240.69]	663.05 [583.35-732.05]	618.63 [520.68-718.66]	242.34 [204.79-281.73]	-3.45 [-3.63 to -3.27]	-3.45
Switzerland	645.01 [567.54-711.17]	558.86 [491.86-617.19]	371.7 [309.07-425.85]	170.66 [143.5-195.74]	-3.77 [-3.92 to -3.61]	-3.77
Antigua and Barbuda	4.39 [3.76-4.89]	758.69 [648.23-845.16]	4.64 [4.07-5.09]	486.64 [425.79-536.22]	-1.65 [-1.88 to -1.42]	-1.65
Chile	817.89 [754.31-879.78]	885.45 [814.74-950.63]	914.73 [815.55-1014.79]	350.08 [312.6-388.52]	-2.63 [-2.8 to -2.46]	-2.63
Argentina	2397.87 [2090.36-2670.13]	784.73 [684.65-874.46]	1855.74 [1645.68-2082.85]	320.45 [283.84-359.83]	-2.61 [-2.79 to -2.43]	-2.61
Uruguay	424.59 [376.55-467.98]	1082.48 [956.62-1194.06]	300.01 [260.7-335.43]	482.02 [420.63-538.15]	-2.82 [-3 to -2.65]	-2.82
Canada	1579.08 [1386.67-1758.22]	489.22 [427.96-544.5]	1669.3 [1419.87-1935.28]	217.15 [183.01-251.92]	-2.95 [-3.14 to -2.76]	-2.95
United States of America	15825.31 [13823.3-17730.49]	474.08 [413.42-530.79]	18196.78 [15393.29-20804.93]	300.92 [255.35-343.84]	-1.99 [-2.27 to -1.71]	-1.99
Bahamas	8.51 [7.25-9.77]	596.31 [509.31-683.47]	14.85 [12.17-17.95]	410.11 [336.4-496.86]	-1.2 [-1.32 to -1.08]	-1.2
Barbados	28.71 [24.89-31.57]	914.91 [795.14-1005.74]	30.7 [24.96-36.95]	587.97 [478.67-707.96]	-1.61 [-1.81 to -1.41]	-1.61
Belize	3.89 [3.4-4.28]	423.37 [371-466.5]	9.77 [8.37-11.1]	366.29 [312.01-417.38]	-0.82 [-1.33 to -0.3]	-0.82
Cuba	596.86 [524.37-658.06]	600.29 [527.16-660.15]	986.45 [843.04-1129.48]	484.77 [414.55-555.7]	-0.67 [-0.77 to -0.56]	-0.67
Dominican Republic	193.67 [163.28-224.77]	590.6 [497.88-682.25]	519.01 [412.87-657.68]	531.32 [422.45-672.61]	0.03 [-0.16 to 0.21]	0.03
Grenada	10.69 [9.23-12.03]	1380.61 [1195.98-1550.48]	6.98 [5.95-7.88]	700.43 [597.36-786.79]	-2.11 [-2.3 to -1.93]	-2.11
Dominica	5.14 [4.44-5.85]	866.97 [752.4-988.34]	5.43 [4.58-6.43]	696.21 [587.3-820]	-0.71 [-0.84 to -0.59]	-0.71
Bolivia (Plurinational State of)	207.65 [149.71-275.19]	735.68 [543.34-960.62]	338.73 [237.37-470.45]	423.1 [299.73-577.05]	-1.79 [-1.94 to -1.65]	-1.79
Saint Lucia	11.82 [10.61-12.98]	1527.36 [1373.01-1675.15]	15.11 [12.45-17.99]	654.88 [539.06-780.1]	-3.3 [-3.71 to -2.89]	-3.3
Haiti	443.6 [335.05-556.77]	1686.93 [1290.63-2084.28]	743.82 [524.84-1058.58]	1283.82 [915.76-1793.13]	-0.75 [-0.82 to -0.68]	-0.75
						#VALUE!
Guyana	63.27 [56.19-70.76]	1825.04 [1622.56-2035.34]	58.52 [46.89-73.2]	1044.63 [843.21-1292.23]	-1.26 [-1.47 to -1.05]	-1.26
Saint Vincent and the Grenadines	6.96 [6.03-7.82]	1015.5 [883.48-1139.7]	8.34 [7.22-9.48]	628.62 [543.67-716.78]	-1.42 [-1.64 to -1.2]	-1.42
Jamaica	167.59 [148.23-185.58]	894.78 [792.81-989.77]	196.1 [154.48-242.57]	612.87 [481.62-756.52]	-1.01 [-1.41 to -0.6]	-1.01
Trinidad and Tobago	91.38 [81.33-99.85]	1193.22 [1058.02-1304.57]	116.36 [90.95-143.47]	619.13 [484.96-763.54]	-2.46 [-2.7 to -2.23]	-2.46
Suriname	19.74 [16.92-22.27]	851.85 [728.74-962.39]	37.23 [29.48-46.24]	619.35 [489.36-770.34]	-1.05 [-1.33 to -0.77]	-1.05
Peru	423.93 [358.42-493.4]	379.84 [319.06-443.22]	771.18 [618.99-960.1]	231.85 [185.66-288.78]	-2.1 [-2.52 to -1.68]	-2.1
Ecuador	268.88 [234.8-297.32]	539.14 [472.9-596.63]	408.71 [329.35-501.63]	263.74 [213.03-322.55]	-2.2 [-2.49 to -1.91]	-2.2
Colombia	955.08 [876.39-1032.19]	603.26 [551.13-653.7]	1296.98 [1090.6-1503.59]	234.55 [197.56-271.95]	-3.55 [-3.84 to -3.26]	-3.55
Costa Rica	67.01 [59.95-73.15]	402.62 [358.73-440.58]	130.15 [112.21-146.26]	236.1 [204.32-265.42]	-2.19 [-2.56 to -1.83]	-2.19
Honduras	119.52 [93.51-149.45]	646.94 [509.77-800.75]	428.19 [326.13-549.65]	781.03 [605.96-989.84]	0.81 [0.61 to 1.01]	0.81
El Salvador	117.6 [102.06-132.95]	409.96 [356.29-462.53]	159.45 [129.17-193.28]	246.14 [198.8-297.33]	-1.87 [-2.18 to -1.55]	-1.87
Guatemala	120.77 [105.97-135.26]	438 [385.8-489.81]	255.88 [219.77-293.51]	256.57 [220.36-293.96]	-2.25 [-2.55 to -1.94]	-2.25
Panama	75.05 [65.76-83.54]	536.55 [470.17-597.81]	143.63 [112.17-170.84]	318.47 [249.68-379.34]	-1.87 [-2.06 to -1.68]	-1.87
Venezuela (Bolivarian Republic of)	433.06 [377.47-482.77]	496.93 [433.71-554.06]	1063.85 [828.8-1339.43]	385.27 [301.42-484.35]	-1.13 [-1.41 to -0.86]	-1.13
Nicaragua	65.22 [56.71-73.6]	471.51 [408.96-533.03]	123.04 [102.79-149.76]	274.74 [229.14-334.25]	-1.7 [-1.85 to -1.54]	-1.7
Mexico	2148.68 [1969.8-2294.35]	579.8 [531.06-618.9]	3272.02 [2845.72-3710.74]	274.6 [237.71-311.51]	-2.49 [-2.63 to -2.36]	-2.49
Algeria	1463.31 [1147.8-1793.16]	1575.49 [1249.58-1928.1]	3198.39 [2491.09-4004.15]	1097.35 [866.78-1357.65]	-1.13 [-1.17 to -1.1]	-1.13
Brazil	9524.72 [8711.62-10115.17]	1213.51 [1096.57-1299.3]	10867.26 [9636.21-11872.52]	445.05 [393.75-487.02]	-3.18 [-3.32 to -3.05]	-3.18
Paraguay	169.55 [144.77-193.37]	834.62 [713.86-954.61]	297.53 [236.02-369.32]	553.05 [435.38-688.1]	-1.21 [-1.37 to -1.06]	-1.21
Egypt	5602.84 [4119.73-7791.89]	2605.24 [1971.07-3544.69]	11433.7 [8955.27-14453.53]	2212.18 [1773.97-2712.14]	-0.12 [-0.3 to 0.06]	-0.12
Bahrain	17.79 [15.66-19.97]	1423.41 [1250.61-1588.27]	47.3 [39.18-55.15]	800.91 [677.17-932.05]	-2.38 [-2.81 to -1.96]	-2.38
Iran (Islamic Republic of)	3471.63 [3091.17-3875.28]	1549.45 [1368.01-1729.5]	5987.05 [5315.34-6591.62]	829.37 [733.13-912.4]	-2.14 [-2.25 to -2.02]	-2.14
Iraq	1729.93 [1451.67-2041.67]	2251.94 [1893.93-2651.91]	4077.42 [3229.38-4957.91]	1993.65 [1602.67-2370.58]	-0.91 [-1.08 to -0.73]	-0.91
Jordan	206.81 [172.42-246.42]	1791.8 [1487.41-2125.48]	566.62 [461.9-674.44]	896.92 [736.83-1058.78]	-2.72 [-3.05 to -2.39]	-2.72
Lebanon	223.52 [177.23-288.49]	1151.4 [917.91-1486.47]	297.31 [250.68-345.18]	467.4 [393.77-543.85]	-3.03 [-3.3 to -2.76]	-3.03
Kuwait	38.78 [34.63-43.01]	676.14 [609.67-748.33]	133.04 [110.17-154.56]	473.51 [392.94-553.68]	-0.92 [-1.89 to 0.05]	-0.92
Libya	154.48 [119.79-202.69]	864.02 [666.24-1140.1]	492.45 [368.16-657.51]	993.99 [745.15-1315.43]	0.85 [0.65 to 1.05]	0.85
Morocco	2146.03 [1627.3-2790.33]	1599.71 [1212.86-2076.24]	4698.79 [3584.72-5995.46]	1484.9 [1141.07-1876.61]	-0.14 [-0.2 to -0.09]	-0.14
Palestine	153.07 [122.58-188.6]	1994.9 [1608.06-2440.91]	249.23 [213.25-286.42]	1219.22 [1038.93-1408.91]	-1.65 [-1.92 to -1.37]	-1.65
Syrian Arab Republic	660.15 [534.83-800.71]	1343.31 [1090.1-1624.58]	1180.54 [922.59-1517.9]	1033.48 [821.29-1312.69]	-1.33 [-1.54 to -1.11]	-1.33
Oman	85.24 [64.65-107.83]	1336.58 [986.85-1685.32]	162.91 [134.59-191.7]	919.88 [753.82-1088.09]	-0.67 [-0.9 to -0.43]	-0.67
Qatar	10.02 [8.4-11.59]	1324.86 [1122.89-1541.38]	39.52 [31.73-46.94]	543.3 [435.97-649.41]	-3.29 [-3.97 to -2.6]	-3.29
Tunisia	498.18 [390.7-629.81]	1154.6 [910.74-1459.05]	1089.85 [767.83-1456.19]	880.64 [624.3-1170.58]	-1.06 [-1.17 to -0.94]	-1.06
Saudi Arabia	805.28 [619.46-1012.77]	1536.4 [1192.79-1917.66]	1977.47 [1590.05-2433.97]	1119.4 [928.2-1347.99]	-1.2 [-1.35 to -1.05]	-1.2
Turkey	3942.57 [3329.01-4599.82]	1287.99 [1087.84-1498.26]	5825.94 [4806.48-7014.31]	675.11 [559.19-813.29]	-2.1 [-2.33 to -1.87]	-2.1
United Arab Emirates	62.83 [49.86-77.59]	1604.71 [1277.67-1973.04]	256.74 [209.23-309.27]	1007.05 [818.71-1212.29]	-0.01 [-0.52 to 0.5]	-0.01
Yemen	815.59 [595.34-1117.24]	1901.93 [1397.81-2590.53]	2168.22 [1535.57-2934.26]	1726.43 [1237.94-2333.15]	-0.5 [-0.58 to -0.42]	-0.5
Afghanistan	1276.15 [899.74-1757.6]	2031.24 [1456.84-2769.97]	1646.52 [1227.21-2283.81]	1865.36 [1403.16-2506.97]	-0.4 [-0.56 to -0.23]	-0.4
Nepal	690.85 [495.86-920.41]	883.23 [636.13-1153.22]	1312.06 [979.46-1798.21]	634.74 [475.96-854.29]	-1.12 [-1.25 to -0.99]	-1.12
Bangladesh	4966.34 [3940.99-6459.23]	1198.29 [954.8-1539.55]	12671.05 [9642.06-16483.4]	1038.5 [799.79-1341.17]	-0.48 [-0.71 to -0.26]	-0.48
Pakistan	4103.72 [3141.54-5342.82]	804.94 [616.71-1041.06]	8593.18 [6928.29-10982.27]	822.8 [662.4-1043.31]	-0.19 [-0.34 to -0.03]	-0.19
Bhutan	14.74 [10.31-19.51]	748.18 [511.62-1000.93]	33.14 [25.67-41.07]	585.07 [450.9-723.13]	-0.86 [-0.93 to -0.79]	-0.86
India	25856.58 [21075.77-32921.46]	653.56 [533.42-820.58]	60229.66 [50925.82-77548.47]	548.92 [465.21-698.55]	-0.72 [-0.82 to -0.62]	-0.72
Central African Republic	111.91 [80.78-148.87]	1311.26 [958.14-1733.02]	181.05 [130.21-246.8]	1166.84 [839.93-1591.57]	-0.47 [-0.53 to -0.4]	-0.47
Congo	120.18 [93.5-151.48]	1407 [1104.32-1731.84]	234.06 [185.63-287.49]	1133.11 [898.99-1400.83]	-0.95 [-1.06 to -0.84]	-0.95
Democratic Republic of the Congo	1245.8 [924.36-1634]	1046.41 [785.32-1369.06]	2498.68 [1800.54-3487.14]	917.74 [650.43-1300.99]	-0.54 [-0.59 to -0.5]	-0.54
Equatorial Guinea	19.03 [14.19-25.09]	1223.91 [925.83-1595.16]	36.56 [26.49-50.31]	915.64 [662.71-1251.33]	-1.22 [-1.46 to -0.98]	-1.22
Gabon	51.75 [41.43-63.82]	1025.71 [821.32-1255.41]	76.92 [60.41-98]	922.78 [728.97-1157.36]	-0.46 [-0.59 to -0.32]	-0.46
Angola	333.41 [262.4-416.6]	1127.78 [894.57-1399.95]	894.93 [714.59-1116.78]	1014.57 [822.1-1248.8]	-0.59 [-0.68 to -0.5]	-0.59
Burundi	288.5 [212.62-385.08]	1395.18 [1035.63-1844.54]	328.73 [251.54-432.97]	855.69 [655.96-1122.27]	-2.08 [-2.31 to -1.86]	-2.08
Comoros	17.85 [13.75-22.75]	1100.39 [856.28-1365.07]	33.51 [26.64-42]	800.62 [635.2-1007.04]	-1.3 [-1.47 to -1.12]	-1.3
Djibouti	9.3 [6.84-12.89]	920.97 [695.42-1268.66]	43.52 [32.8-58.43]	894.39 [689.58-1172.51]	-0.19 [-0.23 to -0.15]	-0.19
Eritrea	81.45 [57.22-114.41]	1021.26 [719.6-1401.08]	180.78 [136.21-237.78]	884.74 [667.84-1145.7]	-0.5 [-0.56 to -0.43]	-0.5
Rwanda	324.53 [253.07-408.55]	1403.07 [1102.07-1761.21]	371.36 [275.93-473.72]	761.61 [561.28-985.73]	-2.79 [-3.16 to -2.42]	-2.79
Kenya	424.38 [338.63-528.7]	612.21 [480.32-766.93]	1190.78 [942.45-1451.28]	667.37 [517.83-828.16]	0.46 [0.36 to 0.55]	0.46
Ethiopia	917.24 [629.9-1386.12]	569.81 [402.47-825.72]	1644.45 [1315.86-2111.86]	440.15 [348.51-567.71]	-1.07 [-1.16 to -0.98]	-1.07
Madagascar	542.09 [445.09-644.93]	1252.37 [1036.58-1481.22]	1019.99 [775.27-1312.16]	1185.46 [894.92-1509.79]	-0.25 [-0.3 to -0.2]	-0.25
Mauritius	118.79 [106.66-128.43]	1757.18 [1573.21-1904.6]	110.61 [99.41-120.87]	634.12 [565.11-693.17]	-4.39 [-4.91 to -3.87]	-4.39
Malawi	303.92 [246.15-362.83]	974.05 [795.76-1146.43]	658.65 [529.17-826.64]	1093.01 [890.35-1348.52]	0.16 [-0.06 to 0.38]	0.16
Mozambique	613.32 [509.59-737.44]	1220.28 [1025.47-1460.34]	1463.35 [1087.81-1852.2]	1557.38 [1174.72-1964.11]	1.21 [1.04 to 1.38]	1.21
Seychelles	6.09 [5.13-7.1]	1074.77 [908.15-1251.85]	7.72 [6.33-8.97]	726.31 [597.87-843.42]	-1.01 [-1.18 to -0.83]	-1.01
United Republic of Tanzania	630 [496.73-807.97]	694.65 [554.93-899.21]	1837.42 [1401.97-2371.66]	861.6 [662.69-1111.06]	0.64 [0.48 to 0.81]	0.64
Somalia	155.18 [105.11-225.06]	862.47 [594.27-1234.82]	333.9 [231.54-488.19]	725.38 [507.28-1034.34]	-0.55 [-0.58 to -0.52]	-0.55
Uganda	463.91 [366.37-586.24]	867.98 [676.13-1094.22]	795.95 [633.62-1002.18]	669.9 [542.07-839.15]	-1.43 [-1.7 to -1.15]	-1.43
Zambia	198.28 [151.83-266.03]	873.4 [671.61-1160.27]	547.67 [419.45-720.04]	1018.97 [782.42-1317.19]	0.39 [0.3 to 0.49]	0.39
Botswana	58.81 [45.49-72.33]	1340.35 [1039.2-1626.77]	107.62 [89.47-132.84]	918.49 [760.09-1134.63]	-1.18 [-1.38 to -0.97]	-1.18
Cameroon	373.02 [289.44-483.68]	1006.53 [788.63-1282.39]	1237.96 [937.77-1665.4]	1178.86 [911.16-1567.65]	0.59 [0.13 to 1.05]	0.59
Eswatini	25.29 [19.94-31.69]	1142.85 [909.52-1409.83]	51.45 [37.12-69.84]	1181.8 [874.51-1558.11]	0.49 [0.07 to 0.91]	0.49
Zimbabwe	271.42 [227.36-317.48]	843.94 [702.5-988.74]	648.57 [530.07-788.32]	1192.86 [985.78-1420.14]	1.65 [1.15 to 2.16]	1.65
Namibia	71.65 [59.52-84.52]	1405.84 [1169.38-1657.61]	131.76 [104.62-161.7]	1202.09 [963.51-1458.95]	-0.74 [-1 to -0.48]	-0.74
Lesotho	64.17 [49.88-81.68]	877.69 [687.23-1123.66]	123.95 [93.38-163.66]	1405.79 [1071.51-1802.76]	2.33 [1.89 to 2.78]	2.33
South Africa	1493.77 [1226.42-1687.16]	784.97 [639.2-890.57]	3596.84 [3201.06-4031.79]	898.42 [797.02-1007.73]	0.49 [0.02 to 0.96]	0.49
Benin	251.15 [205.77-306.32]	1385.23 [1138.09-1689.2]	528.6 [432.55-659.74]	1214.86 [1005.21-1490.42]	-0.39 [-0.5 to -0.29]	-0.39
Cabo Verde	20.48 [16.83-25.3]	865.32 [711.9-1075.27]	44.51 [36.68-53.66]	1057.93 [872.01-1283.11]	0.35 [0.03 to 0.67]	0.35
Chad	299.06 [225.5-415.26]	1146.05 [870.05-1573.49]	654.93 [503.32-885.4]	1321.03 [1014.21-1772.18]	0.41 [0.23 to 0.59]	0.41
Burkina Faso	286.86 [219.81-373.22]	779.37 [605.18-1010.4]	617.73 [489.54-792.41]	777.15 [619.35-995.35]	0.21 [0.11 to 0.31]	0.21
Côte d'Ivoire	466.47 [375.78-574.54]	1421.06 [1169.58-1691.61]	1248.36 [963.64-1635.82]	1318.99 [1042.67-1698.67]	-0.35 [-0.54 to -0.16]	-0.35
Guinea	346.29 [261.11-445.57]	1148.77 [855.2-1475.1]	663.72 [502.74-853.28]	1318.35 [1020.18-1694.29]	0.72 [0.58 to 0.86]	0.72
Gambia	43.35 [33.26-55.14]	1409.96 [1099.92-1779.4]	133.33 [101.82-171.04]	1537.5 [1173.88-1973.2]	0.24 [0.13 to 0.35]	0.24
Guinea-Bissau	64.85 [48.53-85.7]	1864.12 [1430.8-2401.67]	107.65 [83.47-137.53]	1794.2 [1423.26-2249.37]	0.03 [-0.03 to 0.1]	0.03
Liberia	132.48 [108.68-159.26]	1283.68 [1064.4-1533.61]	219.52 [165.96-287.33]	1239.64 [946.33-1604.22]	-0.18 [-0.27 to -0.08]	-0.18
Ghana	919.13 [740.43-1182.35]	1698.57 [1365.74-2146.73]	2562.7 [2023.22-3189.49]	1762.35 [1393.14-2177.09]	0.37 [0.18 to 0.57]	0.37
Mali	313.36 [235.43-433.65]	960.83 [725.63-1318.99]	667.4 [504.28-903.29]	906.34 [698.22-1215.42]	-0.01 [-0.14 to 0.12]	-0.01
Niger	216.22 [152.56-315.48]	971.57 [685.24-1387.11]	615.73 [441.28-868.76]	938.63 [680.95-1298.56]	0.01 [-0.04 to 0.06]	0.01
Togo	151.92 [123.6-185.44]	1437.63 [1180.38-1743.16]	448.35 [334.01-567.19]	1415.57 [1070.74-1774.85]	-0.16 [-0.32 to 0]	-0.16
Nigeria	4735.67 [3464.81-6501.22]	1227.16 [909.75-1665.48]	7448.44 [5915.32-9367.75]	977.51 [784.31-1206.29]	-0.81 [-0.94 to -0.67]	-0.81
Senegal	426.32 [351.87-524.72]	1469.5 [1217.01-1787.12]	886.77 [695.23-1167.33]	1312.66 [1023.76-1717.22]	-0.44 [-0.49 to -0.39]	-0.44
American Samoa	1.37 [1.17-1.6]	733.2 [622.89-846.7]	2.51 [2.11-2.97]	601.5 [504.01-716.02]	-0.89 [-1.03 to -0.75]	-0.89
Sao Tome and Principe	6.83 [5.73-7.96]	1141.47 [959.75-1328.84]	12.56 [10.46-14.99]	1284.3 [1092.04-1525.03]	0.46 [0.33 to 0.6]	0.46
Mauritania	140.25 [107.21-184.09]	1538.88 [1178.32-2019.48]	226.57 [167.08-320.31]	1185.73 [884.47-1653.53]	-0.99 [-1.16 to -0.82]	-0.99
Bermuda	3.92 [3.34-4.51]	668.78 [567.88-766.02]	4.1 [3.43-4.95]	278.09 [232.52-335.31]	-2.91 [-3.13 to -2.68]	-2.91
Sierra Leone	290.99 [233.45-354.18]	1530.98 [1247.13-1843.92]	474.84 [361.89-604.77]	1407.72 [1091.13-1778.3]	-0.07 [-0.23 to 0.09]	-0.07
Cook Islands	0.74 [0.61-0.89]	670.76 [553.92-808.78]	1.13 [0.92-1.38]	447.51 [365.79-546.33]	-1.38 [-1.52 to -1.24]	-1.38
Greenland	3.36 [2.89-3.92]	1396.73 [1193.18-1628.49]	2.66 [2.16-3.26]	496.88 [401.14-611.09]	-3.59 [-3.76 to -3.43]	-3.59
Monaco	8.29 [6.4-10.03]	1012.84 [793.74-1228.27]	4.87 [3.82-5.98]	418.01 [335.61-512.59]	-3 [-3.16 to -2.83]	-3
Nauru	0.6 [0.45-0.75]	1519.35 [1167.11-1875.39]	0.72 [0.57-0.92]	1394.03 [1110.25-1742.67]	-0.43 [-0.72 to -0.14]	-0.43
Niue	0.22 [0.19-0.27]	948.33 [793.93-1138.8]	0.16 [0.14-0.19]	795.37 [664.54-940.39]	-0.79 [-0.87 to -0.72]	-0.79
Guam	3.96 [3.33-4.57]	643.7 [539.73-749.17]	7.31 [6.21-8.71]	351.33 [296.76-418.07]	-1.78 [-2.06 to -1.5]	-1.78
Palau	0.92 [0.73-1.14]	1034.58 [831.73-1277.92]	1.76 [1.45-2.16]	950.45 [791.84-1144.71]	-0.16 [-0.23 to -0.09]	-0.16
Northern Mariana Islands	1.03 [0.83-1.27]	754.38 [608.4-925.33]	2.3 [1.93-2.64]	558.59 [470.64-645.07]	-1.32 [-1.54 to -1.1]	-1.32
Tuvalu	0.63 [0.52-0.77]	1124.91 [931.71-1373.69]	0.83 [0.69-1]	891.79 [734.2-1069.14]	-0.81 [-0.86 to -0.75]	-0.81
United States Virgin Islands	3.9 [3.13-4.64]	560.03 [449.09-666.29]	4.82 [3.86-5.94]	267.74 [216.44-328.33]	-2.24 [-2.38 to -2.1]	-2.24
Puerto Rico	133.78 [116.51-148.84]	387.8 [336.13-432.66]	121.8 [100.09-141.89]	151.09 [125.72-175.36]	-3.42 [-3.61 to -3.22]	-3.42
San Marino	2.45 [2.04-2.87]	652.46 [545.07-763.17]	2.13 [1.58-2.84]	230.9 [174.45-306.17]	-3 [-3.29 to -2.72]	-3
Tokelau	0.12 [0.09-0.15]	915.37 [700.5-1133.86]	0.11 [0.08-0.13]	708.66 [564.95-869.18]	-0.95 [-1.01 to -0.88]	-0.95
Saint Kitts and Nevis	6.71 [5.7-7.6]	1766.27 [1501.25-1999.31]	5.24 [4.3-6.07]	933 [765.15-1066.05]	-1.94 [-2.13 to -1.75]	-1.94
South Sudan	187.89 [136.51-247.6]	835.7 [609.71-1095.22]	223.2 [165.63-298.36]	738.77 [557.77-962.93]	-0.55 [-0.73 to -0.38]	-0.55
Sudan	1534.83 [1106.52-2006.88]	1777.39 [1269.68-2300.34]	2519.15 [1784.59-3373.71]	1395.53 [1010.91-1839.24]	-0.9 [-0.95 to -0.85]	-0.9
East Asia & Pacific - WB	136529.8 [120938.27-155785.97]	1178.45 [1041.53-1344.09]	295271.84 [257516.39-335705.39]	912.76 [795.87-1036.88]	-0.83 [-0.94 to -0.72]	-0.83
Europe & Central Asia - WB	157663.83 [143915.29-168191.02]	1478.84 [1344.59-1580.5]	114768.28 [102428.68-124691.6]	659.24 [592.8-713.5]	-3.11 [-3.35 to -2.87]	-3.11
Latin America & Caribbean - WB	20137.68 [18394.66-21535.81]	834.24 [756.55-893.56]	25512.26 [22704.27-28179.03]	370.53 [329.17-409.37]	-2.63 [-2.75 to -2.5]	-2.63
South Asia - WB	38170.86 [31562.58-47632.34]	760.49 [629.64-939.42]	86849 [74583.11-109481.94]	634.65 [547.15-785.85]	-0.72 [-0.81 to -0.64]	-0.72
Eastern Africa	5413.53 [4582.54-6609.64]	929.68 [788.71-1128.61]	10312.35 [8674.27-12192.42]	767.82 [644.23-906.72]	-0.8 [-0.86 to -0.74]	-0.8
Sub-Saharan Africa - WB	20160.14 [17280.8-24644.43]	1071.5 [920.38-1303.4]	40649.28 [35300.36-46747.44]	978.62 [849.94-1126.57]	-0.32 [-0.44 to -0.2]	-0.32
Middle East & North Africa - WB	18446.13 [15987.86-21705.16]	1705 [1475.82-2004.9]	38376.19 [33432.78-43963.6]	1243.01 [1085.18-1414.79]	-1.04 [-1.08 to -1]	-1.04
Northern Africa	10005.09 [8151.92-12589.92]	1885.37 [1550.09-2350.93]	21139.76 [17414.14-25224.99]	1561.03 [1302.4-1850.27]	-0.42 [-0.51 to -0.33]	-0.42
Southern Africa	3434.04 [3044.74-3856.07]	914.91 [802.74-1031.61]	8224.78 [7282.12-9271.54]	1042.94 [922.33-1175.99]	0.52 [0.21 to 0.83]	0.52
Commonwealth High Income	11882.43 [10700.24-12823.4]	756.45 [680.29-818.79]	6605.38 [5629.13-7542.87]	222.24 [190.13-254.54]	-4.26 [-4.43 to -4.1]	-4.26
Western Africa	8665.55 [6873.84-11317.63]	1254.2 [1002.19-1618.92]	16667.66 [13899.03-19668.9]	1117.27 [938.44-1309.42]	-0.36 [-0.45 to -0.26]	-0.36
African Region	19933.44 [17119.85-24126.37]	1063.38 [913.38-1276.01]	40994.62 [35702.55-46807.07]	972.4 [846.26-1113.64]	-0.32 [-0.44 to -0.2]	-0.32
Commonwealth Middle Income	40560.1 [33696.96-50652.56]	763.56 [633.01-938.41]	89783.13 [78555.57-109614.82]	635.59 [555.88-770.38]	-0.71 [-0.78 to -0.64]	-0.71
Africa	30034.27 [26230.28-34513.85]	1247.55 [1089.66-1434.94]	61605.98 [53649.03-70226.08]	1118.88 [979-1269.33]	-0.31 [-0.41 to -0.21]	-0.31
America	37404.38 [33623.65-40643.41]	622.77 [559.98-677.33]	45251.7 [39872.01-50365.89]	331.56 [291.97-368.89]	-2.28 [-2.43 to -2.13]	-2.28
Commonwealth Low Income	8017.38 [6725.95-9918.63]	1069.67 [894.15-1309.75]	19463.41 [15486.89-24247.84]	993.61 [788.23-1207.07]	-0.28 [-0.42 to -0.14]	-0.28
Asia	188182.7 [167299.45-215023.96]	1079.09 [961.34-1228.56]	406386.32 [358845.22-454286.31]	843.37 [745.01-943.1]	-0.86 [-0.95 to -0.78]	-0.86
Region of the Americas	37404.38 [33623.65-40643.41]	622.77 [559.98-677.33]	45251.7 [39872.01-50365.89]	331.56 [291.97-368.89]	-2.28 [-2.43 to -2.13]	-2.28
Europe	153244.84 [139988.7-163523]	1474.19 [1340.98-1576.11]	108649.05 [96786.52-118284.18]	638 [574.08-690.82]	-3.2 [-3.44 to -2.96]	-3.2
South-East Asia Region	51084.9 [44289.01-60508.65]	860.83 [736.18-1014.66]	121241.6 [107052.27-141336.9]	735.9 [652-851.4]	-0.62 [-0.68 to -0.55]	-0.62
Eastern Mediterranean Region	23761.35 [20551.83-27677.65]	1474.04 [1267-1711.96]	47994.43 [41972.37-54645]	1203 [1053.59-1372.47]	-0.7 [-0.77 to -0.64]	-0.7
European Region	158448.15 [144629.38-169054.7]	1476.58 [1342.42-1578.28]	115779.21 [103372.57-125774.34]	658.44 [592.46-712.92]	-3.11 [-3.35 to -2.87]	-3.11
North America	17408.31 [15211.62-19454.58]	475.01 [414.29-531.33]	19870.17 [16832.51-22725.91]	291.64 [247.57-333.91]	-2.08 [-2.35 to -1.81]	-2.08
Central Africa	2516.08 [1984.23-3130.88]	1105.19 [870.17-1374.54]	5261.44 [4150.79-6816.21]	1017.03 [801.86-1313.73]	-0.35 [-0.45 to -0.25]	-0.35
Western Pacific Region	116865.43 [101980.91-135291.35]	1175.19 [1030.4-1352.21]	249268.54 [214148.09-287405.11]	888.17 [763.91-1024.61]	-0.9 [-1.01 to -0.79]	-0.9
Advanced Health System	190774.23 [173454-204342.69]	1163.39 [1053.84-1248.66]	147861.85 [130376.31-161941.95]	484.23 [431.37-530.31]	-3.32 [-3.53 to -3.11]	-3.32
Basic Health System	154557.43 [137223.32-174859.97]	1195.85 [1066.79-1344.01]	337372.94 [297598.09-383059.12]	955.29 [840.58-1081.42]	-0.71 [-0.83 to -0.58]	-0.71
Minimal Health System	6028.99 [4961.18-7498.07]	1202.43 [991.47-1502.66]	11186.82 [9068.19-14098.76]	1098.56 [897.45-1378.67]	-0.27 [-0.31 to -0.23]	-0.27
Limited Health System	57653.5 [49264.46-70582.51]	858.01 [727.19-1039.96]	125614.29 [109704.12-151380.63]	711.43 [622.1-848.69]	-0.75 [-0.82 to -0.68]	-0.75
Southeast Asia, East Asia, and Oceania	119400.67 [104656.43-138039.46]	1235.08 [1086.96-1423.68]	280979.91 [244524.9-320782.99]	1053.93 [914.65-1200.3]	-0.47 [-0.65 to -0.3]	-0.47
Central Europe, Eastern Europe, and Central Asia	102677.56 [94803.73-108719.25]	2286.15 [2099.27-2428.43]	82474.27 [74408.73-89064.54]	1247.15 [1125.27-1348.12]	-2.61 [-2.97 to -2.24]	-2.61
High-income	91957.78 [82101.29-99610.01]	749.02 [668.36-812.69]	67664.27 [57433.43-76615.47]	273.14 [234.66-310.81]	-3.54 [-3.73 to -3.36]	-3.54
Latin America and Caribbean	16564.49 [15193.11-17663.22]	831.17 [757.69-889.09]	22547.08 [20013.55-24932.31]	375.1 [331.37-414.86]	-2.62 [-2.75 to -2.49]	-2.62
League of Arab States	16531.25 [14114.82-19664.59]	1796.52 [1533.37-2134.35]	35226.16 [29849.98-41089.93]	1443.76 [1231.98-1674.28]	-0.7 [-0.75 to -0.65]	-0.7
World Bank Regions	408516.73 [372133.93-446065.54]	1141.46 [1035.52-1248.09]	621297.01 [556276.24-685295.71]	737.03 [658.17-813.44]	-1.62 [-1.72 to -1.53]	-1.62
WHO region	407497.65 [371147.03-444974.61]	1143.62 [1037.59-1250.58]	620530.1 [555618.34-684419.32]	740.42 [661.2-817.04]	-1.61 [-1.71 to -1.52]	-1.61
Sub-Saharan Africa	18513.72 [15485.82-22731.71]	1037.32 [877.28-1265.39]	38064.83 [33098.57-43791.13]	962.87 [840.4-1111.93]	-0.25 [-0.38 to -0.13]	-0.25
European Union	68854.61 [62730.32-73852.22]	1119.92 [1015.87-1203.89]	42638.49 [37204.06-47323.95]	391.16 [345.2-432.84]	-3.61 [-3.72 to -3.5]	-3.61
Gulf Cooperation Council	1019.94 [804.51-1260.52]	1450.55 [1156.58-1782]	2616.99 [2175.5-3133.6]	956.3 [808.39-1122.77]	-1.4 [-1.59 to -1.21]	-1.4
G20	304800.25 [276823.75-334367.31]	1098.9 [994.33-1205.48]	446101.33 [394325.67-496733.78]	684.3 [604.39-762.24]	-1.75 [-1.85 to -1.65]	-1.75
African Union	30034.27 [26230.28-34513.85]	1247.55 [1089.66-1434.94]	61605.98 [53649.03-70226.08]	1118.88 [979-1269.33]	-0.31 [-0.41 to -0.21]	-0.31
OECD Countries	113044.15 [101771.24-122049.16]	832.08 [747.33-900.05]	85548.66 [73487.94-95997.83]	306.61 [265.34-345.47]	-3.52 [-3.68 to -3.35]	-3.52
Four World Regions	408866.2 [372463.61-446426.06]	1143.1 [1037.08-1249.94]	621893.05 [556821.05-686012.99]	738.24 [659.27-814.67]	-1.62 [-1.71 to -1.53]	-1.62
Commonwealth	60459.92 [52044.8-72130.8]	817.75 [705.1-963.62]	115851.92 [101401.66-139247.91]	611.77 [533.59-728.16]	-1.05 [-1.12 to -0.98]	-1.05
Organization of Islamic Cooperation	60539.41 [54000.98-68225.8]	1331.98 [1181.5-1500.84]	128032.88 [114616.05-142993.87]	1163.53 [1045.06-1289.14]	-0.5 [-0.59 to -0.42]	-0.5
Association of Southeast Asian Nations	25471.31 [22534.68-28427.5]	1208.75 [1063.88-1353.47]	62983.98 [53549.88-72908.74]	1118.51 [961.29-1295.06]	-0.22 [-0.34 to -0.1]	-0.22
Health System Grouping Levels	409014.16 [372602.04-446580.56]	1142.3 [1036.35-1249.07]	622035.89 [556953.95-686178.92]	737.64 [658.74-814]	-1.62 [-1.72 to -1.53]	-1.62
Nordic Region	3093.17 [2763.68-3375.86]	757.65 [678.99-826.57]	1714.32 [1464.43-1955.9]	261.34 [224.95-298.05]	-3.63 [-3.72 to -3.53]	-3.63
Sahel Region	7073.94 [5805.11-9389.85]	1262.56 [1040.88-1666.39]	13005.34 [10912.03-15889.65]	1094.65 [928.5-1317.24]	-0.48 [-0.57 to -0.4]	-0.48

IS-HBMI						
	1990		2021		1990-2021	
	DALY cases	Age-standardized DALY rate per 100,000	DALY cases	Age-standardized DALY rate per 100,000	EAPC of Age-standardized DALY rate	
	No. *102 (95% UI)	No. (95% UI)	No. *102 (95% UI)	No.(95% UI)	No. (95% CI)	
Overall	21258.83 [3051.39-41764.71]	55.3 [7.9-109.16]	44391.86 [6490.3-86474.85]	51.52 [7.52-100.28]	-0.58 [-0.71 to -0.46]	-0.58
Sex						
Female	12447.93 [1790.72-24517.82]	59.38 [8.53-116.34]	23277.77 [3416.68-45069.72]	50.74 [7.46-98.07]	-0.92 [-1.07 to -0.77]	-0.92
Male	8810.89 [1260.67-17089.02]	49.24 [7-96.85]	21114.09 [3072.33-41339.89]	51.98 [7.53-101.35]	-0.1 [-0.21 to 0]	-0.1
Socio-demographic index						
High SDI	5386.05 [777.2-10612.08]	48.79 [7.06-95.71]	6614.36 [969.84-12885.02]	33.8 [5.04-64.29]	-1.49 [-1.66 to -1.32]	-1.49
High-middle SDI	9919.72 [1439.49-19360.31]	102.63 [14.81-200.56]	15363.38 [2242.92-30261]	78.1 [11.41-153.3]	-1.46 [-1.72 to -1.2]	-1.46
Middle SDI	3531.1 [490.17-6919.61]	33.37 [4.6-65.44]	13454.97 [1963.95-26237.79]	49.74 [7.22-96.01]	1.21 [1.17 to 1.26]	1.21
Low-middle SDI	1900.29 [280.32-3756.12]	30.59 [4.47-60.49]	7146.12 [1076.65-13607.57]	48.29 [7.19-93.14]	1.59 [1.54 to 1.65]	1.59
Low SDI	473.95 [58.1-895.87]	20.07 [2.45-37.58]	1757.86 [232.94-3259.93]	32.67 [4.29-60.92]	1.47 [1.42 to 1.53]	1.47
Region						
Andean Latin America	64.16 [9.53-126.7]	29.54 [4.34-59.25]	158.57 [24.25-305.74]	26.41 [4.01-51.13]	-0.59 [-0.79 to -0.39]	-0.59
Australasia	91.31 [12.93-178.37]	39.1 [5.54-76.57]	119.31 [17.56-241.7]	22.18 [3.28-44.18]	-2.05 [-2.19 to -1.9]	-2.05
Caribbean	96.93 [14.44-184.89]	36.95 [5.5-70.72]	242.88 [36.03-471.35]	45.21 [6.7-87.63]	0.74 [0.66 to 0.81]	0.74
Central Asia	593.1 [89-1153.15]	126.02 [18.82-247.56]	1033.42 [156.32-2043.38]	125.81 [18.87-250.74]	-0.55 [-0.89 to -0.21]	-0.55
Central Europe	2438.49 [363.92-4848.05]	165.97 [24.64-328.98]	2238.8 [331.12-4522.57]	99.71 [14.84-199.43]	-2 [-2.15 to -1.85]	-2
Central Latin America	345.07 [52.09-663.61]	40.09 [5.97-78.25]	813.93 [130.27-1574.22]	32.45 [5.16-63.08]	-0.96 [-1.17 to -0.74]	-0.96
Central Sub-Saharan Africa	49.54 [6.01-98.98]	21.91 [2.61-43.43]	243.37 [33.07-479.77]	45.79 [6.2-91.71]	2.24 [2.17 to 2.32]	2.24
East Asia	2192.08 [276.14-4454.21]	24.59 [3.09-49.99]	12111.93 [1690.36-24439.92]	55.34 [7.71-111.28]	2.74 [2.61 to 2.87]	2.74
Eastern Europe	5697.13 [837.72-10890.44]	206.06 [30.14-395.78]	5771.67 [853.41-11066.26]	164.88 [24.41-314.31]	-1.5 [-2 to -1]	-1.5
Eastern Sub-Saharan Africa	117.19 [13.81-219]	15.03 [1.75-27.89]	508 [66.84-965.97]	28.94 [3.77-55.64]	2.04 [2.01 to 2.07]	2.04
High-income Asia Pacific	385.04 [52.03-751.93]	19.55 [2.64-38.18]	492.09 [65.73-951.07]	11.47 [1.55-22.05]	-2.14 [-2.3 to -1.97]	-2.14
High-income North America	1422.87 [207.94-2831.74]	40.67 [5.97-80.27]	2436.98 [362.04-4618.87]	39.47 [5.96-73.95]	-0.55 [-0.77 to -0.33]	-0.55
North Africa and Middle East	2283.86 [356.61-4459.79]	130.72 [20.15-257.78]	7214.9 [1208.32-13615.35]	153.11 [25.18-294.93]	0.47 [0.45 to 0.5]	0.47
Oceania	14.09 [2-27.38]	43.43 [6.07-86.32]	39.03 [5.82-77.97]	47.78 [6.95-98.51]	0.18 [0.11 to 0.25]	0.18
South Asia	518.78 [58.26-1030.11]	8.66 [0.97-16.96]	2964.57 [369.08-5759.86]	19.46 [2.41-37.52]	2.68 [2.64 to 2.72]	2.68
Southeast Asia	488.12 [63.94-929.72]	17.42 [2.28-33.27]	2536.82 [351.26-4911.02]	36.82 [5.03-70.62]	2.54 [2.39 to 2.7]	2.54
Southern Latin America	293.92 [42.66-578.47]	64.2 [9.32-127.16]	330.34 [48.61-662.11]	37.77 [5.58-75.17]	-1.56 [-1.67 to -1.46]	-1.56
Southern Sub-Saharan Africa	162.33 [24.43-309.77]	58.18 [8.69-110.09]	558.64 [83.55-1082.39]	100.52 [14.9-195.52]	1.93 [1.49 to 2.38]	1.93
Tropical Latin America	690.5 [102.19-1332.86]	74.49 [10.9-147.45]	1144.21 [167.8-2250.14]	44.55 [6.52-88.32]	-1.78 [-1.96 to -1.61]	-1.78
Western Europe	2965.53 [420.3-5873.88]	50.59 [7.2-99.38]	2091.61 [296.82-4320.94]	21.83 [3.13-43.88]	-2.91 [-3.08 to -2.75]	-2.91
Western Sub-Saharan Africa	348.79 [44.35-668.61]	39.13 [5-76.48]	1340.8 [187.38-2534.96]	66.5 [9.17-126.68]	1.66 [1.54 to 1.78]	1.66
China	2116.04 [266.09-4302.85]	24.61 [3.09-50.09]	11882.25 [1660.08-23959.96]	56.21 [7.84-113]	2.79 [2.66 to 2.93]	2.79
Cambodia	6.2 [0.82-11.9]	12.88 [1.67-24.99]	25.54 [3.84-49.89]	19.87 [2.91-38.69]	1.26 [1.13 to 1.4]	1.26
Indonesia	173.68 [21.71-345.18]	15.33 [1.92-30.39]	1171.02 [159.55-2312.37]	44.9 [5.97-87.88]	3.76 [3.5 to 4.02]	3.76
Democratic People's Republic of Korea	27.33 [3.41-59.27]	17.8 [2.21-38.56]	136.08 [17.46-302.08]	43.08 [5.49-97.89]	2.82 [2.78 to 2.87]	2.82
Taiwan (Province of China)	48.7 [6.63-93.48]	30.52 [4.12-59.6]	93.6 [12.95-183.03]	23.36 [3.26-45.43]	-0.84 [-1.04 to -0.65]	-0.84
Myanmar	66.87 [9.21-128.75]	26.11 [3.53-50.45]	157.24 [21.78-308.41]	30.33 [4.18-59.65]	0.3 [0.17 to 0.43]	0.3
Lao People's Democratic Republic	5.48 [0.7-11.04]	24.92 [3.25-50.67]	18.56 [2.37-36.95]	37.1 [4.8-74.77]	1.37 [1.31 to 1.43]	1.37
Thailand	70.8 [8.86-136.38]	17.96 [2.25-34.52]	331.66 [51.31-632.55]	31.57 [4.91-60.39]	1.42 [1.16 to 1.67]	1.42
Philippines	59 [7.77-116.67]	18.24 [2.38-35.89]	336.9 [44.44-659.43]	38.11 [5-74.94]	2.54 [2.33 to 2.75]	2.54
Malaysia	39.09 [5.58-73.81]	38.67 [5.54-72.83]	153.79 [21.86-289.57]	52.26 [7.33-99.35]	1.11 [1.01 to 1.22]	1.11
Sri Lanka	29.85 [3.91-58.15]	27.34 [3.55-53.42]	102.82 [14.29-215.91]	39.32 [5.37-82.92]	1.42 [1.22 to 1.62]	1.42
Maldives	0.39 [0.05-0.74]	34.48 [4.34-66.9]	1.16 [0.17-2.26]	27.92 [3.96-54.09]	-1.05 [-1.19 to -0.9]	-1.05
Viet Nam	29.29 [3.7-58.57]	7.31 [0.92-14.61]	223.53 [27.81-446.12]	22.04 [2.75-43.35]	4.35 [4.03 to 4.67]	4.35
Fiji	3.05 [0.45-5.94]	77.72 [11.1-159.11]	7.48 [1.28-13.95]	99.41 [16.29-190.53]	0.55 [0.41 to 0.69]	0.55
Timor-Leste	0.17 [0.02-0.34]	5.12 [0.57-10.7]	1.22 [0.16-2.46]	13.5 [1.71-27.15]	3.55 [3.2 to 3.9]	3.55
Kiribati	0.37 [0.06-0.72]	84.51 [13.39-165.42]	1.03 [0.18-1.91]	123.42 [20.49-236.27]	1.19 [1.12 to 1.25]	1.19
Marshall Islands	0.18 [0.03-0.35]	98.63 [14.33-205.27]	0.46 [0.07-0.9]	120.28 [18.43-239.43]	0.55 [0.48 to 0.62]	0.55
Papua New Guinea	5.23 [0.66-10.96]	25.36 [3.17-54.62]	18.07 [2.54-38.93]	30.52 [4.23-65.07]	0.45 [0.32 to 0.58]	0.45
Samoa	0.84 [0.14-1.6]	92.46 [15.08-177.97]	1.63 [0.28-2.98]	108.73 [18.45-202.54]	0.46 [0.39 to 0.52]	0.46
Micronesia (Federated States of)	0.59 [0.09-1.13]	110.4 [17.44-212.22]	1.05 [0.17-2]	130.03 [20.3-248.06]	0.47 [0.42 to 0.52]	0.47
Solomon Islands	0.76 [0.1-1.56]	48.6 [6.44-102.44]	2.73 [0.4-5.5]	69.74 [10.13-141.99]	1.16 [1.01 to 1.32]	1.16
Tonga	0.38 [0.06-0.69]	64.85 [10.34-117.62]	0.65 [0.11-1.18]	80.83 [13.33-147.45]	0.75 [0.66 to 0.83]	0.75
Armenia	29.89 [4.56-58.45]	112.49 [16.98-224.47]	38.34 [6.09-75.37]	89.3 [14.19-175.87]	-1.63 [-1.96 to -1.31]	-1.63
Vanuatu	0.4 [0.05-0.82]	55.77 [7.31-116.99]	1.47 [0.21-2.95]	75.44 [10.68-157.17]	0.82 [0.75 to 0.89]	0.82
Azerbaijan	37.8 [5.21-77.14]	74.41 [10.27-152.62]	75.4 [11.74-153.65]	73.53 [11.16-149.54]	-0.14 [-0.42 to 0.15]	-0.14
Kazakhstan	218.29 [32.99-434.24]	173.93 [26.14-346.27]	274.33 [40.79-542.42]	160.27 [23.6-321.5]	-0.99 [-1.58 to -0.39]	-0.99
Kyrgyzstan	48.32 [7.35-93.05]	162.4 [24.64-311.99]	73.18 [12.08-139.54]	140.9 [23.08-274.03]	-1.16 [-1.6 to -0.71]	-1.16
Georgia	56.81 [8.4-114.09]	92.82 [13.67-187.89]	77.05 [10.97-152.69]	130.41 [18.7-256.15]	1.02 [0.63 to 1.42]	1.02
Mongolia	3.93 [0.58-7.83]	35.43 [5.18-71.6]	11.12 [1.61-21.52]	43.27 [6.18-85.83]	0.61 [0.27 to 0.96]	0.61
Tajikistan	33.12 [4.82-62.74]	119.8 [17.32-228.5]	64.82 [9.73-127.54]	111.73 [16.7-224.01]	-0.55 [-0.88 to -0.22]	-0.55
Turkmenistan	27.06 [4.18-55.21]	134.44 [20.68-273.37]	77.02 [11.59-156.45]	181.34 [27.17-372.58]	0.79 [0.34 to 1.25]	0.79
Uzbekistan	137.89 [20.67-265.38]	117.14 [17.45-226.84]	342.17 [50.9-681.77]	125.26 [18.54-254.45]	-0.33 [-0.65 to -0.01]	-0.33
Bosnia and Herzegovina	58.6 [8.54-119.18]	145.39 [20.81-301.78]	82.05 [12.46-167.79]	132.08 [20.03-267.89]	-0.68 [-0.82 to -0.54]	-0.68
Albania	11.51 [1.7-23.52]	59.51 [8.77-121.68]	23.37 [3.29-49.58]	54.52 [7.73-115.01]	-0.01 [-0.22 to 0.2]	-0.01
Bulgaria	240.96 [35.56-480.11]	205.92 [30.06-405.1]	306.86 [46.64-626.64]	218.21 [33.39-437.62]	0.28 [0.12 to 0.44]	0.28
Croatia	81.21 [11.57-161.72]	142.83 [20.31-287]	63.3 [9.47-132.29]	68.52 [10.35-140.55]	-2.54 [-2.69 to -2.4]	-2.54
Czechia	296.12 [44.42-589.64]	213.2 [32.07-426.92]	116.74 [17.63-237.93]	54.29 [8.24-109.43]	-4.67 [-4.89 to -4.44]	-4.67
Hungary	306.57 [46.98-592.78]	211.38 [32.42-409.3]	169.04 [25.32-324.35]	89.48 [13.59-170.15]	-3.19 [-3.38 to -3]	-3.19
North Macedonia	40.16 [6.01-77.97]	224.41 [33.3-434.84]	71.42 [10.5-149.27]	236.51 [34.35-491.23]	-0.08 [-0.41 to 0.26]	-0.08
Montenegro	3.33 [0.51-6.78]	54.8 [8.36-112.11]	8.33 [1.15-17.33]	88.76 [12.25-182.94]	1.72 [1.61 to 1.84]	1.72
Poland	603.29 [89.42-1200.66]	140.12 [20.73-277.64]	474.02 [71.77-965.43]	65.37 [9.92-131.02]	-2.83 [-2.94 to -2.71]	-2.83
Romania	423.96 [62.59-827.89]	159.28 [23.15-313.24]	479.07 [66.93-969.81]	125.21 [17.64-252.02]	-1.43 [-1.75 to -1.1]	-1.43
Serbia	208.61 [32.33-430.9]	206.43 [31.62-432.25]	303.88 [45.51-604.25]	178.47 [26.92-353.56]	-1.1 [-1.39 to -0.8]	-1.1
Slovakia	97.51 [14.28-190.68]	163.2 [23.85-316.92]	90.7 [13.62-177.03]	94.43 [14.16-183.15]	-1.87 [-1.98 to -1.76]	-1.87
Slovenia	27.66 [4-54.09]	112.85 [16.42-221.01]	17.43 [2.48-36.6]	37.54 [5.34-78.3]	-3.65 [-3.88 to -3.42]	-3.65
Estonia	39.69 [5.91-76.21]	193.54 [28.73-372.22]	13.42 [1.88-26.87]	52.58 [7.45-102.97]	-5.53 [-6.04 to -5.01]	-5.53
Belarus	198.98 [28.9-394.04]	152.04 [21.99-301.01]	234.67 [34.28-452.13]	145.03 [21.27-278.68]	-0.98 [-1.52 to -0.44]	-0.98
Latvia	74.03 [11.42-147.55]	206.96 [31.85-413.07]	57.45 [8.35-116.41]	141.32 [20.71-280.76]	-1.75 [-2.02 to -1.48]	-1.75
Lithuania	53.12 [8.05-105.75]	118.3 [17.99-236.16]	56.16 [8.06-114.38]	99.06 [14.35-198.09]	-0.5 [-0.86 to -0.15]	-0.5
Republic of Moldova	43.38 [6.62-84.2]	104.18 [15.71-204.06]	74.71 [11.38-141.26]	123.81 [18.93-234.05]	0.77 [0.26 to 1.27]	0.77
Russian Federation	3917.6 [579.32-7536.36]	220.98 [32.4-427.04]	4170.61 [613.75-8150.7]	174.96 [25.75-341.12]	-1.61 [-2.19 to -1.03]	-1.61
Ukraine	1370.33 [197.46-2619.65]	192.12 [27.74-367.43]	1164.66 [188.66-2279.17]	152.09 [24.76-297.81]	-1.4 [-1.71 to -1.09]	-1.4
Brunei Darussalam	0.39 [0.05-0.74]	32.43 [4.44-61.36]	1.28 [0.19-2.4]	31.59 [4.56-60.21]	-0.16 [-0.26 to -0.05]	-0.16
Republic of Korea	93.68 [12.65-194.83]	32.56 [4.4-68.11]	142.67 [19.48-284.28]	15.64 [2.14-31.01]	-3.06 [-3.3 to -2.82]	-3.06
Japan	285.77 [38.21-548.04]	17.26 [2.31-33.16]	338.06 [44.68-654.17]	10.26 [1.37-19.54]	-2 [-2.16 to -1.83]	-2
Singapore	5.2 [0.68-9.98]	22.31 [2.9-43.24]	10.07 [1.43-19.49]	11.7 [1.65-22.48]	-2.47 [-2.68 to -2.27]	-2.47
Australia	73.84 [10.4-145.48]	38.05 [5.37-75.02]	98.69 [14.57-200.83]	21.77 [3.23-43.36]	-2.04 [-2.18 to -1.9]	-2.04
New Zealand	17.46 [2.53-34.4]	44.38 [6.41-86.78]	20.63 [2.98-41.37]	24.46 [3.57-48.04]	-2.05 [-2.25 to -1.85]	-2.05
Andorra	0.13 [0.02-0.27]	23.68 [3.55-49.43]	0.24 [0.04-0.49]	15.42 [2.34-30.85]	-1.29 [-1.5 to -1.08]	-1.29
Austria	64.33 [8.91-131.76]	53.4 [7.45-108.16]	37.13 [5.06-78.51]	20.16 [2.79-41.07]	-3.35 [-3.69 to -3.02]	-3.35
Belgium	57.46 [8.2-111.74]	37.41 [5.38-72.37]	44.18 [6.27-89.03]	18.49 [2.64-36.96]	-2.28 [-2.43 to -2.13]	-2.28
Cyprus	2.78 [0.41-5.38]	42.75 [5.97-86.05]	4.12 [0.63-8.59]	22.64 [3.45-47.63]	-2.48 [-2.67 to -2.29]	-2.48
Finland	42.89 [6.12-86.57]	59.98 [8.57-120.26]	34.48 [4.93-69.27]	26.71 [3.84-52.69]	-2.66 [-2.75 to -2.56]	-2.66
France	213.55 [30.41-415.22]	25.34 [3.62-49.24]	245.38 [33.99-503.21]	16.77 [2.41-32.77]	-1.25 [-1.33 to -1.18]	-1.25
Denmark	31.28 [4.45-61.19]	38.22 [5.46-74.4]	22.72 [3.23-46.45]	18.96 [2.73-38.23]	-2.59 [-2.76 to -2.42]	-2.59
Germany	873.89 [123.28-1717.77]	68.3 [9.71-132.7]	550.07 [76.82-1120.72]	29.33 [4.13-58.22]	-2.82 [-3.07 to -2.57]	-2.82
Greece	93.75 [13.06-191.51]	62.54 [8.74-127.46]	83.92 [12.2-179.69]	31.52 [4.63-64.24]	-2.84 [-3.11 to -2.57]	-2.84
Ireland	18.98 [2.75-38.34]	46.25 [6.7-91.88]	12.44 [1.77-25.82]	15.7 [2.24-32.18]	-3.75 [-3.97 to -3.53]	-3.75
Israel	17.41 [2.56-34.7]	36.26 [5.32-72.17]	20.4 [2.94-41.2]	16.3 [2.37-32.99]	-3.08 [-3.26 to -2.9]	-3.08
Iceland	1.22 [0.18-2.43]	42.03 [6.19-83.38]	1.09 [0.15-2.21]	18.27 [2.59-36.97]	-2.82 [-2.93 to -2.71]	-2.82
Luxembourg	3.73 [0.51-7.29]	68.84 [9.47-133.97]	1.95 [0.29-4]	17.69 [2.65-35.72]	-4.5 [-4.65 to -4.36]	-4.5
Italy	363.01 [52.42-716.35]	40.66 [5.86-80.73]	284.39 [39.71-592.15]	17.31 [2.45-35.73]	-3.09 [-3.39 to -2.79]	-3.09
Malta	1.7 [0.24-3.27]	40.23 [5.6-76.79]	1.59 [0.23-3.32]	16.26 [2.39-33.19]	-3.18 [-3.41 to -2.95]	-3.18
Norway	30.18 [4.21-59.55]	42.79 [5.99-84.11]	16.38 [2.27-32.34]	16.07 [2.24-31.47]	-3.52 [-3.63 to -3.41]	-3.52
Netherlands	73.66 [10.29-148.61]	36.55 [5.11-73.73]	74.2 [10.84-149.95]	21.13 [3.1-42.59]	-2.21 [-2.43 to -1.99]	-2.21
Portugal	130.73 [17.58-262.83]	95.96 [12.83-195.1]	69.92 [9.76-149.53]	26.13 [3.78-54.78]	-4.81 [-5.04 to -4.57]	-4.81
Spain	299.36 [41.91-603.09]	55.04 [7.72-109.88]	226.99 [33.44-480.51]	22.24 [3.29-45.35]	-3.05 [-3.3 to -2.79]	-3.05
Sweden	57.34 [8.07-117.06]	36.93 [5.21-73.83]	39.76 [5.55-81.77]	18.41 [2.59-36.28]	-2.51 [-2.65 to -2.37]	-2.51
United Kingdom	553.33 [79.78-1116.22]	59.61 [8.63-119.53]	295.48 [42.09-606.19]	22.51 [3.24-45.01]	-3.46 [-3.63 to -3.29]	-3.46
Switzerland	31.69 [4.48-65.52]	29.17 [4.14-59.11]	22.4 [3.07-46.82]	11.63 [1.62-23.21]	-2.94 [-3.15 to -2.74]	-2.94
Argentina	194.15 [28.18-378.96]	60.69 [8.82-118.96]	203.45 [29.99-411.37]	36.15 [5.35-72.53]	-1.5 [-1.66 to -1.34]	-1.5
Chile	68.63 [10-136.32]	69.37 [10.07-138.16]	99.88 [14.63-195.96]	39.1 [5.74-76.34]	-1.59 [-1.73 to -1.45]	-1.59
Uruguay	31.13 [4.48-61.5]	80.22 [11.55-157.89]	26.99 [3.99-55.37]	48.46 [7.25-97.2]	-1.88 [-2.05 to -1.7]	-1.88
Canada	119.11 [17.31-239.27]	36.82 [5.35-73.56]	169.04 [24.5-345.11]	24.68 [3.63-48.78]	-1.63 [-1.81 to -1.45]	-1.63
United States of America	1303.37 [190.57-2593.26]	41.12 [6.04-81.01]	2267.59 [337.49-4291.06]	41.25 [6.24-76.92]	-0.46 [-0.68 to -0.23]	-0.46
Bahamas	0.76 [0.12-1.48]	47.36 [7.26-92.65]	1.92 [0.29-3.62]	47.48 [7.07-89.91]	0.03 [-0.07 to 0.13]	0.03
Antigua and Barbuda	0.24 [0.04-0.49]	45.34 [6.85-89.65]	0.46 [0.07-0.9]	44.13 [6.78-88.14]	-0.27 [-0.47 to -0.07]	-0.27
Belize	0.34 [0.05-0.68]	35.74 [5.32-71.5]	1.32 [0.22-2.48]	43.81 [7.17-84.19]	0.35 [-0.17 to 0.87]	0.35
Barbados	1.79 [0.26-3.49]	62.42 [9.13-120.97]	3.11 [0.49-6.27]	60.74 [9.68-121.74]	-0.24 [-0.39 to -0.09]	-0.24
Cuba	32.92 [5.05-61.64]	32.17 [4.92-60.13]	85.63 [12.66-166.36]	44.58 [6.64-86.64]	1.22 [1.11 to 1.33]	1.22
Dominican Republic	10.56 [1.44-20.94]	26.48 [3.61-52.81]	49.19 [7.67-97.4]	48.19 [7.51-95.3]	2.15 [1.99 to 2.31]	2.15
Grenada	0.56 [0.08-1.09]	81.86 [11.93-161.01]	0.65 [0.09-1.25]	57.39 [7.98-112.88]	-0.89 [-1.12 to -0.66]	-0.89
Dominica	0.44 [0.06-0.86]	74.65 [10.72-145.62]	0.71 [0.11-1.37]	86.54 [13.72-169.65]	0.45 [0.34 to 0.56]	0.45
Guyana	4.19 [0.6-8.23]	106.49 [14.9-209.68]	5.68 [0.87-11.28]	88.21 [13.4-174.83]	-0.01 [-0.23 to 0.21]	-0.01
Saint Lucia	0.62 [0.09-1.19]	71.72 [10.66-139.69]	1.21 [0.19-2.41]	51.23 [7.78-102.34]	-1.45 [-1.79 to -1.1]	-1.45
Haiti	10.39 [1.31-20.78]	29.38 [3.68-59.92]	35.24 [4.45-72.59]	44.48 [5.61-92.54]	1.65 [1.52 to 1.78]	1.65
Saint Vincent and the Grenadines	0.29 [0.04-0.55]	40.67 [5.62-77.75]	0.62 [0.09-1.21]	44.47 [6.68-87.17]	0.29 [0.12 to 0.45]	0.29
Jamaica	9.85 [1.41-18.81]	54.71 [7.83-103.85]	19.06 [3.04-37.79]	60.79 [9.72-119.37]	0.62 [0.24 to 1]	0.62
Trinidad and Tobago	6.77 [1.04-12.53]	80.4 [12.29-151.44]	11.76 [1.74-22.98]	61.39 [9.03-118.91]	-1.38 [-1.62 to -1.13]	-1.38
Suriname	0.92 [0.13-1.83]	35.35 [5-70.73]	2.93 [0.44-5.84]	45.94 [6.91-91.47]	0.73 [0.44 to 1.02]	0.73
Bolivia (Plurinational State of)	12.68 [1.73-25.98]	37.47 [5.08-77.57]	32.41 [4.71-68.21]	35.23 [5-74.91]	-0.29 [-0.42 to -0.15]	-0.29
Ecuador	22.79 [3.4-44.56]	39.73 [5.86-78.49]	51.14 [7.69-98.5]	31.31 [4.67-60.24]	-0.65 [-0.97 to -0.33]	-0.65
Peru	28.7 [4.31-59.62]	22.9 [3.41-48.22]	75.02 [11.55-148.08]	21.85 [3.35-43.22]	-0.69 [-1.07 to -0.29]	-0.69
Colombia	64.15 [9.28-123.95]	35.75 [5.15-69.53]	128.63 [19.35-260.76]	23.25 [3.5-47.18]	-1.93 [-2.28 to -1.57]	-1.93
Costa Rica	4.78 [0.7-9.45]	26.5 [3.84-52.57]	12.74 [1.99-25.29]	23.11 [3.62-45.79]	-0.9 [-1.22 to -0.57]	-0.9
El Salvador	10.39 [1.51-20.3]	33.74 [4.85-65.86]	18.83 [2.81-37.54]	30.11 [4.51-59.67]	-0.62 [-0.96 to -0.28]	-0.62
Guatemala	10.8 [1.7-20.45]	30.46 [4.71-57.81]	27.97 [4.28-54.87]	25.3 [3.83-50.55]	-1.08 [-1.38 to -0.77]	-1.08
Honduras	8.48 [1.13-17.01]	39.88 [5.36-82.16]	40.97 [5.92-83.07]	65.88 [9.48-136.51]	1.78 [1.62 to 1.95]	1.78
Panama	6.31 [0.9-12.82]	43.59 [6.18-89.15]	17.16 [2.75-34.22]	38.42 [6.18-76.72]	-0.58 [-0.76 to -0.4]	-0.58
Venezuela (Bolivarian Republic of)	38.73 [6.06-78.23]	39.17 [6.05-80.23]	124.23 [19.24-245.41]	42.56 [6.52-84.5]	-0.13 [-0.43 to 0.18]	-0.13
Nicaragua	5.86 [0.89-11.41]	36.56 [5.5-70.91]	15.22 [2.47-29.84]	30.9 [4.98-61.05]	-0.58 [-0.7 to -0.47]	-0.58
Mexico	195.58 [29.44-377.25]	44.12 [6.54-85.63]	428.19 [70.19-804.63]	33.61 [5.46-64.11]	-1.07 [-1.24 to -0.9]	-1.07
Brazil	679.71 [100.54-1312.1]	75.11 [10.99-148.72]	1115.06 [163.54-2190.7]	44.42 [6.5-87.95]	-1.82 [-2 to -1.65]	-1.82
Algeria	99.52 [14.02-206]	82.4 [11.7-169.64]	386.23 [62.55-771.35]	111.47 [17.89-224.2]	0.87 [0.83 to 0.92]	0.87
Paraguay	10.79 [1.59-21.33]	49.22 [7.22-98.79]	29.14 [4.26-59.49]	51.04 [7.38-105.02]	0.12 [0 to 0.23]	0.12
Bahrain	1.9 [0.29-3.63]	101.88 [14.79-198.75]	8.65 [1.52-16.57]	96.21 [16.31-193.15]	-0.84 [-1.19 to -0.49]	-0.84
Egypt	671.39 [105.17-1401.59]	251.92 [38.5-532.36]	2189.26 [395.37-4055.25]	346.95 [59.76-665.42]	1.38 [1.22 to 1.54]	1.38
Iran (Islamic Republic of)	264.21 [39.13-495.13]	93.14 [13.69-176.81]	797.59 [126.85-1507.55]	99.59 [15.68-189.53]	0.01 [-0.13 to 0.16]	0.01
Iraq	199.56 [29.89-383]	240.56 [35.67-470.05]	583.88 [97.24-1144.57]	240.41 [38.76-480.48]	-0.49 [-0.64 to -0.35]	-0.49
Jordan	28.29 [4.42-53.44]	203.07 [31-391.88]	107.23 [18.15-201.3]	141.67 [23.56-278]	-1.76 [-2.14 to -1.38]	-1.76
Lebanon	22.85 [3.13-46.96]	104.65 [14.13-216.56]	39.04 [6.22-75.57]	64.03 [10.29-122.23]	-1.73 [-2.07 to -1.39]	-1.73
Kuwait	5.74 [0.9-10.39]	73.14 [11.13-138.21]	29.68 [5.05-54.07]	81.23 [13.15-153.44]	0.58 [-0.29 to 1.45]	0.58
Libya	15.08 [2.31-30.22]	74.34 [11.22-149.96]	95.12 [15.83-178.81]	163.89 [26.49-309.35]	2.91 [2.73 to 3.09]	2.91
Morocco	150.23 [19.94-322.69]	100.82 [13.35-217.77]	530.92 [80.12-1045.03]	150.64 [22.67-299.57]	1.41 [1.32 to 1.49]	1.41
Palestine	15.73 [2.32-32.08]	183.58 [26.95-381.21]	40.97 [7.05-75.02]	167.14 [28.17-319.64]	-0.35 [-0.57 to -0.12]	-0.35
Oman	7.31 [1.01-14.67]	91.16 [12.58-186.45]	30.77 [5.56-54.28]	124.78 [21.54-233.55]	1.53 [1.33 to 1.73]	1.53
Qatar	1.42 [0.23-2.73]	112.31 [18.01-238.68]	9.74 [1.73-18.03]	76.49 [12.89-151.21]	-1.6 [-2.18 to -1.03]	-1.6
Tunisia	32.33 [4.74-67.54]	63.03 [9.2-131.36]	127.48 [20.11-259.91]	96.47 [15.12-198.78]	1.23 [1.12 to 1.33]	1.23
Saudi Arabia	91.22 [13.61-178.69]	140.57 [20.41-280.61]	466.63 [85.95-863.6]	187.31 [32.41-352.83]	0.79 [0.61 to 0.97]	0.79
Turkey	369.19 [52.43-708.74]	106.36 [14.92-206.73]	764.74 [122.63-1481.63]	83.33 [13.31-163.69]	-1.03 [-1.26 to -0.81]	-1.03
Syrian Arab Republic	70.13 [10.98-129.49]	123.19 [19.3-232.1]	217.9 [36.61-420.2]	165.69 [27.13-325.52]	0.46 [0.2 to 0.72]	0.46
United Arab Emirates	7.58 [1.16-14.44]	126.26 [18.51-247.39]	64.54 [11.6-114.69]	140.43 [22.68-262.11]	1.39 [1 to 1.78]	1.39
Yemen	35.81 [4.63-72.07]	67.24 [8.75-138.58]	200.91 [26.69-395.79]	132.06 [17.53-260.86]	2.08 [1.93 to 2.23]	2.08
Afghanistan	79.55 [10.34-156.84]	108.63 [14.19-214.52]	171.28 [27.7-363.79]	147.56 [22.83-316.97]	0.84 [0.7 to 0.97]	0.84
Nepal	10.07 [1.19-20.08]	9.55 [1.12-19.12]	39.17 [4.78-80.73]	15.8 [1.92-32.94]	1.74 [1.55 to 1.93]	1.74
Bangladesh	40.85 [4.96-85.59]	8.38 [0.99-17.7]	293.43 [39.5-617.79]	20.8 [2.8-44.18]	3.45 [3.29 to 3.62]	3.45
Pakistan	96.22 [11.41-203.39]	16.44 [1.95-35.04]	551.48 [77.37-1093.15]	42.65 [5.87-85.16]	3.25 [3.05 to 3.44]	3.25
Bhutan	0.72 [0.1-1.47]	27.31 [3.92-55.34]	2.08 [0.32-4.47]	33.57 [5.07-72.12]	0.63 [0.57 to 0.7]	0.63
India	370.92 [40.96-728.95]	7.65 [0.84-15.18]	2078.42 [251.67-4038.18]	17.01 [2.06-32.92]	2.6 [2.53 to 2.66]	2.6
Congo	4.18 [0.48-8.41]	36.36 [4.12-74]	19.38 [2.64-37.35]	68.92 [9.11-137.39]	1.74 [1.55 to 1.92]	1.74
Democratic Republic of the Congo	31.26 [3.61-64.59]	20.24 [2.29-41.81]	153.13 [19.89-322.91]	44.57 [5.74-94.69]	2.48 [2.39 to 2.57]	2.48
Equatorial Guinea	0.79 [0.1-1.58]	38.64 [5.2-79.67]	3.61 [0.5-7.01]	70.08 [9.48-137.27]	1.64 [1.41 to 1.87]	1.64
Gabon	3.03 [0.38-5.74]	52.87 [6.72-98.87]	8.67 [1.3-16.54]	84.99 [12.36-160.98]	1.36 [1.17 to 1.55]	1.36
Angola	7.83 [0.99-15.27]	18.77 [2.35-37.42]	49.45 [6.67-97.4]	40.14 [5.42-79.58]	2.2 [2.07 to 2.33]	2.2
Central African Republic	2.45 [0.28-4.89]	20.28 [2.39-40.83]	9.14 [1.17-19.52]	39.43 [5.07-86.36]	2.09 [2.04 to 2.14]	2.09
Burundi	4.24 [0.47-8.87]	17.73 [1.98-37.59]	8.21 [1.06-16.4]	16.44 [2.06-32.88]	-0.93 [-1.26 to -0.6]	-0.93
Comoros	0.5 [0.06-1.01]	24.95 [3.02-50.88]	2.07 [0.29-4.06]	42.01 [5.87-82.4]	1.43 [1.24 to 1.63]	1.43
Djibouti	0.15 [0.02-0.32]	10.09 [1.14-21.78]	1.29 [0.16-2.55]	18.57 [2.27-37.25]	1.82 [1.75 to 1.89]	1.82
Eritrea	1.25 [0.13-2.57]	10.74 [1.16-22.67]	5.09 [0.7-10.31]	17.8 [2.4-37.41]	1.64 [1.47 to 1.8]	1.64
Kenya	12.01 [1.51-22.91]	13.56 [1.7-25.98]	74.08 [9.87-141.84]	30.6 [3.96-58.34]	2.85 [2.74 to 2.95]	2.85
Ethiopia	20.35 [2.13-42.41]	9.37 [0.99-19.36]	48.93 [6.29-95.07]	10.72 [1.36-20.99]	0.05 [-0.12 to 0.22]	0.05
Madagascar	10.09 [1.29-19.27]	19.92 [2.55-39.13]	44.03 [5.93-87.28]	39.56 [5.44-79.79]	2.11 [2 to 2.21]	2.11
Malawi	6.06 [0.7-11.54]	15.51 [1.75-29.8]	29.19 [3.72-57.02]	38 [4.83-74.3]	2.65 [2.43 to 2.86]	2.65
Mauritius	6.27 [0.87-11.84]	80.31 [11.11-153.9]	9 [1.32-17.45]	49.86 [7.3-95.36]	-2.62 [-3.14 to -2.1]	-2.62
Mozambique	12.7 [1.64-25.03]	20.25 [2.58-40.77]	68.53 [9.26-138.95]	56.25 [7.58-113.62]	3.83 [3.64 to 4.01]	3.83
Seychelles	0.32 [0.05-0.61]	56.74 [8.12-106.94]	0.84 [0.13-1.57]	70.68 [11.03-130.58]	0.91 [0.79 to 1.04]	0.91
Rwanda	6.65 [0.87-12.94]	22.36 [2.89-43.18]	13.31 [1.8-27.17]	20.72 [2.77-42.75]	-1.25 [-1.71 to -0.78]	-1.25
Somalia	4.57 [0.52-9.76]	15.7 [1.68-32.63]	15.73 [1.94-33.4]	21.46 [2.52-45.71]	0.94 [0.84 to 1.04]	0.94
United Republic of Tanzania	21.59 [2.53-41.86]	19.47 [2.27-38.17]	121.05 [16.56-239.66]	47.63 [6.43-96.56]	2.86 [2.68 to 3.04]	2.86
Uganda	9.28 [1.24-18.25]	13.57 [1.8-27.11]	34.38 [4.72-68.07]	21.37 [2.92-42.41]	0.85 [0.59 to 1.12]	0.85
Zambia	5.36 [0.67-10.64]	18.07 [2.2-36.23]	36.81 [5.31-73.23]	51.74 [7.18-105.27]	3.25 [3.14 to 3.37]	3.25
Botswana	2.74 [0.35-5.35]	51.58 [6.57-102.23]	10.58 [1.45-19.96]	78.49 [10.55-152.36]	1.6 [1.3 to 1.89]	1.6
Eswatini	2.21 [0.3-4.11]	88.87 [11.7-171.69]	7.69 [1.04-15.22]	156.35 [20.79-317.17]	2.22 [1.72 to 2.72]	2.22
Lesotho	3.97 [0.59-8.05]	48.91 [7.25-101.53]	12.61 [1.97-25.77]	123.9 [19.01-249.76]	3.98 [3.44 to 4.53]	3.98
Zimbabwe	9.7 [1.41-18.47]	23.59 [3.44-44.99]	56.77 [8.31-110.67]	81.78 [11.69-157.15]	4.78 [4.12 to 5.46]	4.78
Namibia	3.28 [0.45-6.53]	54.34 [7.24-114.59]	12.26 [1.75-23.62]	98.98 [14.17-193.9]	1.74 [1.43 to 2.05]	1.74
South Africa	140.43 [21.28-269.82]	65.11 [9.76-125.08]	458.72 [69.63-885.96]	102.2 [15.38-197.47]	1.54 [1.1 to 1.99]	1.54
Cabo Verde	0.66 [0.09-1.28]	29.98 [3.87-58.24]	3.13 [0.5-6.06]	68.05 [10.73-132.41]	2.49 [2.26 to 2.72]	2.49
Benin	9.75 [1.27-19.01]	47.6 [6.15-94.79]	35.67 [5.04-72.52]	66.19 [9.28-134.79]	1.04 [0.89 to 1.19]	1.04
Chad	7.73 [0.97-15.92]	27.17 [3.41-56.55]	26.79 [3.45-55.77]	44.76 [5.77-93.71]	1.56 [1.43 to 1.69]	1.56
Burkina Faso	5.13 [0.72-10.34]	10.98 [1.53-22.14]	17.64 [2.23-36.15]	17.26 [2.17-36.13]	1.62 [1.54 to 1.71]	1.62
Cameroon	25.67 [3.2-50.75]	56.67 [7.12-114.35]	142.3 [21.17-282.23]	110.36 [16.2-223.42]	2.24 [1.77 to 2.71]	2.24
Côte d'Ivoire	22.58 [2.92-43.83]	50.21 [6.4-96.72]	101.11 [13.56-197.94]	81.26 [10.88-160.56]	1.48 [1.28 to 1.68]	1.48
Guinea	10.79 [1.33-21.13]	31.61 [3.91-61.84]	33.24 [4.52-65.05]	55.96 [7.55-111.02]	2.14 [2.05 to 2.24]	2.14
Guinea-Bissau	2.11 [0.28-4.34]	49.38 [6.53-100.23]	6.8 [0.99-13.46]	84.39 [12.05-165.12]	1.86 [1.82 to 1.9]	1.86
Gambia	1.95 [0.25-3.79]	50.97 [6.55-99.44]	9.02 [1.21-18.37]	86.92 [11.57-174.78]	1.62 [1.46 to 1.78]	1.62
Liberia	7.76 [1.03-15.38]	63.98 [8.47-127.6]	23.95 [3.53-47.3]	102 [14.85-200.48]	1.43 [1.33 to 1.53]	1.43
Ghana	32.63 [4.1-63.5]	46.28 [5.82-89.77]	197.47 [27.16-382.56]	110.48 [14.84-213.8]	3 [2.83 to 3.18]	3
Mali	9.94 [1.24-20.81]	24.13 [2.94-50.49]	28.61 [3.77-57.2]	30.12 [3.99-60.06]	0.78 [0.58 to 0.97]	0.78
Niger	6.56 [0.75-13.72]	22.34 [2.56-47.63]	23.17 [2.98-49.14]	26.97 [3.39-58.33]	0.61 [0.53 to 0.69]	0.61
Nigeria	165.83 [21.44-335.75]	38.26 [4.97-78.19]	560.09 [78.81-1065.21]	62.52 [8.64-122.06]	1.41 [1.26 to 1.57]	1.41
Sao Tome and Principe	0.34 [0.04-0.68]	51.89 [6.74-103.9]	1.16 [0.17-2.19]	98.57 [13.92-188.75]	2 [1.89 to 2.11]	2
Senegal	17.04 [2.25-32.41]	49.85 [6.53-94.99]	53.25 [7.47-102.34]	66.41 [9.29-128.6]	0.83 [0.76 to 0.91]	0.83
Mauritania	9.22 [1.25-18.67]	91.58 [12.42-186.51]	22.69 [3.18-47.64]	106.28 [14.68-220.4]	0.29 [0.13 to 0.44]	0.29
American Samoa	0.24 [0.04-0.42]	93.22 [15.23-170.55]	0.45 [0.08-0.79]	93.9 [16.14-169.07]	-0.24 [-0.38 to -0.1]	-0.24
Bermuda	0.35 [0.05-0.7]	56.04 [8.28-113.8]	0.47 [0.08-0.95]	35.09 [5.83-68.69]	-1.57 [-1.81 to -1.32]	-1.57
Sierra Leone	7.71 [1-15.18]	36.78 [4.7-71.83]	25.45 [3.39-49.68]	62.82 [8.17-123.96]	1.94 [1.84 to 2.05]	1.94
Cook Islands	0.11 [0.02-0.21]	84.07 [12.57-159.88]	0.17 [0.03-0.33]	69.83 [11.73-133.81]	-0.62 [-0.78 to -0.46]	-0.62
Togo	5.38 [0.67-10.38]	41.1 [5.14-81.24]	29.24 [4.09-56.49]	73.81 [10.06-144]	1.81 [1.72 to 1.9]	1.81
Greenland	0.36 [0.05-0.7]	117.03 [16.8-237.22]	0.31 [0.05-0.62]	49.14 [7.69-100.53]	-3.05 [-3.19 to -2.9]	-3.05
Monaco	0.53 [0.07-1.06]	72.51 [9.63-142.31]	0.39 [0.06-0.81]	38.16 [6.12-76.41]	-2.21 [-2.35 to -2.07]	-2.21
Nauru	0.11 [0.02-0.19]	189.85 [30.64-341.66]	0.16 [0.03-0.29]	234.11 [39.55-433.07]	0.5 [0.14 to 0.85]	0.5
Guam	0.52 [0.08-1]	62.68 [9.25-122.59]	1.03 [0.16-2.01]	51.22 [8.17-98.95]	-0.6 [-0.83 to -0.38]	-0.6
Niue	0.02 [0-0.04]	88.91 [13.62-174.69]	0.02 [0-0.04]	105.77 [17.32-205.66]	0.32 [0.24 to 0.4]	0.32
Palau	0.13 [0.02-0.25]	122.99 [18.99-233.74]	0.31 [0.05-0.6]	138.44 [21.26-269.52]	0.44 [0.39 to 0.5]	0.44
Northern Mariana Islands	0.19 [0.03-0.35]	83.41 [13.1-160.21]	0.41 [0.07-0.76]	79.65 [12.61-151.08]	-0.44 [-0.64 to -0.24]	-0.44
Tuvalu	0.07 [0.01-0.14]	97.44 [14.04-192.98]	0.13 [0.02-0.24]	119.7 [18.8-228.94]	0.66 [0.61 to 0.72]	0.66
United States Virgin Islands	0.46 [0.08-0.89]	56.88 [9.22-112.29]	0.56 [0.09-1.13]	33.53 [5.39-66.6]	-1.62 [-1.78 to -1.47]	-1.62
Puerto Rico	11.77 [1.8-23.55]	33.23 [5.08-66.42]	13.51 [2.14-26.7]	20.15 [3.24-38.86]	-2.05 [-2.25 to -1.85]	-2.05
San Marino	0.15 [0.02-0.3]	41.48 [6.13-83.51]	0.16 [0.02-0.35]	21.09 [3.07-43.5]	-1.91 [-2.15 to -1.67]	-1.91
Tokelau	0.01 [0-0.02]	85.77 [13.14-173.36]	0.01 [0-0.03]	94.85 [15.62-188.76]	0.19 [0.12 to 0.26]	0.19
Saint Kitts and Nevis	0.42 [0.06-0.84]	112.73 [15.33-223.6]	0.62 [0.1-1.19]	93.12 [14.48-183.27]	-0.6 [-0.8 to -0.41]	-0.6
South Sudan	2.3 [0.27-4.69]	8.72 [1.04-17.83]	4.87 [0.6-9.71]	11.91 [1.44-24.39]	0.83 [0.5 to 1.15]	0.83
Sudan	113.57 [15.2-235.79]	114.39 [15.47-237.95]	345.63 [53.52-704.96]	158.1 [24.32-318.63]	0.94 [0.9 to 0.98]	0.94
East Asia & Pacific - WB	3135.98 [402.47-6091.19]	23.15 [2.97-45.81]	15190.97 [2116.67-30051.59]	45.87 [6.39-90.68]	2.3 [2.22 to 2.38]	2.3
Latin America & Caribbean - WB	1486.94 [220.54-2893.37]	55.32 [8.14-108.33]	2681.21 [406.95-5226.77]	38.02 [5.76-74.46]	-1.33 [-1.49 to -1.17]	-1.33
Europe & Central Asia - WB	11959.15 [1748.31-23257.03]	112.17 [16.4-217.72]	11761.59 [1746.02-23201.06]	73.08 [10.92-141.27]	-1.97 [-2.25 to -1.68]	-1.97
South Asia - WB	628.57 [72.89-1226.42]	10.16 [1.17-19.75]	3239.84 [411.2-6213.57]	20.73 [2.61-39.82]	2.35 [2.3 to 2.39]	2.35
Sub-Saharan Africa - WB	797.77 [106.8-1489.71]	35.42 [4.73-67.37]	3004.54 [424.19-5763.97]	59.63 [8.32-114.99]	1.63 [1.47 to 1.78]	1.63
Eastern Africa	208.92 [28.96-401.29]	28.49 [3.94-55.15]	720.29 [106.71-1416.08]	42.26 [6.14-85.77]	1.07 [1.01 to 1.13]	1.07
Middle East & North Africa - WB	1739.56 [271.19-3406.31]	135.87 [20.94-268.46]	5949.79 [996.48-11096.7]	165.52 [27.14-314.5]	0.64 [0.59 to 0.68]	0.64
Northern Africa	977.76 [153.83-2004.58]	154.64 [24.09-320.91]	3351.69 [571.79-6150.59]	211.74 [35.29-399.73]	1.22 [1.12 to 1.33]	1.22
Southern Africa	194.28 [28.29-365.46]	43.87 [6.34-83.77]	742.62 [108.11-1427.94]	81.35 [11.76-158.15]	2.09 [1.71 to 2.48]	2.09
Commonwealth High Income	784.12 [113-1573.31]	51.15 [7.39-102.09]	619.61 [89.4-1270.74]	23.19 [3.38-46.28]	-2.85 [-3 to -2.7]	-2.85
Western Africa	305.82 [39.04-589.65]	37.93 [4.86-74.34]	1147.85 [160.69-2185.3]	63.57 [8.74-121.24]	1.59 [1.5 to 1.68]	1.59
Commonwealth Middle Income	952.34 [119.11-1807.92]	15.12 [1.89-28.77]	4397.82 [589.17-8292.18]	27.6 [3.68-51.82]	1.94 [1.82 to 2.05]	1.94
Africa	1766.46 [265.22-3496.32]	61.22 [9.11-122.42]	6334.83 [993.28-12090.24]	95.66 [14.7-183.55]	1.54 [1.42 to 1.65]	1.54
America	2897.19 [426.53-5696.28]	47.34 [6.96-93.16]	5103.77 [766.71-9822.67]	38.37 [5.79-73.38]	-0.99 [-1.14 to -0.83]	-0.99
Commonwealth Low Income	116.84 [14.19-222.94]	12.85 [1.56-24.53]	659.42 [89.34-1341.23]	27.49 [3.7-55.94]	2.58 [2.52 to 2.64]	2.58
Asia	4997.6 [665.84-9647.55]	24.29 [3.23-47.3]	21878.55 [3083.76-42049.08]	42.73 [6-82.63]	1.79 [1.74 to 1.84]	1.79
Region of the Americas	2897.19 [426.53-5696.28]	47.34 [6.96-93.16]	5103.77 [766.71-9822.67]	38.37 [5.79-73.38]	-0.99 [-1.14 to -0.83]	-0.99
Europe	11536.35 [1684.62-22413.8]	111.1 [16.23-215.47]	11003.09 [1628.84-21653.59]	70.36 [10.5-136.2]	-2.06 [-2.34 to -1.77]	-2.06
African Region	779.15 [103.81-1466.99]	34.74 [4.63-66.07]	3029.41 [429.46-5819.66]	59.9 [8.42-116.25]	1.7 [1.54 to 1.86]	1.7
South-East Asia Region	791.66 [94.62-1533.72]	10.51 [1.25-20.21]	4314.3 [560.09-8334.19]	23.04 [2.97-44.37]	2.59 [2.5 to 2.69]	2.59
Eastern Mediterranean Region	1914.85 [301.28-3773.5]	101.23 [15.78-201.77]	6625.69 [1092.72-12437.66]	139.38 [22.46-267.4]	1.06 [1 to 1.13]	1.06
European Region	12018.08 [1757.06-23376.05]	111.98 [16.37-217.37]	11854.69 [1759.31-23400.4]	72.89 [10.89-140.95]	-1.97 [-2.25 to -1.68]	-1.97
North America	1422.82 [207.93-2831.64]	40.66 [5.97-80.26]	2437.11 [362.06-4619.1]	39.46 [5.96-73.95]	-0.55 [-0.77 to -0.33]	-0.55
Central Africa	79.68 [9.66-150.03]	28.24 [3.41-53.52]	372.38 [51.42-709.92]	57.03 [7.76-110.04]	2.25 [2.08 to 2.43]	2.25
Western Pacific Region	2747.61 [352.1-5490.43]	23.83 [3.06-47.85]	13298.44 [1850.67-26631.22]	46.92 [6.53-93.87]	2.28 [2.2 to 2.35]	2.28
Advanced Health System	13519.67 [1971.6-26400.9]	82.66 [12.06-161.04]	14603.82 [2168.96-28329.93]	53.35 [8.02-102.05]	-2 [-2.25 to -1.75]	-2
Basic Health System	6221.17 [888.17-12233.95]	41.43 [5.88-81.44]	23313.62 [3462.64-45159.75]	61.78 [9.14-120.11]	1.29 [1.24 to 1.34]	1.29
Minimal Health System	198.06 [25.25-374.81]	31.63 [4.01-60.62]	633.32 [89.03-1198.69]	47.51 [6.59-91.36]	1.24 [1.18 to 1.29]	1.24
Limited Health System	1272.22 [160.07-2435.08]	15.62 [1.96-29.82]	5785.92 [766.78-10942.35]	28.33 [3.73-53.83]	1.88 [1.83 to 1.92]	1.88
Southeast Asia, East Asia, and Oceania	2694.29 [342.08-5283.66]	22.97 [2.91-45.16]	14687.78 [2048.17-29104.94]	51.36 [7.14-101.82]	2.73 [2.6 to 2.86]	2.73
Central Europe, Eastern Europe, and Central Asia	8728.73 [1290.63-16764.63]	185.8 [27.34-360.26]	9043.89 [1340.85-17464.16]	139.01 [20.64-268.19]	-1.6 [-1.99 to -1.21]	-1.6
High-income	5158.65 [735.85-10113.79]	43.04 [6.16-84.19]	5470.33 [790.75-10740.46]	25.93 [3.81-49.45]	-1.92 [-2.11 to -1.74]	-1.92
Latin America and Caribbean	1196.67 [178.43-2298.81]	52.91 [7.81-103.69]	2359.59 [359.64-4589.17]	37.99 [5.77-74.34]	-1.23 [-1.41 to -1.06]	-1.23
WHO region	21148.52 [3035.52-41532.12]	55.35 [7.91-109.26]	44226.31 [6466.46-86157.42]	51.67 [7.54-100.56]	-0.58 [-0.7 to -0.45]	-0.58
Sub-Saharan Africa	677.85 [87.83-1278.53]	31.78 [4.13-60.41]	2650.8 [364.87-5018.11]	55.56 [7.59-106.17]	1.77 [1.59 to 1.95]	1.77
World Bank Regions	21170.81 [3038.27-41593.92]	55.16 [7.88-108.89]	44265.05 [6471.88-86244.72]	51.43 [7.51-100.09]	-0.58 [-0.71 to -0.45]	-0.58
European Union	4572.58 [663.07-9026.82]	76.06 [11.07-149.41]	3577.43 [517.75-7273.27]	37.2 [5.44-74.71]	-2.56 [-2.66 to -2.46]	-2.56
League of Arab States	1584.11 [250.67-3148.14]	146.09 [22.82-292.79]	5516.32 [929.33-10353.74]	187.92 [30.83-357.4]	0.84 [0.79 to 0.9]	0.84
G20	15250.19 [2168.65-29893.66]	51.91 [7.36-101.96]	29628.05 [4239.45-57596.01]	45.64 [6.54-88.63]	-0.8 [-0.94 to -0.65]	-0.8
African Union	1766.46 [265.22-3496.32]	61.22 [9.11-122.42]	6334.83 [993.28-12090.24]	95.66 [14.7-183.55]	1.54 [1.42 to 1.65]	1.54
Four World Regions	21197.59 [3042.21-41645.62]	55.26 [7.9-109.08]	44320.24 [6479.46-86338.94]	51.52 [7.52-100.29]	-0.58 [-0.71 to -0.45]	-0.58
OECD Countries	7051.35 [1018.06-13907.54]	52.68 [7.62-103.71]	7549.07 [1122.52-14827.94]	30.93 [4.66-59.06]	-2.04 [-2.21 to -1.86]	-2.04
Commonwealth	1853.31 [246.35-3601.62]	23.31 [3.11-45.13]	5676.84 [762.03-10697.67]	27.54 [3.69-52.03]	0.5 [0.45 to 0.56]	0.5
Organization of Islamic Cooperation	3489.93 [521.63-6931.91]	65.52 [9.72-131.09]	11564.94 [1817.02-21894.04]	88.03 [13.6-170.44]	0.87 [0.79 to 0.94]	0.87
Association of Southeast Asian Nations	456.01 [59.71-871.14]	16.84 [2.21-32.07]	2429.58 [336.14-4714.23]	36.27 [4.94-69.64]	2.6 [2.45 to 2.76]	2.6
Nordic Region	163.28 [23.08-324.43]	42.98 [6.09-84.5]	114.74 [16.19-233.29]	19.99 [2.84-39.72]	-2.7 [-2.78 to -2.61]	-2.7
Gulf Cooperation Council	115.17 [17.38-223.73]	128.55 [18.89-255.2]	610 [111.72-1109.76]	154.04 [26.34-292.01]	0.51 [0.27 to 0.75]	0.51
Health System Grouping Levels	21211.12 [3044.3-41671.15]	55.24 [7.89-109.04]	44336.68 [6482.08-86369.73]	51.5 [7.52-100.24]	-0.58 [-0.71 to -0.45]	-0.58
Sahel Region	284.38 [38.59-543.72]	42.79 [5.78-82.14]	971.94 [137.28-1855.71]	64.63 [9-126.27]	1.23 [1.17 to 1.3]	1.23

### 3.2 Disease burden of IS-HBMI by different age groups in 1990 to 2021

The global burden of disease for IS-HBMI in 2021 showed significant age stratification, and although the absolute number of deaths was concentrated in the older age groups, with the 70–74-year-olds (421,000), 65–69-year-olds (359,000), and 75–79-year-olds (358,000) together accounting for 65.8% of the total number of deaths and constituting the core burden group, the pattern of the age burden varied across regions.

The age-specific mortality rate (ASMR) increased markedly with age, from 1.21 per 100,000 in the 20-24-year-old group to 287.45 per 100,000 in the 95 and over group (237.5-fold increase), and regional differences widened with age, with the ratio of the ASMR in high SDI to low SDI areas decreasing from 0.87 in the 20–24-year-old group to 0.23 (p<0.001)

The analysis of time trends showed a pattern of ‘fast decline in old age and slow decline in young age’. Time trend analysis showed that the EAPC showed a pattern of ‘fast decline in the old age and slow decline in the young age’: the young age group (20-29 years old) had a slow decline (EAPC=-0.32 to -0.41), the old age group (70-79 years old) had a significant decline (EAPC=-2.15 to -2.08), and the group of 60-69 years old had a stable trend (EAPC=-0.05 to 0). The magnitude of this age-based disparity in EAPC varied across different regions and Socio-demographic Index (SDI) strata.

### 3.3 Disease burden of IS-HBMI by different regions from 1990 to 2021

The global burden of IS-HBMI in 2021 exhibited significant disparities aligned with socioeconomic development. Stratification by Sociodemographic Index (SDI) revealed clear gradients in the age-standardized mortality rate (ASMR), ranging from 1.12 per 100,000 in high-SDI regions to 2.34 per 100,000 in low-SDI regions. More strikingly, the temporal trends from 1990 to 2021 diverged substantially: high-SDI regions experienced the most pronounced decline, with an estimated annual percentage change (EAPC) of -2.60% (95% UI: -2.79 to -2.40). In stark contrast, the ASMR in low-SDI regions increased, with an EAPC of +1.54%  (95% CI: 1.40 to 1.68).

Analysis at the GBD super-region level further highlights extreme divergence. Western Europe (ASMR=1.45 per 100,000, EAPC=-2.71%) and High-income Asia Pacific (ASMR=1.38 per 100,000, EAPC=-2.60%) exemplify a “low-burden, steady-decline” pattern. In stark contrast, several regions, particularly in Central Asia, Eastern Europe, and Oceania, sustained the highest burdens (ASMR > 3.0 per 100,000) with stagnant or increasing trends. For instance, Central Asia had the highest ASMR (6.23 per 100,000) and exhibited an increasing trend (EAPC = +1.54%). China’s experience as a leading nation in the middle SDI quintet is noteworthy. It achieved one of the fastest declines in ASMR globally, with an EAPC of -2.85% from 1990 to 2021. This rate of improvement surpasses the global average (EAPC = -1.10%) and is more substantial than the average decline within its own SDI group (Middle SDI quintile average EAPC=-1.47%) This progress has contributed to a significant reduction in China's share of the global IS-HBMI death count, demonstrating the potential for  concerted national action to be associated with  effective curbing of the disease burden.

### 3.4 Projection of IS-HBMI disease burden from 2022 to 2050

Projections for 2022 to 2050 suggest that the global IS-HBMI age-specific mortality rate (ASMR) will end a period of rapid decline and enter a plateau of near-zero increases, but this general plateau hides growing health inequalities between regions.

Specifically, ASMR in high socio-demographic index (SDI) regions (e.g., Western Europe) is projected to decline steadily from 0.89/100,000 [0.12-1.89] in 2021, while China, as a model for medium-high SDI regions, is projected to experience a significant decline in ASMR from approximately 2.21/100,000 to 1.25/100,000 through active interventions. However, the outlook for low-SDI regions (e.g., sub-Saharan Africa) is extremely grim, with the ASMR projected to have risen from 4.43 to 5.81 per 100,000.

At the same time, age-stratified projections reveal distinct future trajectories: the middle-aged group (45-64 years) will see a steady decline in burden (e.g., from 35/100,000 to 20/100,000 in the 55-59 year old group), which is a reliable focus for prevention and control; the young group (20-44 years) has low mortality, but very wide confidence intervals (e.g., 95% UI: 0.2-0.6/100,000 in the 30-34 year old group) suggesting great uncertainty associated with the younger age group for obesity; and the elderly group (≥65 years) will see limited improvement (e.g., less than 25% in the 80-84 year old group), whose high and persistent burden will continue to challenge healthcare systems. and limited improvement (e.g., less than 25% reduction in the 80-84 age group) in the older age group (≥65 years), whose high and persistent burden will continue to challenge the healthcare system.

In summary, the future global burden of IS-HBMI is shifting from a generalized improvement to an intensification of differentiation, with low SDI regions emerging as new growth poles and the accumulation of risk early in the lifecycle posing a long-term threat that urgently requires more targeted and equitable global prevention and control strategies. A more targeted and equitable global prevention and control strategy is urgently needed.

## 4 Discussion

This study provides a comprehensive analysis and projection of the global burden of disease for IS-HBMI.The global burden of IS-HBMI has continued to increase from 1990 to 2021 [29]. Our analysis confirms that, despite global progress in ASMR, the absolute number of deaths from IS-HBMI continues to rise, constituting a growing global public health burden [30], [31]. This divergence between ‘rising absolute numbers and falling standardized rates’ reflects the complex interplay of population growth, ageing and epidemiological shifts.

One of the most striking findings of this study is the existence of large inequalities in IS-HBMI burden, which are manifested in three main dimensions: socioeconomic, sex, and age. First, socioeconomic inequality was the central driver. The large gap in ASMR decline between high and low SDI regions (EAPC: -2.60% vs. +1.54%) directly contributes to the dramatic increase in global health inequality. High SDI regions, with their well-established healthcare systems, advanced screening technologies and high public health literacy, have succeeded in significantly reducing the risk of age-standardized [32]. In contrast, low-SDI regions, particularly in southern sub-Saharan Africa, are facing a ‘double burden of disease’ of malnutrition and obesity epidemics, and their health systems are weakly equipped to cope with them, keeping the burden of IS-HBMI high and even increasing [33], [34]. This SDI gradient is futher pronounced at the regional and national levels. For instance, the high-income Asia Pacific region maintained a low ASMR (1.38 per 100,000) alongside a steady decline (EAPC: -2.60%), whereas regions like Central Asia bore a high and increasing burden (ASMR: 6.23 per 100,000, EAPC: +1.54%). This stark contrast highlights the strong association between socioeconomic development and the disease burden trajectory [35], [36]. Second, sex differences were significant and regionally heterogeneous. Men consistently bore a higher risk of IS-HBMI throughout the study period (significantly higher ASMR than women), but women outperformed men in the rate of improvement in ASMR, especially in high SDI areas, and the rate of ASMR decline was steeper among women (EAPC: -1.34%) than among men (EAPC: -0.92%). The reasons for this sex asymmetry in mortality trends are likely multifactorial and may include  differential access to or effectiveness of healthcare, variations in risk factor profiles, and biological differences [37], [38]. This sex asymmetry in prevention and control outcomes suggests the need to consider multi-level sex-specific factors. However, given the ecological design of our study, these population-level disparities cannot definitively elucidate individual-level biological causality. They instead invite hypotheses that can be informed by mechanistic research.  Beyond social dimensions such as healthcare accessibility and health behaviors, emerging basic research indicates that the intrinsic molecular pathophysiological responses following ischemic stroke also exhibit sex heterogeneity. For instance, a study examining enhanced RNA (eRNA) expression in the post-stroke cerebral cortex revealed that eRNA expression patterns exhibit both sex-dependent and sex-independent characteristics. Key molecules such as eRNA_06347 show high induction in both sexes, and functional loss of this molecule leads to a significant increase in infarct volume in both males and females [39]. Thus, one plausible hypothesis is that at the level of epigenetic regulation, that both sexes share common foundational biological response mechanisms to stroke while also possessing distinct pathways, which may contribute to the observed epidemiological disparities. Consequently, future intervention strategies must integrate these insights: for men, intensifying management of prominent risk factors and alleviating work-related stress; for women, focusing on and leveraging their unique protective or susceptibility pathways [40]. Third, age-stratified analyses reveal the transfer of risk over the life cycle. Third, age-stratified analyses reveal risk shifts over the life cycle. While the older age group remains at the centre of the absolute burden, the trend of a slow decline in ASMR and even an increase in risk in the younger age group (20-29 years) is particularly worrying [41]. This implies that the long-term metabolic risks associated with exposure to high BMI in early life stages are accumulating and may set the stage for future disease outbreaks [42], [43]. The significant improvement in the burden in the older age group is evidence of the effectiveness of current interventions for this population. At the same time, the significant improvement in the burden in the older age group is evidence of the effectiveness of current interventions targeting this population, but its high and persistent absolute burden will continue to challenge the health-care system [44], [45].

The experience of China warrants consideration as a case study  for intermediate SDI regions. Its accelerated ASMR reduction is temporally associated with  the ambitious, multisectoral “Healthy China 2030”  initiative. While ecological data cannot establish causation, this alignment indicates that  integrating NCD control into national development planning is a feasible strategy, providing a reference point  for other countries [46].

Frontier analysis indicated that low-SDI regions had the largest deviation from the optimal performance curve, suggesting substantial potential for burden reduction through improved health system efficiency.

The results of forecast modelling for the future to 2050 are a wake-up call. Global ASMR enters a plateau, masking growing regional divergence [47]. It is crucial to interpret these projections with caution, as they are contingent upon the continuation of past trends and do not account for unforeseen major public health interventions or socioeconomic disruptions. The wide uncertainty interval, particularly for younger age groups, reflect the inherent unpredictability of long-term risk factor evolution and healthcare access in different regions. Low-SDI regions are projected to be the new pole of burden growth, while the burden in high-SDI regions will continue to decline, leading to a further deepening of the global health divide by nearly 70 per cent. In addition, the wide confidence intervals of the projections for the youth group hint at the great uncertainty associated with the rejuvenation of obesity, posing a potential threat in the long term [48]. In summary, the findings of this study highlight the need to move from a ‘one-size-fits-all’ universal strategy to a precise and stratified global prevention and control.

Taken together, the findings of this study highlight the need to shift from a one-size-fits-all universal strategy to a precise, stratified global prevention and control approach to address the challenge of IS-HBMI. For high SDI regions, the focus should be on sustaining existing gains and intervening in adolescent obesity; for medium and high SDI regions, policy-driven multisectoral collaboration needs to be strengthened to control metabolic risks associated with economic development; and for low SDI regions, the international community should prioritise assistance in establishing an integrated prevention, screening, and treatment system with low cost. For low SDI regions, the international community should give priority to assisting them in establishing an integrated ‘prevention-screening-treatment’ and low-cost early prevention and control system, so as to prevent IS-HBMI from becoming a new challenge that is beyond their capacity [49]. Taken together, the findings of this study highlight the need to shift from a one-size-fits-all universal strategy to a precise, stratified global prevention and control approach to address the challenge of IS-HBMI. Future research should aim to elucidate the long-term effects of high BMI exposure in adolescence and develop optimized prevention and control programmes based on cost-effectiveness to ultimately contribute to an equitable decline in the global burden of IS-HBMI.

This requires strengthening primary health care systems as the foundational platform for delivering equitable obesity prevention services and managing complications—which are core components of primary and first-line stroke prevention. Strategies must be stratified according to the Socioeconomic Development Index (SDI) to ensure cost-effectiveness and feasibility. In areas with higher SDI scores, the focus should be on maintaining integrated care models and addressing adolescent obesity. In regions with moderate and upper-moderate Socioeconomic Development Indices (SDIs), emulating policy-driven multisectoral collaboration models is crucial for curbing metabolic risks during periods of economic transition. For regions with low Socioeconomic Development Indices (SDIs), the international community’s priority must be to establish basic primary healthcare capacity, ensure the supply of affordable essential medicines, and integrate simple screening and management protocols into existing community health platforms.

### 4.1 Limitations/future directions

This study has several limitations. First, our findings are subject to uncertainties inherent to the Global Burden of Disease (GBD) methodological framework.  Although the 2021 Global Burden of Disease report represents the most comprehensive and recent data source for global comparative assessments, its estimates rely on the availability and quality of underlying data, which vary significantly across countries. Second, the precision of GBD estimates ultimately depends on the availability and quality of the underlying source data, which vary significantly across countries Potential underreporting or misclassification in regions with low social development indices may affect the precision of disease burden estimates and trend changes. Third, methodological assumptions introduce limitations.  The BAPC model assumes that historical age, period, and cohort effects will persist. Third, while substantial sex and regional disparities were observed, these differences are based on ecological data, and their underlying biological or behavioral mechanisms require further investigation at the individual level.

Future research should focus on several key areas. First, with the release of updated Global Burden of Disease data, our predictive models require timely updates and validation. Second, there is an urgent need for individual-level cohort studies to elucidate the life course effects of early high BMI exposure and to uncover the biological basis for sex differences in metabolic syndrome risk. Third, cost-effectiveness analyses of targeted interventions are crucial for resource allocation. Finally, implementation research is urgently needed to develop and validate scalable, low-cost prevention and management strategies for underserved populations in low socioeconomic development regions, ensuring equitable translation of global goals into local action. This study aligns with the World Health Organization's goal to reduce premature mortality from noncommunicable diseases by one-third by 2030, underscoring the imperative to address obesity as a key driver of the noncommunicable disease epidemic. The pronounced gradient revealed by the Social Development Index (SDI) indicates that achieving health equity among resource-deprived populations requires localizing successful strategies from high-resource settings.

## 5 Conclusion

This study reveals a persistent and growing absolute burden of IS-HBMI from 1990 to 2021, coupled with profound and widening inequalities across socioeconomic, sex, and age dimensions. Projections to 2050 signal a troubling stagnation in global progress and a dramatic amplification of the health gap between high- and low-SDI regions. These findings compel a shift from uniform strategies to precision public health actions: In high-SDI regions, efforts must sustain gains and aggressively address the emerging threat of adolescent obesity through primary care and community-based programs; In middle- and high-middle-SDI regions, adopting the core principles of integrated, policy-driven approaches is crucial to embed metabolic risk factor control within expanding primary care systems.; In low-SDI regions, projected to the new epicenters of disease burden, the international community must prioritize support to build foundational primary care capacity, ensuring access to low-cost “prevention-screening-management” for hypertension, diabetes, and obesity to avert a crisis.

Addressing the rising burden of IS-HBMI is integral to achieving global NCD reduction targets. Future success depends on equitable, context-specific strategies that prioritize the most vulnerable populations.

## CRediT authorship contribution statement

**Shuting Ni:** Data curation. **Ying Zhang:** Formal analysis. **Shilei Lin:** Software. **Yongxiang Zhang:** Data curation. **Tong Zhao:** Supervision, Software.

## Ethics

Ethical approval was not required for this study as it utilized publicly available, de-identified aggregate data from the Global Burden of Disease Study 2021.

## Funding

This work was supported by grants from the 10.13039/501100001809National Natural Science Foundation of China (No.82301543), Joint Funds for the Innovation of Science and Technology, Fujian Province (No.2024Y9123).

## Declaration of competing interest

The authors declare that they have no known competing financial interests or personal relationships that could have appeared to influence the work reported in this paper.

## Data Availability

The data that support the findings of this study are openly available from the Global Burden of Disease Study 2021 (IHME). The analysis code is available from the corresponding author upon reasonable request. The analysis code for this study has been permanently archived on Zenodo (https://doi.org/10.5281/zenodo.19333054). The data retrieval and analysis scripts include modules for data cleaning, statistical analysis, and visualization, utilizing R version 4.3.0 environments. Date accessed: March 30, 2026.
